# Multi-omic association study identifies DNA methylation-mediated genotype and smoking exposure effects on lung function in children living in urban settings

**DOI:** 10.1371/journal.pgen.1010594

**Published:** 2023-01-13

**Authors:** Matthew Dapas, Emma E. Thompson, William Wentworth-Sheilds, Selene Clay, Cynthia M. Visness, Agustin Calatroni, Joanne E. Sordillo, Diane R. Gold, Robert A. Wood, Melanie Makhija, Gurjit K. Khurana Hershey, Michael G. Sherenian, Rebecca S. Gruchalla, Michelle A. Gill, Andrew H. Liu, Haejin Kim, Meyer Kattan, Leonard B. Bacharier, Deepa Rastogi, Matthew C. Altman, William W. Busse, Patrice M. Becker, Dan Nicolae, George T. O’Connor, James E. Gern, Daniel J. Jackson, Carole Ober

**Affiliations:** 1 Department of Human Genetics, University of Chicago, Chicago Illinois, United States of America; 2 Rho Inc., Durham, North Carolina, United States of America; 3 Department of Population Medicine, Harvard Medical School, Boston, Massachusetts, United States of America; 4 Department of Environmental Health, Harvard T.H. Chan School of Public Health, Boston, Massachusetts, United States of America; 5 Channing Division of Network Medicine, Brigham and Women’s Hospital, Harvard Medical School, Boston, Massachusetts, United States of America; 6 Department of Pediatrics, Johns Hopkins University Medical Center, Baltimore, Maryland, United States of America; 7 Division of Allergy and Immunology, Ann & Robert H. Lurie Children’s Hospital, Chicago, Illinois, United States of America; 8 Department of Pediatrics, University of Cincinnati College of Medicine, Cincinnati, Ohio, United States of America; 9 Division of Asthma Research, Cincinnati Children’s Hospital Medical Center, Cincinnati, Ohio, United States of America; 10 Department of Internal Medicine, University of Texas Southwestern Medical Center, Dallas, Texas, United States of America; 11 Department of Pediatrics, Washington University School of Medicine, St. Louis, Missouri, United States of America; 12 Department of Allergy and Immunology, Children’s Hospital Colorado, University of Colorado School of Medicine, Aurora, Colorado, United States of America; 13 Department of Medicine, Henry Ford Health System, Detroit, Michigan, United States of America; 14 Columbia University College of Physicians and Surgeons, New York, New York, United States of America; 15 Monroe Carell Jr. Children’s Hospital at Vanderbilt University Medical Center, Nashville, Tennessee, United States of America; 16 Children’s National Health System, Washington, District of Columbia, United States of America; 17 Department of Allergy and Infectious Diseases, University of Washington, Seattle, Washington, United States of America; 18 Department of Pediatrics and Medicine, University of Wisconsin School of Medicine and Public Health, Madison, Wisconsin, United States of America; 19 National Institute of Allergy and Infectious Diseases, Bethesda, Maryland, United States of America; 20 Department of Statistics, University of Chicago, Chicago, Illinois, United States of America; 21 Pulmonary Center, Boston University School of Medicine, Boston, Massachusetts, United States of America; University of Minnesota School of Public Health, UNITED STATES

## Abstract

Impaired lung function in early life is associated with the subsequent development of chronic respiratory disease. Most genetic associations with lung function have been identified in adults of European descent and therefore may not represent those most relevant to pediatric populations and populations of different ancestries. In this study, we performed genome-wide association analyses of lung function in a multiethnic cohort of children (n = 1,035) living in low-income urban neighborhoods. We identified one novel locus at the *TDRD9* gene in chromosome 14q32.33 associated with percent predicted forced expiratory volume in one second (FEV_1_) (p = 2.4x10^-9^; β_z_ = -0.31, 95% CI = -0.41- -0.21). Mendelian randomization and mediation analyses revealed that this genetic effect on FEV_1_ was partially mediated by DNA methylation levels at this locus in airway epithelial cells, which were also associated with environmental tobacco smoke exposure (p = 0.015). Promoter-enhancer interactions in airway epithelial cells revealed chromatin interaction loops between FEV_1_-associated variants in *TDRD9* and the promoter region of the *PPP1R13B* gene, a stimulator of p53-mediated apoptosis. Expression of *PPP1R13B* in airway epithelial cells was significantly associated the FEV_1_ risk alleles (p = 1.3x10^-5^; β = 0.12, 95% CI = 0.06–0.17). These combined results highlight a potential novel mechanism for reduced lung function in urban youth resulting from both genetics and smoking exposure.

## Author summary

Lung function is determined by both genetic and environmental factors. Impairment of lung function can result from harmful environmental exposures in early life, which disproportionally affect children living in low-income, urban communities. However, most genetic association studies of lung function have been performed in adults and without regard for socioeconomic status. Therefore, genetic risk factors discovered to date may not reflect those most relevant to high-risk populations. In this study, we sought to identify genetic variants correlated with lung function in a multiethnic cohort of children living in low-income, urban neighborhoods and analyze how tobacco smoke exposure may influence any genetic effects. We discovered a common genetic variant associated with lower lung function in this population, and we found that the association was mediated by nearby epigenetic changes in DNA methylation, which were in turn correlated with smoking exposure. We then identified a nearby gene, *PPP1R13B*, which is known to aid in the deactivation of damaged cells, whose expression in airway cells aligned with these genetic and epigenetic effects. This study reveals a potential mechanism through which genetic risk and environmental exposures can affect airway development, perhaps leading to interventions that can help reduce the burden of asthma in socioeconomically disadvantaged children.

## Introduction

Reduced lung function is a hallmark of asthma and chronic obstructive pulmonary disease (COPD). Lung function measures, such as forced expiratory volume in one second (FEV_1_) and forced vital capacity (FVC), are strong predictors of future all-cause mortality [1–6]. Airway obstruction often begins in early life [7–10], with lower lung function in infancy being a risk factor for the development of asthma in childhood [11] and COPD in late adulthood [12].

Genetic factors contribute to differences in lung function among individuals, with heritability estimates ranging from 0.50 for FEV_1_ to 0.66 for FEV_1_/FVC ratio [13]. The many genome-wide association studies (GWAS) of lung function measures [14–25] have implicated pathways related to lung development [20,26–28], inflammation [26], and tissue repair [29], among others [29]. Lung function is also affected by environmental exposures, such as smoking [30–32] and air pollution [33], which can disrupt airway development in early life, increasing the risk of childhood asthma and perhaps other chronic obstructive diseases [8–12,34,35]. For example, exposure to second hand smoke in utero and through childhood is associated with increased risk of childhood asthma [36], lower lung function in adolescence [37], and larger declines in lung function later in life [38,39]. Such adverse exposures are known to alter the epigenetic landscape in exposed individuals [40,41], potentially mediating downstream biological effects [42–44] and modifying genetic associations with lung function [45,46].

Environmental risk factors disproportionally affect socioeconomically disadvantaged children, particularly those living in urban environments [47,48]. In fact, socioeconomic effects contribute to disparities in lung health [49], including the higher burden of chronic respiratory disease among Black and Hispanic children compared to non-Hispanic white children [49–52]. Most genetic association studies of lung function, however, have been limited to adults of European descent. Therefore, genetic risk factors discovered to date may not reflect those most relevant to high-risk populations, which can further exacerbate health disparities [53,54]. Identifying genetic variants and epigenetic variation associated with lung function in high-risk, multiethnic, pediatric populations may provide more direct insights into the early development of impaired lung function.

In this study, we analyzed measures of lung function from the Asthma Phenotypes in the Inner City (APIC) [55,56] and Urban Environment and Childhood Asthma (URECA) cohorts [57], which consist of children living in low-income neighborhoods in 10 U.S. cities. We performed whole-genome sequencing (WGS) on 1,035 participants from APIC and URECA (ages 5–17 years; 67% non-Hispanic Black, 25% Hispanic; 66% with doctor-diagnosed asthma) and performed a GWAS with FEV_1_ and the FEV_1_/FVC ratio. We then performed expression quantitative trait locus (eQTL) and methylation quantitative trait locus (meQTL) mapping in airway epithelial cells and peripheral blood mononuclear cells (PBMCs) from a subset of the URECA children. We further tested for genotype and DNA methylation interactions with smoking exposure. We aimed to identify methylation-mediated genetic and smoking exposure associations with lung function, linking environmental effects, epigenetic modifications, and specific genetic risk alleles to reduced pulmonary health in urban youth.

## Results

### Genetic variants at the *TDRD9* locus are associated with lung function

We completed WGS and variant calling on 1,035 participants from the APIC and URECA studies (APIC = 508, URECA = 527; Table 1). The mean sequencing depth was 31.6x per sample (S1A Fig). On average, 95.3%, 90.3% and 62.6% of each genome was mapped with at least 10x, 20x and 30x sequencing read depth, respectively (S1B Fig). Approximately 3.8 million high-confidence autosomal variants were called per sample. Variant call concordance between replicate sample pairs (n = 3) was >99.9% for single nucleotide polymorphisms (SNPs) and was 98.9% for insertions and deletions (InDels; S1 Table).

**Table 1:** Demographic characteristics of sequenced APIC and URECA participants.

Characteristic	All	APIC	URECA
Number	1035	508	527
Age, years, mean (SD)	10.3 (2.5)	10.9 (3.1)	9.6 (1.1)
Female sex	477 (46%)	216 (43%)	261 (50%)
*Race/Ethnicity*			
Black (non-Hispanic)	696 (67%)	319 (63%)	377 (72%)
White (non-Hispanic)	14 (1%)	7 (1%)	7 (1%)
Hispanic	258 (25%)	153 (30%)	105 (20%)
Other/mixed	64 (6%)	26 (5%)	38 (7%)
Unknown	3 (<1%)	2 (<1%)	1 (<1%)
*Site*			
Baltimore	234 (23%)	85 (17%)	149 (28%)
Boston	189 (18%)	65 (13%)	124 (23%)
Chicago	62 (6%)	62 (12%)	-
Cincinnati	45 (4%)	45 (9%)	-
Dallas	38 (4%)	38 (9%)	-
Denver	59 (6%)	59 (12%)	-
Detroit	50 (5%)	50 (10%)	-
New York	164 (16%)	64 (13%)	100 (19%)
St. Louis	155 (15%)	-	155 (29%)
Washington, D.C.	39 (4%)	39 (8%)	-
Household income < $15k	550 (54%)	273 (54%)	277 (54%)
Caretaker completed HS	756 (73%)	364 (72%)	392 (74%)
Caretaker smokes*	315 (33%)	123 (27%)	192 (39%)
Asthma	681 (75%)	508 (100%)	173 (43%)
BMI, Z-score, mean (SD)	0.9 (1.2)	1.0 (1.2)	0.8 (1.1)
FEV_1_, % predicted, mean (SD)	94.9 (16.3)	91.9 (17.6)	98.5 (14.5)
FEV/FVC, mean (SD)	0.80 (0.09)	0.78 (0.10)	0.83 (0.07)

Results are presented as counts and percentages or as means with standard deviations. Missing data were not included in percentage calculations. Ages for URECA correspond to the year the genome-wide association study lung function data were collected.

*Caretaker smoking status in URECA was collected at age 10. APIC: Asthma Phenotypes in the Inner City; BMI: body mass index; FEV_1_: forced expiratory volume in one second; FEV_1_/FVC: ratio of FEV_1_ to forced vital capacity; HS: high school; URECA: Urban Environment and Childhood Asthma.

**Fig 1:**
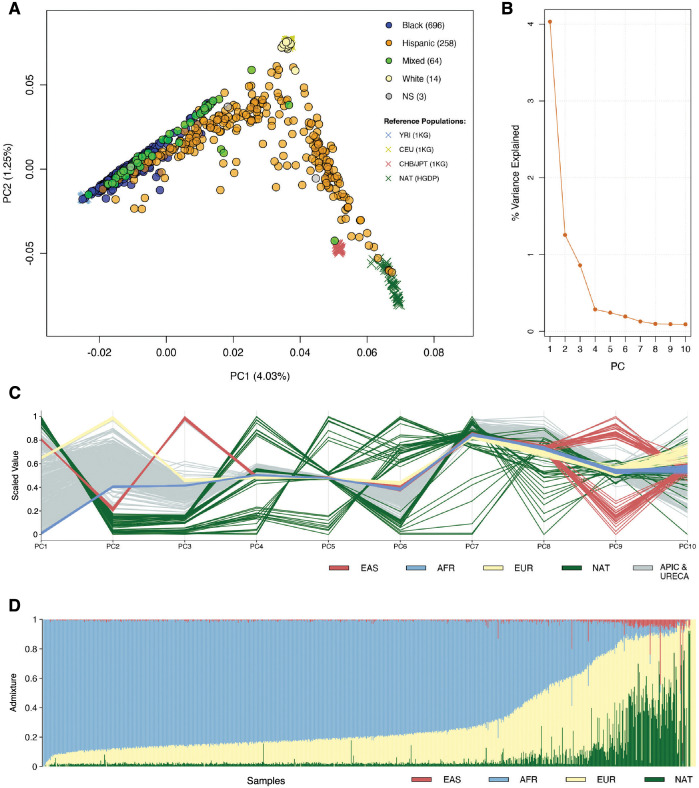
Ancestry composition of sequenced APIC & URECA participants. A) The top two principal components (PCs) of ancestry are plotted for sequenced APIC & URECA participants, colored by self-identified race/ethnicity, along with the four ancestry reference populations used for determining ancestry. NS = not specified. B) The proportion of genetic variance explained by each of the top 10 PCs. C) The relative values of the top 10 PCs are plotted for each sample, colored by reference population. D) The estimated proportion of admixture from each ancestral population is shown for each sequenced APIC & URECA participant. Each vertical line corresponds to one sample. 1KG, 1000 Genomes project; HGDP, Human Genome Diversity Project; YRI, Yoruba in Ibadan, Nigeria; CEU, Utah residents with Northern and Western European ancestry; CHB, Han Chinese in Beijing, China; JPT, Japanese in Tokyo, Japan; NAT, Native Americans from HGDP; EAS, East Asian ancestry; AFR, African ancestry; EUR, European ancestry.

The sequenced cohort included 696 (67%) participants who self-identified as non-Hispanic Black and 258 (25%) who self-identified as Hispanic (Table 1). Principal component and admixture analyses using genotypes were conducted to characterize the ancestry of the participants (Fig 1). This revealed that the genetic ancestry of our sample was 66% African, 26% European, 7% Native American, and 1% East Asian. The cohort was 54% male and included 681 (66%) children diagnosed with asthma (Table 1).

**Fig 2:**
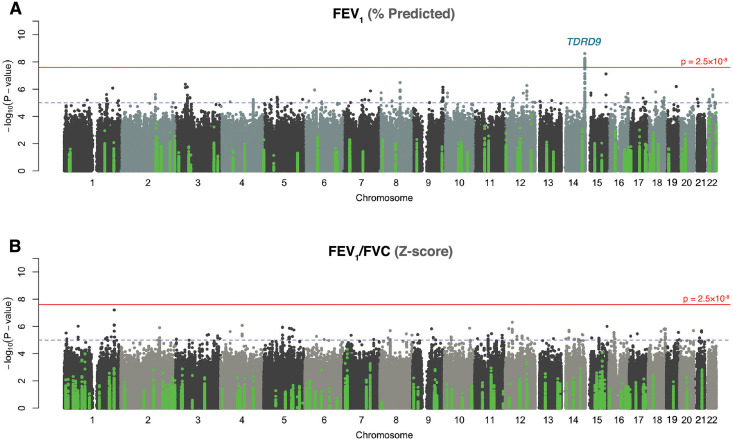
Genome-wide association results. GWAS Manhattan plots for **A)** FEV_1_ and **B)** FEV_1_/FVC ratio. The horizontal red line indicates genome-wide significance (p ≤ 2.5x10^−8^). The dotted horizontal blue line indicates p = 1x10^−5^. Variants colored in green are in previously identified GWAS loci [23]. FEV_1_, forced expiratory volume in one second; FEV_1_/FVC, ratio of FEV_1_ to forced vital capacity.

**Fig 3:**
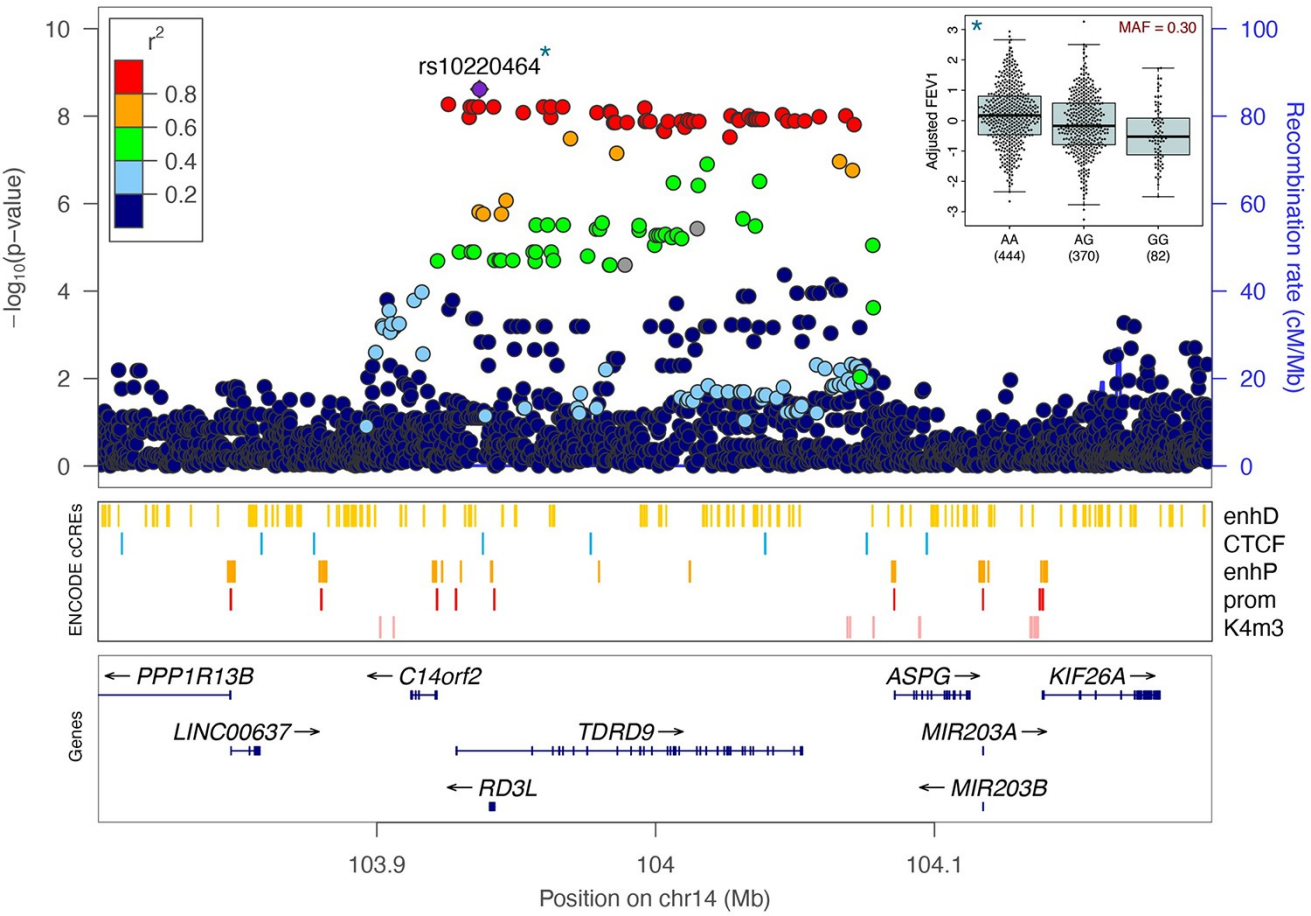
FEV_1_-associated variants on chromosome 14q32.33. FEV_1_ association results are shown at the *TDRD9* gene locus. Each variant is plotted according to its position and -log_10_ p-value, colored by linkage disequilibrium to the lead variant, rs10220464, within the sample. Candidate cis-Regulatory Elements (cCREs) from ENCODE [59] are also shown for the region. The inset panel in the upper right shows the distribution of adjusted FEV_1_ values by rs10220464 genotype. FEV_1_, forced expiratory volume in one second; MAF, minor allele frequency; EnhD, distal enhancer-like signature; CTCF, CCCTC-binding factor sites; enhP, proximal enhancer-like signature; prom, promoter-like signature; K4m3, trimethylation of histone H3 at lysine 4.

**Fig 4:**
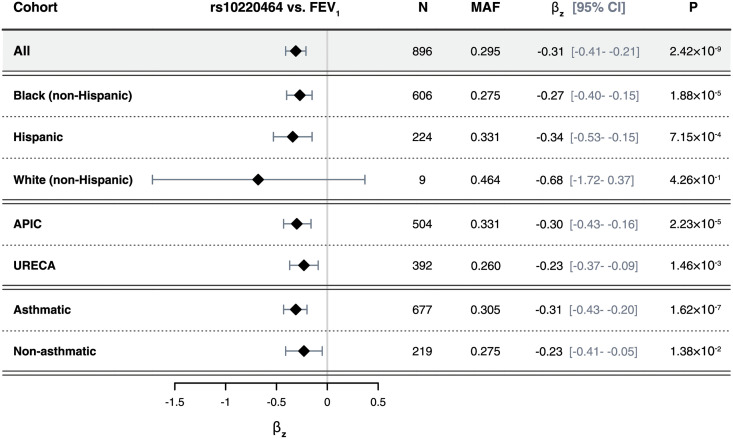
Rs10220464 effect size heterogeneity. A forest plot of the associations between rs10220464 and FEV_1_ (% predicted) are shown for distinct sub-cohorts distinguished by self-identified race/ethnicity, study, and asthma status. β_z_, the association effect size between the rs10220464 allele count and the adjusted and normalized FEV_1_ (% predicted) values; FEV_1_, forced expiratory volume in one second; N, total number of individuals included in the association test; MAF, minor allele frequency within the sub-cohort; P, the association p-value.

Using the WGS variant calls for 14.1 million variants with minor allele frequency (MAF) ≥0.01, we performed a GWAS of two lung function traits: FEV_1_ (% predicted) and FEV_1_/FVC (Z-scores), measured between ages 5–17 (Table 1, S2 Fig), adjusting for age, sex, asthma diagnosis, the first 10 principal components (PCs) of ancestry, and sample relatedness using a linear mixed model [58]. The FEV_1_ GWAS included 896 participants from APIC (n = 504) and URECA (n = 392), and the FEV_1_/FVC GWAS included 886 participants from APIC (n = 497) and URECA (n = 389). The genomic control factor, λ_GC_, for both GWAS results was 1.02 (S3 Fig), indicating adequate control for population stratification. We identified one locus on chromosome 14q32.33 that was associated with FEV_1_ at genome-wide significance (p<2.5x10^-8^); no other variants were associated with FEV_1_ and no variants were associated with FEV_1_/FVC at genome-wide levels of significance (Fig 2). The FEV_1_ locus on chromosome 14 consisted of a 200 kb region of associated variants in high linkage disequilibrium (LD) across the *TDRD9* (Tudor Domain Containing 9) gene (Fig 3, S2 Table). The minor allele at the lead SNP (rs10220464; MAF = 0.30) was significantly associated with lower FEV_1_ (p = 2.4x10^-9^; β_z_ = -0.31, 95% confidence interval (CI) = -0.41- -0.21) and nominally associated with lower FEV_1_/FVC (p = 1.1x10^-3^; β_z_ = -0.17, 95% CI = -0.28- -0.07). Fine-mapping analysis at this locus (chr14:103.7–104.3Mb) revealed one 95% credible set of effect variables consisting of 59 SNPs, with rs10220464 having the highest individual posterior inclusion probability among them (S4 Fig). We did not detect any significant differences in rs10220464 association effect size by ancestry or asthma status or study for FEV_1_ (Fig 4). Furthermore, the *TDRD9* locus remained the only genome-wide significant association when the two GWAS were performed without adjustment for asthma status (S5 Fig). The overall effect size correlations between asthma-adjusted and unadjusted GWAS results were r = 0.981 for FEV_1_ and r = 0.954 for FEV_1_/FVC.

We examined association results for the previously identified FEV_1_ and FEV_1_/FVC loci reported in the meta-analysis of the UK Biobank and SpiroMeta Consortium by Shrine and colleagues (n = 400,102) [23], which included 70 loci for FEV_1_ and 117 for FEV_1_/FVC. Of these, 64 of the lead SNPs for FEV_1_ and 112 for FEV_1_/FVC were genotyped in the APIC and URECA sample. Only one SNP, for FEV_1_, replicated with false discovery rate (FDR) q<0.05 (rs9610955; p = 1.0x10^-4^; β_z_ = -0.38, 95% CI = -0.58- -0.19; S6 and S7 Figs). Cumulatively, 56% (n = 36) and 54% (n = 60) of these SNPs demonstrated consistent directions of effect for FEV_1_ and FEV_1_/FVC, respectively, with effect size correlations of 0.29 (95% CI = 0.05–0.50; p = 0.020) for FEV_1_ and 0.42 (95% CI = 0.25–0.56; p = 4.2x10^-6^) for FEV_1_/FVC.

### Lung function risk alleles are associated with DNA methylation at the *TDRD9* locus in airway epithelial cells

**Fig 5:**
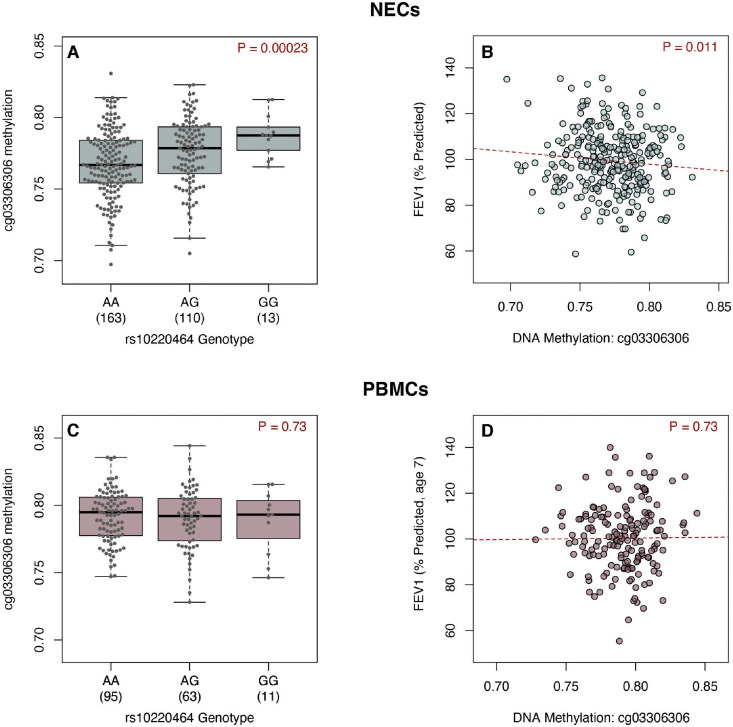
Genotype and FEV_1_ associations with DNA methylation. DNA methylation levels at cg03306306 are shown by rs10220464 genotype and FEV_1_ measures are plotted against cg03306306 methylation levels in NECs at age 11 (A, B), and PBMCs at age 7 (C, D) from URECA. FEV_1_, forced expiratory volume in one second; NECs, nasal epithelial cells; PBMCs, peripheral blood mononuclear cells; URECA, Urban Environment and Childhood Asthma study.

The majority of complex trait-associated variants exert effects by altering gene regulatory networks [60–62]. These changes are often marked by quantitative differences in DNA methylation levels [63–65]. We therefore investigated correlations between the FEV_1_-associated allele at *TDRD9* and DNA methylation at the locus in upper airway (nasal) epithelial cells (NECs) from URECA children at age 11 (n = 286). We tested for associations between the FEV_1_ genotype, as tagged by rs10220464, and DNA methylation levels at 796 CpG sites within 10 kb of any *TDRD9* locus variants associated with FEV_1_ at p<1x10^-5^ (n = 82 variants). The rs10220464 genotype was an meQTL for 5 CpG sites at an FDR <0.05 (S3 Table). DNA methylation levels at only one of these CpG sites, cg03306306 (p = 2.3 x10^-4^; β = 0.07, 95% CI = 0.03–0.10; Fig 5A), was also significantly associated with FEV_1_ at age 10 in URECA (p = 0.011; β = -11.48, 95% CI = -20.27- -2.69; Fig 5B). The rs10220464 genotype accounted for 4.7% of residual variation in cg03306306 methylation, and cg03306306 methylation explained 2.4% of residual variation in FEV_1_.

We then analyzed cg03306306 methylation in PBMCs collected at age 7 (n = 169) [66] from URECA children to evaluate whether the genotype and lung function associations observed in NECs were shared with blood cells. In PBMCs, we observed no correlation between the rs10220464 risk allele and cg03306306 methylation (Fig 5C), nor was there an association between cg03306306 methylation and FEV_1_ (Fig 5D). These results indicate that cg03306306 methylation dynamics in the airway epithelium are not present in peripheral blood cells.

### Smoking exposure is associated with DNA methylation at the *TDRD9* locus

**Fig 6:**
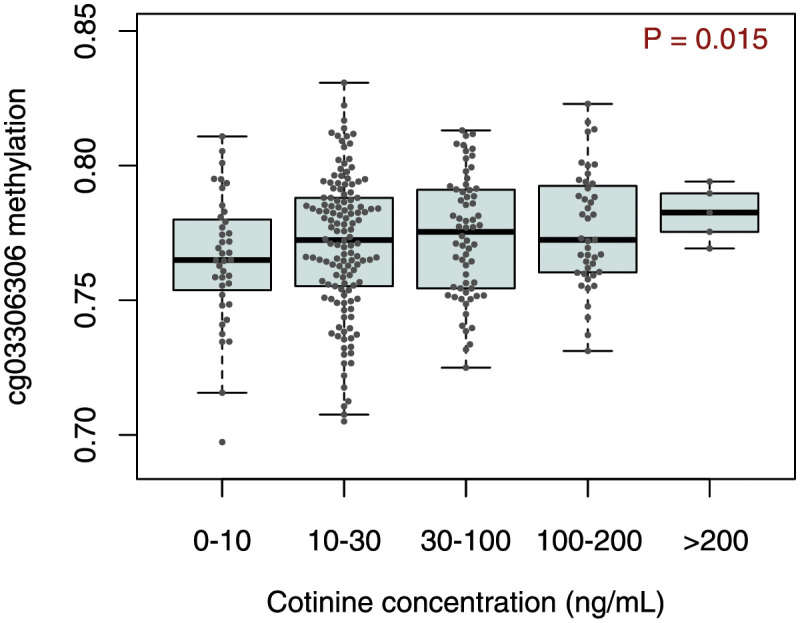
DNA methylation association with smoking exposure. DNA methylation at cg03306306 in nasal epithelial cells at age 11 are plotted against urine cotinine levels from URECA at ages 8–10 as measured using the NicAlert assay (n = 285). URECA, Urban Environment and Childhood Asthma study.

DNA methylation at the *TDRD9* locus had previously been associated with maternal smoking during pregnancy [67,68]. Therefore, we tested for associations between environmental tobacco smoke exposure (S8 Fig) and DNA methylation at this locus in the URECA children. Methylation at cg03306306 in NECs was significantly associated with nicotine metabolite (cotinine) levels in urine collected at ages 7–10 years (p = 0.015; β = 0.03, 95% CI = 0.01–0.05; Fig 6). Methylation at cg03306306 in PBMCs from age 7 was not associated with urine cotinine levels.

To determine if there was an interaction effect between genotype and smoking exposure on DNA methylation and/or lung function, we repeated the cotinine association tests in URECA with the addition of an interaction term to assess if the genotype effect differed between individuals with low and high exposures to smoking. There were no significant genotype-by-smoking exposure interaction effects on methylation levels in NECs or PBMCs in URECA, nor were there any significant methylation-by-smoking effects on FEV_1_ (S9 Fig). There was modest evidence for a genotype-by-smoking exposure interaction effect on FEV_1_ in the combined APIC and URECA sample, but this did not reach statistical significance (p = 0.06, S10 Fig). Considering the ages of the participants in APIC and URECA, most tobacco exposures were likely due to secondhand smoke.

### Genetic effects on lung function are mediated by DNA methylation

To determine if DNA methylation at the *TDRD9* locus had a causal effect on lung function, we performed a Mendelian randomization analysis using two-stage least squares (2SLS) regression. In the first stage, cg03306306 methylation levels in NECs were regressed on an instrument composed of four meQTLs for cg03306306 (rs11160777, rs137961671, rs7143936, rs11160776; Materials and methods). In the second stage, FEV_1_ was regressed on the predicted DNA methylation values generated from the first stage regression, thereby yielding a causal effect estimate of cg03306306 methylation on FEV_1_. Urine cotinine levels were included as a covariate in both stages. The variance explained in the first stage regression was r^2^ = 0.11. The causal effect of cg03306306 methylation on FEV_1_ was statistically significant (p = 0.020). We also tested a single, unweighted allele score of the instrumental variables and observed a causal effect association of p = 0.045 (stage-one r^2^ = 0.10). We further performed a bootstrapped mediation analysis to test whether the rs10220464 risk allele effect on FEV_1_ was mediated by DNA methylation. The indirect effect of rs10220464 on FEV_1_ via cg03306306 methylation was significant, both when including asthma as a covariate (β_z_ = -0.04, 95% CI = -0.10- -0.003, percent mediated = 14.4%) and when asthma was not considered (β_z_ = -0.04, 95% CI = -0.10- -0.002, percent mediated = 15.0%). These results indicate that the effect of the FEV_1_-associated genotype at the *TDRD9* locus is partially mediated through its impact on nearby DNA methylation levels.

### Gene expression and promoter-enhancer interactions implicate *PPP1R13B*

**Fig 7:**
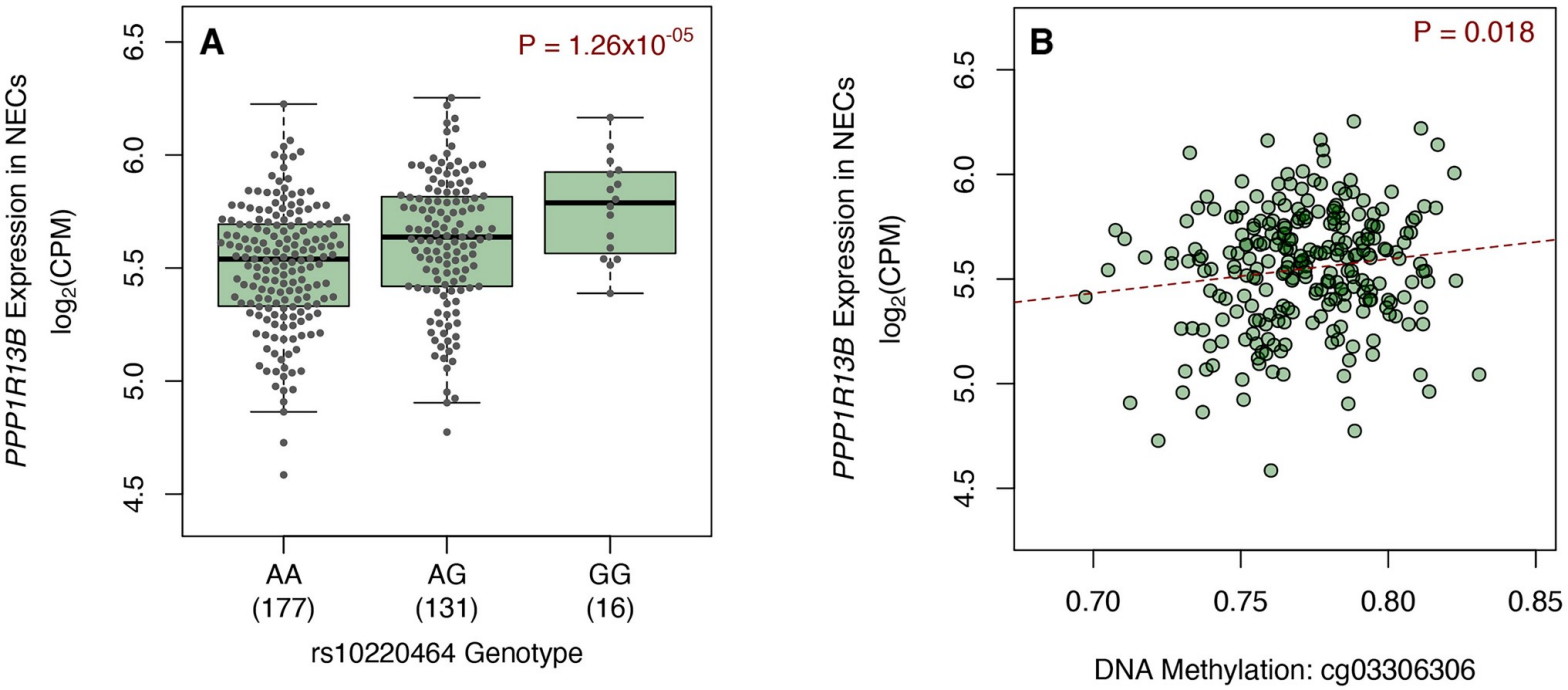
*PPP1R13B* gene expression in NECs. *PPP1R13B* gene expression in NECs at age 11 are plotted against A) rs10220464 genotype (n = 324) and B) DNA methylation at cg03306306 in NECs at age 11 (n = 254). NECs, nasal epithelial cells; CPM, counts per million.

Trait-associated variants and DNA methylation often affect the transcriptome by influencing the expression of one or more neighboring genes [69,70]. Identifying these correlations can help infer causal mechanisms [71]. Therefore, we next explored the relationship between the genotype for the lead FEV_1_ variant rs10220464 and the expression of genes within 1 Mb in NECs and PBMCs from URECA children. Notably, the rs10220464 genotype was not associated with *TDRD9* expression levels in these cells (NECs: p = 0.60, β = 0.12; PBMCs: p = 0.91, β = 0.014). Of the 27 genes that were evaluated (S4 Table), rs10220464 was significantly associated with the expression of only one gene, *PPP1R13B* (Protein Phosphatase 1 Regulatory Subunit 13B; FDR q = 2.77.x10^-4^; p = 1.3x10^-5^; β = 0.12, 95% CI = 0.06–0.17; Fig 7A), in NECs. *PPP1R13B* expression levels were also the most strongly associated of the 27 genes with methylation at cg03306306 in NECs (p = 0.018; β = 0.10, 95% CI = 0.02–0.18; Fig 7B). *PPP1R13B* expression in NECs, however, was not associated with FEV_1_ or smoking exposure (S11 Fig).

**Fig 8:**
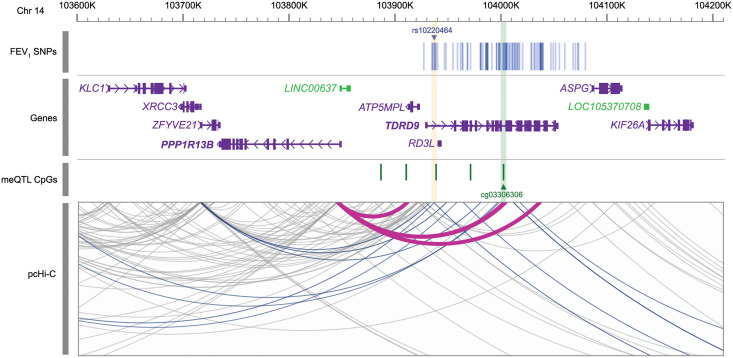
Promoter-enhancer interactions at *TDRD9* locus in nasal epithelial cells. Promoter-to-enhancer chromatin interactions captured by Hi-C in nasal epithelial cells from URECA at age 11 are displayed as grey arcs. SNPs associated with FEV_1_ (p<1x10^-5^) are marked by blue lines in the top row according to their genomic position on chromosome 14. The lead FEV_1_ SNP, rs1022464, is highlighted in yellow. CpG sites associated with rs1022464 (FDR<0.05) are displayed as green markers below the genes, with cg03306306 highlighted in green. Chromatin Interactions containing SNPs associated with FEV_1_ (p<1x10^-5^) are highlighted in blue. Magenta arcs highlight interactions between the *PPP1R13B* promoter and regions containing FEV_1_ SNPs and/or rs1022464-associated CpG sites. FEV_1_, forced expiratory volume in one second; SNPs, single nucleotide polymorphisms; meQTL, methylation quantitative trait locus; pcHi-C, promoter capture Hi-C.

The transcription start site of *PPP1R13B* resides 87 kb from rs10220464 and 152 kb from cg03306306, suggesting long-range interactions between the FEV_1_-associated genotype and the promoter of *PPP1R13B*. To determine whether any of the FEV_1_-associated GWAS variants at the *TDRD9* locus resided in regions that physically interacted with the promoters of *cis*-genes, we evaluated chromatin interactions in lower airway (bronchial) epithelial cells (BECs) [72], assessed by promoter-capture Hi-C. Forty-two of the GWAS variants resided in regions that interacted with the promoters of 9 different genes expressed in NECs (Fig 8; S5 Table). The gene most frequently mapped to these variants was *PPP1R13B*, with 15 variants located in 3 different interaction loops. Moreover, the strongest observed interaction was between a region containing 4 FEV_1_-associated variants and the *PPP1R13B* promoter (CHiCAGO score = 9.38; S5 Table), suggesting that this region is an enhancer for *PPP1R13B* expression. This putative enhancer region is located just 2.21 kb from cg03306306.

### Summary of study associations

**Fig 9:**
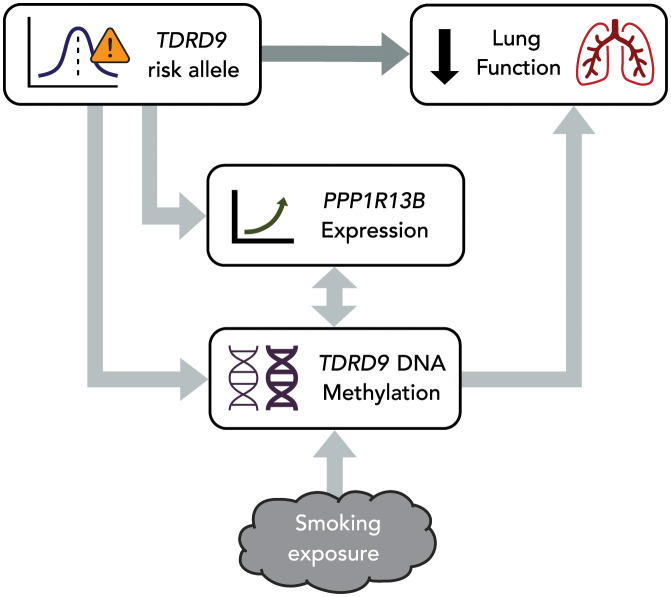
Summary of study associations. The *TDRD9* locus was significantly associated with FEV_1_ (% predicted) in the APIC and URECA cohorts. This association was partially mediated by DNA methylation at the cg03306306 CpG site in *TDRD9* in NECs, which was also significantly associated with environmental tobacco smoke exposure. The TDRD9 risk allele and DNA methylation were both significantly associated with *PPP1R13B* gene expression, but *PPP1R13B* gene expression was not significantly correlated with FEV_1_ itself. Unidirectional arrows represent inferred causality.

The associations between the *TDRD9* risk allele, cg03306306 DNA methylation in NECs, smoking exposure, *PPP1R13B* gene expression, and FEV_1_ (% predicted) reported in this study are summarized in Fig 9.

## Discussion

Using whole-genome sequence variant calls in an asthma-enriched cohort of predominantly African-American children raised in urban environments, we identified a genotype at the *TDRD9* locus associated with lower FEV_1_% predicted. This genotype effect was partially mediated by DNA methylation in airway epithelial cells, which were also correlated with smoking exposure. Data from RNA-sequencing and promoter-capture Hi-C in airway epithelial cells suggested that these FEV_1_-associated genetic and epigenetic variations influence the expression of the *PPP1R13B* gene through long-range interactions.

The *PPP1R13B* gene encodes a protein that promotes apoptosis, a form of programmed cell death, via its interaction with the tumor suppressor p53 and is often referred to by its alias ASPP1 (apoptosis-stimulating protein of p53 1) [73]. In response to oncogenic stress, PPP1R13B translocates to the nucleus, where it enhances the transcriptional activity of p53 on specific target genes relevant to apoptosis [74,75]. Exposure to smoking and fine particulate matter induces epithelial apoptosis in the lung via p53 [76–78]. PPP1R13B may also promote apoptosis in a p53-independent manner by inhibiting autophagy in response to upregulation by EGR-1 (early growth response protein 1) [79]. EGR-1 mediates stress-induced proinflammatory responses in the airway epithelium and contributes to the pathogenesis of COPD [80–85]. Within the lung, *PPP1R13B* is indeed predominantly expressed in epithelial cells, particularly in alveolar type 2 cells, and less so in immune cells and fibroblasts [86,87]. However, Cheng and colleagues studied PPP1R13B function in lung fibroblasts and found that it was upregulated following SiO_2_ exposure, where it promoted fibroblast proliferation and migration through endoplasmic reticulum stress and autophagy pathways [88]. Overall, these studies suggest that *PPP1R13B* plays a key role in maintaining tissue homeostasis by regulating apoptosis and autophagy in response to environmental stimuli [74,89,90]. The specific function(s) of this gene in the airway epithelium and its potential impact on the development of airway obstruction remain to be elucidated. *PPP1R13B* expression in airway epithelial cells at age 11 was not associated with lung function or urine cotinine levels in the URECA children, but the cofactors of this gene [79,91] have been found previously to be upregulated in smokers with COPD [81,92]. Given its association with lung function alleles in our study, its expression in the airway epithelium, and its purported functions in autophagy and apoptosis pathways, additional study of *PPP1R13B* in lung and airway development is warranted, particularly in the context of adverse environmental stimuli, many of which are enriched in low-income urban environments.

In NECs, *PPP1R13B* gene expression was significantly associated with DNA methylation levels at the cg03306306 CpG site in *TDRD9*. Methylation at the *TDRD9* locus was previously reported to correlate with specific environmental exposures [67,68,93] and with *TDRD9* expression in blood [67,94]. *TDRD9* is lowly expressed in the lung but is detected in alveolar macrophages and in monocytes [86,87]. Interestingly, the gene was among the most differentially expressed genes in alveolar macrophages in smokers relative to non-smokers [95], and its knockdown in TDRD9*-*expressing lung carcinomas resulted in increased apoptosis [96]. Its expression was not correlated with the rs10220464 genotype in URECA NECs or PBMCs, but rs10220464 is an eQTL for *TDRD9* expression in whole blood in GTEx data [97], with the minor allele associated with lower *TDRD9* expression. Although evidence from this study points to *PPP1R13B* in the airway epithelium, we can’t exclude the possibility that *TDRD9* or other genes could contribute to the locus’ influence on lung function via other tissues.

The FEV_1_ association signal at the *TDRD9* locus included many variants in high LD across a 200 kb region that could be independently contributing to function. Some of the variants lie in different long-range enhancers [59]. It is also possible that one or more correlated variants were not included because they failed quality control standards. In addition, due to the limited sample size of the WGS cohort, we excluded rare variants (MAF<0.01) from consideration, which could contribute to the signal at this locus. Additional functional studies are needed to identify the causal variant(s) and full mechanism of action.

The correlations of rs10220464, FEV_1_, and smoking exposure with cg03306306 methylation in NECs were absent in PBMCs. Although global DNA methylation patterns between tissues are highly correlated [98], tissue-specific differentially methylated regions are more likely to be functional, particularly if they are positively correlated with gene expression [99]. The *TDRD9* locus has not been identified in epigenome-wide association studies of lung function [44,100–104], but these measured DNA methylation from blood, which may be an insufficient proxy for methylation in the lungs [105]. Indeed, previous studies have found that DNA methylation profiles in NECs are significantly more predictive of pediatric asthma than those in PBMCs [106,107]. Furthermore, epigenetic biomarkers can change with age. For example, epigenetic markers for lung function in adults do not replicate in children [101].

We tested for interactions between smoking exposure and rs10220464 genotype effects on cg03306306 and on FEV_1_ and between smoking exposure and cg03306306 methylation effects on FEV_1_. We did not detect any significant interactions, but our analyses in that regard could have been underpowered given our observed effects and sample sizes [36]. Furthermore, because this study was limited to children living in low-income urban neighborhoods, environmental risk factors are likely to be more prevalent than in the general population [55–57]. Additionally, such exposures are not necessarily ubiquitous across all the different neighborhoods and communities represented in this sample, and although environmental tobacco smoke exposure was examined and the socioeconomic range represented in this study is relatively narrow, there could be relevant environmental factors that were not considered.

To infer causality, Mendelian randomization and mediation analyses rely on assumptions that are often difficult to empirically verify. For the Mendelian randomization analysis, we identified instrumental variants associated with the intermediate cg03306306 that were not independently associated with the outcome, FEV_1_. However, because these variants were selected from the same dataset that the outcome testing was performed in, they were susceptible to bias from winner’s curse [108]. To mitigate the potential impact from this effect and from weak instruments, we performed a secondary analysis in which we combined the instrumental variants into a single, unweighted score. For the mediation analysis, unmeasured confounding can invalidate direct and indirect effect estimates [109]. To protect against such bias, we systematically tested for confounding associations with additional environmental measures available in APIC and URECA (Materials and methods). Nonetheless, there may still exist unknown confounding factors that were not measured. Ultimately the results of the Mendelian randomization and mediation analyses indicate that methylation at cg03306306 in NECs mediated the rs10220464 genotype effect on FEV_1_, but there was residual correlation between rs10220464 and FEV_1_, signifying that the genotype effect was only partially mediated by cg03306306.

Another limitation of our study was the relatively small size for a GWAS. This likely contributed to the lack of statistically significant replication for previously identified lung function loci [23], considering that the observed effects were correlated with results of prior GWAS. However, the APIC and URECA cohorts represent understudied, high-risk, pediatric populations that likely harbor distinct genetic and environmental risk factors compared to older, primarily European ancestry cohorts included in previous GWAS of lung function [14–20,23]. The findings of this study have yet to be replicated in an independent cohort, and should therefore be considered preliminary; however, it is possible that these associations would differ in populations with dissimilar ancestry, age, exposures, and/or asthma risk.

There are additional caveats to consider when interpreting our findings. First, this study integrated data from two cohorts with different recruitment criteria, asthma definitions, and ancestral compositions. Furthermore, most of the analyses beyond the GWAS were limited to subsets of the URECA participants. However, we did not observe significant genetic effect heterogeneity for rs10220464 by study, asthma status, or ancestry. To control for potential population stratification, we used the first ten PCs of ancestry to adjust lung function values and then included the ancestry PCs as fixed effects in the GWAS models (Materials and methods). The linear mixed models also included a genetic relatedness matrix as a random effect to account for residual population structure. Because children with asthma have lower lung function overall (Table 1) and their lung function may be more affected by environmental exposures [110–112], we adjusted for asthma status in the GWAS, as in previous GWAS [113–116]. The likelihood of discovering lung function variants with consistent effects in asthmatics and non-asthmatics was thereby increased, although genetic determinants of lung function may differ by asthma status [117]. Furthermore, adjusting for disease status could potentially introduce collider bias [118]. The significant genotype effect at the *TDRD9* locus, however, remained the only genome-wide-significant association when asthma was excluded as a covariate, and adjustment for asthma did not substantively alter the mediation results. Second, some of the analyses used data collected at different timepoints. For example, most of the urine cotinine and spirometry measures were collected at age 10, but the samples used for the NEC DNA methylation and RNA-seq analyses were collected at age 11. Because DNA methylation and gene expression can change over time [40,119–121], their values at age 11 may not be fully representative of exposures at age 10. Finally, the promoter-capture Hi-C data were from lower airway (bronchial) epithelial cells, whereas the DNA methylation and RNA-seq data were generated from upper airway (nasal) epithelial cells. Although there are transcriptomic differences between epithelial cells from each compartment, their respective profiles are highly correlated [122–126], and the use of NECs as a proxy for the lower airway epithelium has been validated for both gene expression and epigenetic studies [124–127].

Our study identified a novel avenue through which genetic risk and environmental exposures could affect the airways of children raised in low-income urban neighborhoods. Further research into this pathway may yield mechanistic insights into the early development of impaired lung function, perhaps leading to interventions that can help reduce the high incidence and morbidity of chronic respiratory diseases in socioeconomically disadvantaged children.

## Materials and methods

### Ethics statement

The institutional review boards (IRBs) from all participating sites of the URECA (ClinicalTrials.gov Identifier: NCT00114881) and APIC (ClinicalTrials.gov Identifier: NCT01383941) studies gave initial ethical approval for this work. These include IRBs from the following institutions: National Jewish Health, Denver, CO (APIC); Children’s National Medical Center, Washington, DC (APIC); Children’s Memorial Hospital, Chicago, IL (APIC); Johns Hopkins University, Baltimore, MD (APIC & URECA); Boston University School of Medicine, Boston, MA (APIC); Henry Ford Health Center, Detroit, MI (APIC); Columbia University Medical Center, New York, NY (APIC & URECA); Cincinnati Children’s Hospital, Cincinnati, OH (APIC); University of Texas Southwestern Medical School, Dallas, TX (APIC); Boston Medical Center, Boston, MA (URECA); Saint Louis Children’s Hospital, Saint Louis, MO (URECA). In 2014, ethical oversight for these studies transitioned to a single, central IRB managed by WGC IRB (formerly Western IRB), whereupon WGC IRB gave ethical approval for this work [128]. Written informed consent was obtained from legal guardians of all participating children, who also assented.

### Study population and phenotypes

We analyzed samples and phenotypes from two National Institutes of Allergy and Infectious Diseases (NIAID)-funded asthma studies conducted by the Inner-City Asthma Consortium (ICAC) [129]: the Asthma Phenotypes in the Inner City (APIC) study [55,56] and the Urban Environment and Childhood Asthma (URECA) birth cohort study [57]. The APIC study was a 1-year, prospective, epidemiological investigation of children and adolescents with asthma (ages 6–17) living in low-income areas (≥20% of residents below poverty level) in nine U.S. cities (Baltimore, MD; Boston, MA; Chicago, IL; Cincinnati, OH; Dallas, TX; Denver, CO; Detroit, MI; New York, NY; Washington, DC). The APIC participants were required to have a diagnosis of asthma by a physician and to have had at least two episodes requiring bronchodilator administration within the past year [55]. The URECA study enrolled pregnant women living in low-income areas of four U.S. cities (Baltimore, MD; Boston, MA; New York, NY; St. Louis, MO) who reported that either or both parents of the index pregnancy had a history of asthma or allergic diseases [57]. This prospective, longitudinal study followed each child through adolescence, periodically collecting samples and clinical and environmental exposure data.

Lung function was assessed using spirometry. Lung function measures used in this study for APIC participants were taken at the study entry visit (V0). For URECA, measurements from age 10 were used when available; otherwise, the most recent measurement after age 5 was used (S6 Table). Asthma status was assigned according to study-specific criteria. For APIC, asthma was defined by a doctor’s diagnosis of asthma and short-acting beta-agonist use in the year prior [55]. For URECA, asthma status was determined either by doctor diagnosis, lung function reversibility, or symptom recurrence [130]. The 2012 Global Lung Initiative reference equations [131] were applied to generate percent predicted estimates for FEV_1_ and Z-scores for FEV_1_/FVC ratio. Urine cotinine levels were measured using NicAlert immunochromatographic assays, which report results on a scale of 0–6 according to different cotinine concentration ranges [132]. For URECA, urine cotinine results were available at age 10 for most participants (n = 391); otherwise, assays from age 8 (n = 29) or age 7 (n = 2) were used. This study utilized DNA methylation and RNA-seq data generated for other URECA studies; therefore, the number of samples included in each analysis varied and was limited by data availability (S7 Table, S12 Fig).

### Whole-genome sequencing and data processing

DNA was extracted from peripheral blood (APIC, URECA) or cord blood (URECA) and quantified using an Invitrogen Qubit 3 Fluorometer. DNA quality was assessed using the Thermo Scientific NanoDrop One spectrophotometer and confirmed using an Agilent TapeStation system. DNA was processed in batches of 60 using the Illumina Nextera DNA Flex library prep kit with unique dual adaptors. Each set of 60 libraries was sequenced over two NovaSEQ S4 flowcells. Whole-genome sequencing was performed by the University of Chicago Genomics Facility using the Illumina NovaSEQ6000, which generated 150 bp paired-end reads. Sequencing data processing followed the Broad Institute’s Genome Analysis Toolkit (GATK) best practices for germline short variant discovery, as implemented in the harmonized pipeline used by the New York Genome Center for TOPMed [133,134]. Reads were aligned to the GRCh38 human reference genome (including alternate loci and decoy contigs) using BWA-MEM (Burrows-Wheeler Aligner; v0.7.17). Aligned reads further underwent duplicate removal (Picard MarkDuplicates; v2.8.1) and base quality score recalibration (GATK BaseRecalibrator; v3.8) against known sites (dbSNP138, known InDels, and Mills and 1KG gold standard InDels) provided in the GATK resource bundle. Read alignment metrics were calculated using Picard CollectWgsMetrics (v2.8.1) for all aligned reads and for aligned reads with base quality and mapping quality ≥ 20. DNA contamination levels were estimated using VerifyBamID2 (v1.0.6) [135]. Samples with estimated DNA contamination >0.05 were removed from consideration. Samples with poor coverage (<50% of the genome with ≥20x depth) were also removed from further consideration. To identify potential sample swaps, WGS samples were validated using independent genotyping arrays.

### QC array for sample validation

To identify potential WGS sample swaps, we independently genotyped the APIC and URECA participants using the Illumina QC Array-24 BeadChip. SNPs were tested for Hardy-Weinberg Equilibrium (HWE) within each self-identified ancestry group using the chi-square test and removed if they deviated from HWE (Bonferroni-adjusted p<0.05) within at least one ancestry. SNPs with call rates <0.98 were also removed. Samples with total variant call rates <0.95 were not used. Array data with incorrect or indeterminate sex according to X-chromosome heterozygosity rates (Plink v1.90) were also not used [136]. For fourteen of the sequenced URECA samples, we used results from the Illumina Infinium CoreExome+Custom array for sample validation, which were generated and controlled for quality as described by McKennan and colleagues [137]. WGS and array genotypes were tested for concordance using VerifyBamID (v1.1.3) [138]. WGS samples that were not validated with array data were not included in genetic analyses (n = 2).

### Variant calling and quality control

Variant calls were generated using GATK HaplotypeCaller (v4.1.3.0), accounting for contamination estimates, for single nucleotide variants and short insertions, deletions, and substitutions. Sample genotypes were joined using GATK GenomicsDBImport and GenotypeGVCFs over the genomic intervals defined in the GATK WGS calling region interval list provided in the GATK resource bundle. Genotypes with read depth (DP) <10 or quality scores (GQ) <20 were set as missing. Sites with ≥0.1 missingness were then removed from consideration. Variants with minor allele frequencies >0.05 were tested for accordance with HWE, accounting for population structure [139]. Sites with common variants that deviated from structural HWE (P<1x10^-6^) were removed from consideration. Sites with quality by depth ratios (QD) <4 or >34 were also removed, as we observed declines in variant transition/transversion (TS/TV) ratios beyond these bounds (S13 Fig). Variant site quality was further evaluated using machine-learning-based Variant Quality Score Recalibration (VQSR). First, SNPs were modeled using GATK VariantRecalibrator (v4.1.3.0) with Hapmap 3 and with Omni 2.5M SNP chip array as truth resources, 1000G as a training resource, and dbSNP138 as a known sites resource. InDels were likewise trained with the Mills and 1KG gold standard InDels dataset as a truth resource and dbSNP138 as a known sites resource. SNPs and InDels with resultant predicted true positive probabilities below 0.997 and 0.990, respectively, were removed from consideration. Variant call accuracy was assessed by comparing call concordance between three replicate sequencing samples using VCFtools (v0.1.14) vcf-compare [140]. Variant call file manipulation was conducted using BCFtools (v1.10.2) [141].

### Ancestry estimation

Ancestry principal components (PCs) were calculated on the intersect of high quality single-nucleotide variants (SNVs) genotyped in the WGS data and several reference panels from the 1000 Genomes Project (1KG; n = 156) [142] and the Human Genome Diversity Project (HGDP; n = 52) [143]. Native American reference samples consisted of 52 samples from the HGDP with <5% non-native ancestry, according to an analysis of roughly 2 million markers using the program ADMIXTURE (v1.3.0) [144]. These samples were filtered for site quality (missingness 5%; ExcHet<60; VQSLOD≥8.3929), genotype quality (GQ≥20) and depth (DP≥10), MAF >0.02, and HWE (p>0.001) [143]. European, West African, and East Asian reference samples were randomly selected from CEU (n = 52), YRI (n = 52), JPT (n = 26), and CHB (n = 26) samples in the phase 3 1KG reference panel [142]. The combined genotypes were pruned for linkage disequilibrium (LD) ≤0.1 within 1Mb intervals. Ancestry PCs were calculated, accounting for subject relatedness, using PC-Air [145] and PC-Relate [146]. Initial kinship estimates were produced using KING [147]. Kinship and PCs were iteratively estimated using PC-Relate and PC-Air, respectively, until estimates for the top 5 PCs stabilized (n = 3). Reference population admixture estimates were estimated for each WGS sample with ADMIXTURE (v1.3.0), using the 1KG and HGDP reference samples for supervised analysis [144]. Because sample relatedness can lead to biased admixture estimates [145,148], admixture was estimated for each WGS sample separately.

### Quantitative trait association testing

Quantitative traits were adjusted for covariates and normalized using a two-stage approach [149,150]. First, each trait was regressed on age, sex, asthma status, and the first 10 PCs of ancestry. The residuals were then rank-normalized using an inverse normal transformation. In the second stage, the normalized residuals were considered outcome variables in the GWAS, adjusting for the same covariates as in the first stage. Genome-wide association testing was performed for all high-quality common variant calls (MAF≥0.01) using a linear mixed model, as implemented in GEMMA [58], with subject relatedness included as a random effect. Individuals who were not evaluated for asthma at ages 7 or 10 (n = 127) were excluded from trait association testing. The threshold we applied for genome-wide significance was P≤2.5x10^-8^, based on a 5x10^-8^ GWAS threshold and further accounting for two tests. To identify potential collider bias introduced by adjusting for asthma status, we repeated the GWAS without accounting for asthma status in either covariate-adjustment stage.

Fine-mapping analysis was conducted using SuSiE (SusieR R package v0.12.27) [151]. SuSiE applies a form of Bayesian variable selection in regression using iterative Bayesian stepwise selection to identify “credible sets” of variables. Each credible set has a 95% probability of containing at least one causal effect SNP. Prior to running SuSiE, we regressed asthma, age, sex, and ancestry PCs 1–10 from the genotype matrix and outcome vector (the normalized FEV_1_ residuals).

To explore whether there was lead-SNP effect heterogeneity by ancestry, study, or asthma status, we performed additional single-SNP quantitative trait association tests within several different sub-cohorts and introduced interaction effects into our models. For ancestry, we performed separate association tests in each of the non-Hispanic Black, Hispanic, and white populations, according to self-identified race/ethnicity. We then tested for genotype-by-ancestry interaction effects across APIC and URECA by using admixture proportions as covariates in our models, in lieu of ancestry PCs, and including an interaction term with the lead SNP for each continental ancestry group in turn. We tested these interaction effects using the—gxe argument in GEMMA in four separate models (one for each ancestry). To determine whether there was effect heterogeneity by study (APIC vs. URECA), we performed separate association tests in each study and also tested the association across APIC and URECA with the addition of a study covariate and a genotype-by-study interaction term. For asthma status, we performed separate association tests in the asthmatics and non-asthmatics and tested a genotype interaction term with asthma status.

### DNA methylation analysis

DNA from NECs was collected at age 11 from 287 URECA participants and assessed for genome-wide methylation patterns using the Illumina Infinium Human Methylation EPIC Beadchip. DNA methylation levels from PBMCs at age 7 in URECA were collected and processed as previously described [66]. MeQTL analysis was performed using Matrix eQTL [152]. NEC DNA methylation levels were adjusted globally for sex, array, plate, collection site, DNA concentration, percent ciliated epithelial cells, percent squamous cells, and ancestry PCs 1–3. Principal components analysis was then performed on the residual methylation levels, and the first three PCs were included as covariates in the meQTL association tests. Additional methylation PCs were not included in association tests, as they were significantly correlated with asthma phenotypes. Associations with FDR-adjusted P<0.05 were considered significant. MeQTL analysis with the PBMC data included sex, collection site, plate, ancestry PCs 1–3, and eight latent factors [153] (protecting for FEV_1_ at age 7) as covariates.

To test CpG site methylation associations with lung function in NECs, we performed linear regressions on the most recent FEV_1_ measures, with age, sex, ancestry PCs 1–3, and methylation PCs 1–3 as covariates. For the PBMC analysis, we set FEV_1_ at age 7 as the dependent variable, with sex, collection site, plate, ancestry PCs 1–3, and latent factors included as covariates.

For association testing with smoking exposures, we ran linear regressions for DNA methylation and lung function in NECs and PBMCs, as described above, with the addition of cotinine concentrations as a predictor. We further tested for smoking-by-genotype interaction effects on DNA methylation and lung function using these models by adding an interaction term (cotinine concentration: rs10220464 genotype). Proportions of explained variance were calculated by squaring partial correlation coefficients of regression model predictors [154]. One sample from one sibling pair was removed from all methylation analyses to prevent confounding due to relatedness.

### Mendelian randomization and mediation analysis

To assess the causal effects of DNA methylation on lung function, we performed one-sample Mendelian randomization analysis. We applied a 2SLS regression to URECA samples with WGS and DNA methylation data (n = 285) using ivreg [155]. DNA methylation levels in NECs at the cg03306306 CpG site were first adjusted for methylation PCs 1–3 and used as the endogenous, exposure variable. The adjusted and normalized FEV_1_ values from the GWAS were set as the dependent outcome variable. Urine cotinine levels were included as an exogenous covariate (included in both stages). The instrumental variables were chosen from a set of candidate SNPs that were at least nominally associated with cg03306306 methylation with p<0.15. Clustering of pairwise linkage disequilibrium values between these SNPs revealed six distinct haplotypes (S14 Fig). To ensure instrument exogeneity, each candidate SNP was tested for association with FEV_1_ after conditioning on cg03306306 methylation and urine cotinine, and SNPs associated with p<0.05 were removed from consideration. Of the remaining candidate SNPs, one was chosen from each haplotype, resulting in an instrument composed of 4 SNPs (rs11160777, rs137961671, rs7143936, rs11160776). Instrument relevance was validated using the F test, endogeneity using the Wu-Hausman test, and instrument exogeneity using the Sargon test. We tested two 2SLS models: one where the instrumental variables were included as individual predictors, and another featuring an unweighted allele score of the four instrumental variants to reduce potential bias from weak instruments and/or winner’s curse [156,157].

Mediation analysis was conducted with ROBMED [158]. The adjusted and normalized FEV_1_ residuals were set as the dependent variable, adjusted cg03306306 methylation as the mediator, and rs10220464 as the independent variable. Age at FEV_1_ measurement, sex, asthma status, ancestry PCs 1–3, and urine cotinine levels were included as covariates. We also performed a secondary mediation analysis without adjusting for asthma status. To identify additional, potential confounders that could invalidate our mediation model, we systematically tested for associations with 2 socioeconomic variables and 11 environmental exposures available in APIC and URECA (S8 Table, S15 Fig). For each environmental exposure, we tested whether the variable was associated with the mediator (cg03306306) and whether the variable was associated with the outcome (FEV_1_) conditional on the mediator. To ensure no exposure-mediator interactions, we repeated the cg03306306 association test with FEV_1_ with rs10220464 included as a predictor with a rs10220464: cg03306306 interaction term. The indirect effect of rs10220464 on FEV_1_ via cg03306306 methylation was estimated using 100,000 bootstrap resamples.

### Gene expression analysis

We analyzed gene expression in NECs and PBMCs from the URECA birth cohort using RNA-seq. The NEC data were derived from 323 children (155 females, 168 males) at age 11 years at the time of sample collection, and the PBMC data were derived from 130 (53 females, 77 males) PBMC children aged 2 years at the time of collection. Sequencing reads were mapped and quantified using STAR (v2.6.1) [159] and samples underwent trimmed means of M-value (TMM) normalization and voom transformation [160]. Genes with <1 count per million mapped reads (CPM) were removed from analysis. For eQTL association testing in NECs we corrected for sex, the first three ancestry PCs, collection site, epithelial cell proportion, sequencing batch, and 14 latent factors [153] using limma [161]. In PBMCs, we corrected for sex, the first three ancestry PCs, collection site, and 19 latent factors.

### Chromatin interaction analysis

Chromatin interactions were assessed using promoter capture Hi-C [162,163] in ex vivo human BECs from 8 adult lung donors, including 4 with asthma. The data were processed and analyzed as previously described [72,164]. Chromosomal interactions were evaluated using the CHiCAGO algorithm [165]. Interactions with CHiCAGO scores ≥5 were considered significant [165]. Genetic variants within 1 kb of a given interacting fragment were considered part of the chromatin loop. Genes that were not expressed in NECs were not included in the analysis.

## Supporting information

S1 Fig Whole-genome sequencing depth and coverage. **A)** Histogram of 1,035 whole-genome sequencing (WGS) samples from APIC and URECA by mean depth of coverage. **B)** Histogram of WGS samples based on proportion of genome covered at 20x, 25x, and 30x depth. APIC, Asthma Phenotypes in the Inner City study; URECA, Urban Environment and Childhood Asthma study.
(PDF)

S2 Fig Distribution of lung function measures by study. **A)** Distribution of FEV_1_ (% predicted) in APIC and URECA. **B)** Distribution of FEV_1_/FVC in APIC and URECA. APIC, Asthma Phenotypes in the Inner City study; URECA, Urban Environment and Childhood Asthma study. FEV_1_, forced expiratory volume in one second; FVC, forced vital capacity.
(PDF)

S3 Fig P-value distributions of GWAS results. Quantile-quantile plots of the GWAS results with corresponding genomic control factors (lambda) are shown for A) FEV_1_ (% predicted) and B) FEV_1_/FVC. FEV_1_, forced expiratory volume in one second; FVC, forced vital capacity.
(PDF)

S4 Fig Fine-mapping results for FEV_1_ (% predicted) at the *TDRD9* locus. The X-axis shows the chromosome position on chromosome 14. The Y-axis is the posterior inclusion probability (PIP). Variants highlighted in red represent a credible set, in which there is a 95% probability that at least one of the variants is causal. FEV_1_, forced expiratory volume in one second.
(PDF)

S5 Fig Genome-wide association results without adjustment for asthma. GWAS Manhattan plots for **A**) FEV_1_ and **B**) FEV_1_/FVC ratio, without adjustment for asthma status. The horizontal red line indicates genome-wide significance (p ≤ 2.5x10^−8^). The dotted horizontal blue line indicates p = 1x10^−5^. Variants colored in grey are the GWAS results with asthma adjustment. FEV_1_, forced expiratory volume in one second; FEV_1_/FVC, ratio of FEV_1_ to forced vital capacity.
(PDF)

S6 Fig Replication of FEV_1_ GWAS SNPs. Association statistics for previously identified FEV_1_ GWAS SNPs [23]. 64 out of 70 previously identified SNPs were genotyped in APIC & URECA. GWAS, genome-wide association study; SNP, single nucleotide polymorphism; APIC, Asthma Phenotypes in the Inner City study; URECA, Urban Environment and Childhood Asthma study. FEV_1_, forced expiratory volume in one second.
(PDF)

S7 Fig Replication of FEV_1_/FVC GWAS SNPs. Association statistics for previously identified FEV_1_/FVC GWAS SNPs [23]. 112 out of 117 previously identified SNPs were genotyped in APIC & URECA. GWAS, genome-wide association study; SNP, single nucleotide polymorphism; APIC, Asthma Phenotypes in the Inner City study; URECA, Urban Environment and Childhood Asthma study. FEV_1_, forced expiratory volume in one second; FVC, forced vital capacity.
(PDF)

S8 Fig NicAlert Results by Study. Distribution of urine cotinine levels, as measured using NicAlert immunochromatographic assays, which report results on a scale of 0–6 according to the labeled concentration ranges. Proportions were calculated relative to the number of samples with available NicAlert results. APIC, Asthma Phenotypes in the Inner City study; URECA, Urban Environment and Childhood Asthma study.
(PDF)

S9 Fig DNA methylation at cg03306306 by smoking exposure. DNA methylation levels at cg03306306 are shown by rs10220464 genotype in URECA participants with low and high smoking exposures in (**A**) NECs at age 11 and (**B**) PBMCs at age 7. FEV_1_ (% predicted) are also shown by cg03306306 DNA methylation levels in URECA participants with low and high smoking exposures in (**C**) NECs at age 11 and (**D**) PBMCs at age 7. NECs, nasal epithelial cells; PBMCs, peripheral blood mononuclear cells; FEV_1_, forced expiratory volume in one second; URECA, Urban Environment and Childhood Asthma study.
(PDF)

S10 Fig Genotype associations with FEV_1_ by smoking exposure. FEV_1_ (% predicted) are shown by rs10220464 genotype in APIC & URECA participants with low and high smoking exposures according to urine cotinine levels. FEV_1_, forced expiratory volume in one second; APIC, Asthma Phenotypes in the Inner City study; URECA, Urban Environment and Childhood Asthma study.
(PDF)

S11 Fig *PPP1R13B* expression in NECs vs. smoking exposure, FEV_1_. *PPP1R13B* expression in NECs at age 11 was not associated with smoking exposure at age 10 (**A**) nor with FEV_1_ (% predicted) at age 10 (**B**) in URECA. NECs, nasal epithelial cells; FEV_1_, forced expiratory volume in one second; URECA, Urban Environment and Childhood Asthma.
(PDF)

S12 Fig Data availability across APIC and URECA. Data availability for measures used in this study are shown for all sequenced samples. Each row represents a pattern of available and missing data, with green squares indicating available data and grey squares indicating missing data. Total counts of available data points for each variable are listed across the top of the figure. Total counts for each data availability pattern are listed along the right.
(PDF)

S13 Fig Transitions/transversions vs. quality/depth in WGS variant calls. The transition/transversion ratio (TS/TV) is plotted against the variant call quality/depth metric (QD) across all WGS SNP calls in APIC & URECA. Sites with QD less than 4 or greater than 34 were removed from consideration in this study. SNPs, single nucleotide polymorphisms; WGS, whole-genome sequencing; APIC, Asthma Phenotypes in the Inner City study; URECA, Urban Environment and Childhood Asthma study.
(PDF)

S14 Fig Intercorrelation of Mendelian randomization candidate instrument SNPs in URECA. Instrumental variables were chosen from a set of candidate SNPs that were at least nominally associated with cg03306306 methylation with p<0.15. The correlation values between these SNPs are shown, clustered using Ward’s method. The four SNPs used for the instrument are highlighted. URECA, Urban Environment and Childhood Asthma.
(PDF)

S15 Fig Intercorrelation of phenotypes and environmental variables in APIC & URECA. The correlations are shown between FEV_1_ (% predicted), smoking exposure (NicAlert), the primary the lead FEV_1_ SNP rs10220464, DNA methylation at cg03306306, 11 environmental exposures, and 2 socioeconomic indicators, clustered using Ward’s method. APIC, Asthma Phenotypes in the Inner City study; exp., exposure; URECA, Urban Environment and Childhood Asthma.
(PDF)

S1 Table Post-QC sequencing call concordance between replicates. Variant call concordance between three pairs of replicate samples, by variant type and cohort allele frequency. SNPs, single nucleotide polymorphisms; MAF, minor allele frequency; InDels, insertions and deletions.
(PDF)

S2 Table FEV_1_-associated variants in chr14q32.33. All variants in chr14q32.33 associated with FEV_1_ (% predicted) with p<1x10^-5^ (n = 82) in GWAS of 896 participants from APIC & URECA. N, number of genotyped individuals. MAF, minor allele frequency; 95% CI, 95% confidence interval; SE, standard error; P, P-value (Wald); FEV_1_, forced expiratory volume in one second; APIC, Asthma Phenotypes in the Inner City study; URECA, Urban Environment and Childhood Asthma study.
(PDF)

S3 Table MeQTL analysis results and associations with FEV_1_. All CpG sites where DNA methylation levels in NECs at age 11 in URECA were associated with rs10220464 at FDR<0.05 are shown with their corresponding associations with FEV_1_. The FDR-adjusted P-values (FDR Q) correspond to a 5% false-discovery rate. FDR, false discovery rate; 95% CI, 95% confidence interval; FEV_1_, forced expiratory volume in one second; URECA, Urban Environment and Childhood Asthma study.
(PDF)

S4 Table rs10220464 eQTL analysis results. Results of eQTL analyses in NECs and PBMCs with rs10220464 for all genes within 1 Mb in URECA. Gene expression was measured in counts per million mapped reads. The FDR-adjusted P-values (FDR Q) correspond to a 5% false-discovery rate. FDR, false discovery rate; 95% CI, 95% confidence interval; NECs, nasal epithelial cells; PBMCs, peripheral blood mononuclear cells; URECA, Urban Environment and Childhood Asthma study.
(PDF)

S5 Table Chromatin interactions with FEV_1_-associated SNPs. Bait and target fragments refer to mapped Hi-C restriction fragments on chr14 (hg38) for gene promoters and putative enhancers, respectively. FEV_1_ SNPs refer to number of FEV_1_-associated variants (p<1x10^-5^) within 1kb of target fragment. SNPs, single nucleotide polymorphisms; FEV_1_, forced expiratory volume in one second.
(PDF)

S6 Table Age at used lung function measure in URECA. URECA, Urban Environment and Childhood Asthma study; FEV_1_, forced expiratory volume in one second; FVC, forced vital capacity.
(PDF)

S7 Table Study samples. APIC, Asthma Phenotypes in the Inner City study; URECA, Urban Environment and Childhood Asthma study; WGS, whole-genome sequencing; NECs, nasal epithelial cells; PBMCs, peripheral blood mononuclear cells.
(PDF)

S8 Table Additional phenotypic, socioeconomic, and environmental data. Additional variables examined for potential confounding in mediation analyses for APIC & URECA. APIC, Asthma Phenotypes in the Inner City study; URECA, Urban Environment and Childhood Asthma study.
(PDF)

## References

[1] Hole DJ, Watt GC, Davey-Smith G, Hart CL, Gillis CR, Hawthorne VM. Impaired lung function and mortality risk in men and women: findings from the Renfrew and Paisley prospective population study. BMJ. 1996;313(7059):711–5; discussion 5–6. doi: 10.1136/bmj.313.7059.711 8819439 PMC2352103

[2] Schunemann HJ, Dorn J, Grant BJ, Winkelstein W Jr., Trevisan M. Pulmonary function is a long-term predictor of mortality in the general population: 29-year follow-up of the Buffalo Health Study. Chest. 2000;118(3):656–64. 10988186 10.1378/chest.118.3.656

[3] Mannino DM, Holguin F, Pavlin BI, Ferdinands JM. Risk factors for prevalence of and mortality related to restriction on spirometry: findings from the First National Health and Nutrition Examination Survey and follow-up. Int J Tuberc Lung Dis. 2005;9(6):613–21. 15971387

[4] Chinn S, Gislason T, Aspelund T, Gudnason V. Optimum expression of adult lung function based on all-cause mortality: results from the Reykjavik study. Respir Med. 2007;101(3):601–9. doi: 10.1016/j.rmed.2006.06.009 16889951

[5] Miller MR, Pedersen OF, Lange P, Vestbo J. Improved survival prediction from lung function data in a large population sample. Respir Med. 2009;103(3):442–8. doi: 10.1016/j.rmed.2008.09.016 18993043

[6] Agusti A, Noell G, Brugada J, Faner R. Lung function in early adulthood and health in later life: a transgenerational cohort analysis. Lancet Respir Med. 2017;5(12):935–45. doi: 10.1016/S2213-2600(17)30434-4 29150410

[7] Stern DA, Morgan WJ, Wright AL, Guerra S, Martinez FD. Poor airway function in early infancy and lung function by age 22 years: a non-selective longitudinal cohort study. Lancet. 2007;370(9589):758–64. doi: 10.1016/S0140-6736(07)61379-8 17765525 PMC2831283

[8] Stocks J, Hislop A, Sonnappa S. Early lung development: lifelong effect on respiratory health and disease. Lancet Respir Med. 2013;1(9):728–42. doi: 10.1016/S2213-2600(13)70118-8 24429276

[9] McGeachie MJ, Yates KP, Zhou X, Guo F, Sternberg AL, Van Natta ML, et al. Patterns of Growth and Decline in Lung Function in Persistent Childhood Asthma. N Engl J Med. 2016;374(19):1842–52. doi: 10.1056/NEJMoa1513737 27168434 PMC5032024

[10] Agusti A, Faner R. Lung function trajectories in health and disease. Lancet Respir Med. 2019;7(4):358–64. doi: 10.1016/S2213-2600(18)30529-0 30765254

[11] Bisgaard H, Jensen SM, Bonnelykke K. Interaction between asthma and lung function growth in early life. Am J Respir Crit Care Med. 2012;185(11):1183–9. doi: 10.1164/rccm.201110-1922OC 22461370

[12] Duijts L, Reiss IK, Brusselle G, de Jongste JC. Early origins of chronic obstructive lung diseases across the life course. Eur J Epidemiol. 2014;29(12):871–85. doi: 10.1007/s10654-014-9981-5 25537319

[13] Klimentidis YC, Vazquez AI, de Los Campos G, Allison DB, Dransfield MT, Thannickal VJ. Heritability of pulmonary function estimated from pedigree and whole-genome markers. Front Genet. 2013;4:174. doi: 10.3389/fgene.2013.00174 24058366 PMC3766834

[14] Wilk JB, Chen TH, Gottlieb DJ, Walter RE, Nagle MW, Brandler BJ, et al. A genome-wide association study of pulmonary function measures in the Framingham Heart Study. PLoS Genet. 2009;5(3):e1000429. doi: 10.1371/journal.pgen.1000429 19300500 PMC2652834

[15] Repapi E, Sayers I, Wain LV, Burton PR, Johnson T, Obeidat M, et al. Genome-wide association study identifies five loci associated with lung function. Nat Genet. 2010;42(1):36–44. doi: 10.1038/ng.501 20010834 PMC2862965

[16] Hancock DB, Eijgelsheim M, Wilk JB, Gharib SA, Loehr LR, Marciante KD, et al. Meta-analyses of genome-wide association studies identify multiple loci associated with pulmonary function. Nat Genet. 2010;42(1):45–52. doi: 10.1038/ng.500 20010835 PMC2832852

[17] Soler Artigas M, Loth DW, Wain LV, Gharib SA, Obeidat M, Tang W, et al. Genome-wide association and large-scale follow up identifies 16 new loci influencing lung function. Nat Genet. 2011;43(11):1082–90. doi: 10.1038/ng.941 21946350 PMC3267376

[18] Yao TC, Du G, Han L, Sun Y, Hu D, Yang JJ, et al. Genome-wide association study of lung function phenotypes in a founder population. J Allergy Clin Immunol. 2014;133(1):248–55 e1-10. doi: 10.1016/j.jaci.2013.06.018 23932459 PMC4270121

[19] Loth DW, Soler Artigas M, Gharib SA, Wain LV, Franceschini N, Koch B, et al. Genome-wide association analysis identifies six new loci associated with forced vital capacity. Nat Genet. 2014;46(7):669–77. doi: 10.1038/ng.3011 24929828 PMC4140093

[20] Wain LV, Shrine N, Artigas MS, Erzurumluoglu AM, Noyvert B, Bossini-Castillo L, et al. Genome-wide association analyses for lung function and chronic obstructive pulmonary disease identify new loci and potential druggable targets. Nat Genet. 2017;49(3):416–25. doi: 10.1038/ng.3787 28166213 PMC5326681

[21] Burkart KM, Sofer T, London SJ, Manichaikul A, Hartwig FP, Yan Q, et al. A Genome-Wide Association Study in Hispanics/Latinos Identifies Novel Signals for Lung Function. The Hispanic Community Health Study/Study of Latinos. Am J Respir Crit Care Med. 2018;198(2):208–19. doi: 10.1164/rccm.201707-1493OC 29394082 PMC6058984

[22] Wyss AB, Sofer T, Lee MK, Terzikhan N, Nguyen JN, Lahousse L, et al. Multiethnic meta-analysis identifies ancestry-specific and cross-ancestry loci for pulmonary function. Nat Commun. 2018;9(1):2976. doi: 10.1038/s41467-018-05369-0 30061609 PMC6065313

[23] Shrine N, Guyatt AL, Erzurumluoglu AM, Jackson VE, Hobbs BD, Melbourne CA, et al. New genetic signals for lung function highlight pathways and chronic obstructive pulmonary disease associations across multiple ancestries. Nat Genet. 2019;51(3):481–93. doi: 10.1038/s41588-018-0321-7 30804560 PMC6397078

[24] Akenroye AT, Brunetti T, Romero K, Daya M, Kanchan K, Shankar G, et al. Genome-wide association study of asthma, total IgE, and lung function in a cohort of Peruvian children. J Allergy Clin Immunol. 2021. doi: 10.1016/j.jaci.2021.02.035 33713768 PMC8429514

[25] Zhu Z, Li J, Si J, Ma B, Shi H, Lv J, et al. A large-scale genome-wide association analysis of lung function in the Chinese population identifies novel loci and highlights shared genetic aetiology with obesity. Eur Respir J. 2021;58(4). doi: 10.1183/13993003.00199-2021 33766948 PMC8513692

[26] Obeidat M, Hao K, Bosse Y, Nickle DC, Nie Y, Postma DS, et al. Molecular mechanisms underlying variations in lung function: a systems genetics analysis. Lancet Respir Med. 2015;3(10):782–95. doi: 10.1016/S2213-2600(15)00380-X 26404118 PMC5021067

[27] Gharib SA, Loth DW, Soler Artigas M, Birkland TP, Wilk JB, Wain LV, et al. Integrative pathway genomics of lung function and airflow obstruction. Hum Mol Genet. 2015;24(23):6836–48. doi: 10.1093/hmg/ddv378 26395457 PMC4643644

[28] Portas L, Pereira M, Shaheen SO, Wyss AB, London SJ, Burney PGJ, et al. Lung Development Genes and Adult Lung Function. Am J Respir Crit Care Med. 2020;202(6):853–65. doi: 10.1164/rccm.201912-2338OC 32392078 PMC7491406

[29] Kheirallah AK, Miller S, Hall IP, Sayers I. Translating Lung Function Genome-Wide Association Study (GWAS) Findings: New Insights for Lung Biology. Adv Genet. 2016;93:57–145. doi: 10.1016/bs.adgen.2015.12.002 26915270

[30] Aschard H, Tobin MD, Hancock DB, Skurnik D, Sood A, James A, et al. Evidence for large-scale gene-by-smoking interaction effects on pulmonary function. Int J Epidemiol. 2017;46(3):894–904. doi: 10.1093/ije/dyw318 28082375 PMC5837518

[31] Park B, An J, Kim W, Kang HY, Koh SB, Oh B, et al. Effect of 6p21 region on lung function is modified by smoking: a genome-wide interaction study. Sci Rep. 2020;10(1):13075. doi: 10.1038/s41598-020-70092-0 32753590 PMC7403370

[32] Kim W, Moll M, Qiao D, Hobbs BD, Shrine N, Sakornsakolpat P, et al. Smoking Interaction with a Polygenic Risk Score for Reduced Lung Function. medRxiv. 2021. doi: 10.1101/2021.03.26.21254415:2021.03.26.21254415 PMC8678715 34913977

[33] Melbourne CA, Erzurumluoglu AM, Shrine N, Chen J, Tobin MD, Hansell A, et al. Genome-wide gene-air pollution interaction analysis of lung function in 300,000 individuals. medRxiv. 2021. doi: 10.1016/j.envint.2021.107041 34923368 PMC8739564

[34] Miller MD, Marty MA. Impact of environmental chemicals on lung development. Environ Health Perspect. 2010;118(8):1155–64. doi: 10.1289/ehp.0901856 20444669 PMC2920089

[35] Decrue F, Gorlanova O, Usemann J, Frey U. Lung functional development and asthma trajectories. Semin Immunopathol. 2020;42(1):17–27. doi: 10.1007/s00281-020-00784-2 31989229

[36] He Z, Wu H, Zhang S, Lin Y, Li R, Xie L, et al. The association between secondhand smoke and childhood asthma: A systematic review and meta-analysis. Pediatr Pulmonol. 2020;55(10):2518–31. doi: 10.1002/ppul.24961 32667747

[37] Thacher JD, Schultz ES, Hallberg J, Hellberg U, Kull I, Thunqvist P, et al. Tobacco smoke exposure in early life and adolescence in relation to lung function. Eur Respir J. 2018;51(6). 10.1183/13993003.02111-2017 PMC6003782 29748304

[38] Dratva J, Zemp E, Dharmage SC, Accordini S, Burdet L, Gislason T, et al. Early Life Origins of Lung Ageing: Early Life Exposures and Lung Function Decline in Adulthood in Two European Cohorts Aged 28–73 Years. PLoS One. 2016;11(1):e0145127. doi: 10.1371/journal.pone.0145127 26811913 PMC4728209

[39] Savran O, Ulrik CS. Early life insults as determinants of chronic obstructive pulmonary disease in adult life. Int J Chron Obstruct Pulmon Dis. 2018;13:683–93. doi: 10.2147/COPD.S153555 29520136 PMC5834168

[40] Zeilinger S, Kuhnel B, Klopp N, Baurecht H, Kleinschmidt A, Gieger C, et al. Tobacco smoking leads to extensive genome-wide changes in DNA methylation. PLoS One. 2013;8(5):e63812. doi: 10.1371/journal.pone.0063812 23691101 PMC3656907

[41] Rider CF, Carlsten C. Air pollution and DNA methylation: effects of exposure in humans. Clin Epigenetics. 2019;11(1):131. doi: 10.1186/s13148-019-0713-2 31481107 PMC6724236

[42] Jamieson E, Korologou-Linden R, Wootton RE, Guyatt AL, Battram T, Burrows K, et al. Smoking, DNA Methylation, and Lung Function: a Mendelian Randomization Analysis to Investigate Causal Pathways. Am J Hum Genet. 2020;106(3):315–26. doi: 10.1016/j.ajhg.2020.01.015 32084330 PMC7058834

[43] Kwak SY, Park CY, Shin MJ. Smoking May Affect Pulmonary Function through DNA Methylation: an Epigenome-Wide Association Study in Korean Men. Clin Nutr Res. 2020;9(2):134–44. doi: 10.7762/cnr.2020.9.2.134 32395443 PMC7192668

[44] Sunny SK, Zhang H, Relton CL, Ring S, Kadalayil L, Mzayek F, et al. Sex-specific longitudinal association of DNA methylation with lung function. ERJ Open Res. 2021;7(3). doi: 10.1183/23120541.00127-2021 34235211 PMC8255542

[45] Mukherjee N, Lockett GA, Merid SK, Melen E, Pershagen G, Holloway JW, et al. DNA methylation and genetic polymorphisms of the Leptin gene interact to influence lung function outcomes and asthma at 18 years of age. Int J Mol Epidemiol Genet. 2016;7(1):1–17. 27186323 PMC4858611

[46] Zhang H, Tong X, Holloway JW, Rezwan FI, Lockett GA, Patil V, et al. The interplay of DNA methylation over time with Th2 pathway genetic variants on asthma risk and temporal asthma transition. Clin Epigenetics. 2014;6(1):8. doi: 10.1186/1868-7083-6-8 24735657 PMC4023182

[47] Munoz-Pizza DM, Villada-Canela M, Reyna MA, Texcalac-Sangrador JL, Osornio-Vargas AR. Air pollution and children’s respiratory health: a scoping review of socioeconomic status as an effect modifier. Int J Public Health. 2020;65(5):649–60. doi: 10.1007/s00038-020-01378-3 32405779

[48] Hajizadeh M, Nandi A. The socioeconomic gradient of secondhand smoke exposure in children: evidence from 26 low-income and middle-income countries. Tob Control. 2016;25(e2):e146–e55. doi: 10.1136/tobaccocontrol-2015-052828 27312823

[49] Martinez CH, Mannino DM, Curtis JL, Han MK, Diaz AA. Socioeconomic Characteristics Are Major Contributors to Ethnic Differences in Health Status in Obstructive Lung Disease: An Analysis of the National Health and Nutrition Examination Survey 2007–2010. Chest. 2015;148(1):151–8. doi: 10.1378/chest.14-1814 25633478 PMC4493871

[50] Thakur N, Oh SS, Nguyen EA, Martin M, Roth LA, Galanter J, et al. Socioeconomic status and childhood asthma in urban minority youths. The GALA II and SAGE II studies. Am J Respir Crit Care Med. 2013;188(10):1202–9. doi: 10.1164/rccm.201306-1016OC 24050698 PMC3863734

[51] Oraka E, Iqbal S, Flanders WD, Brinker K, Garbe P. Racial and ethnic disparities in current asthma and emergency department visits: findings from the National Health Interview Survey, 2001–2010. J Asthma. 2013;50(5):488–96. doi: 10.3109/02770903.2013.790417 23544662 PMC4566965

[52] Keet CA, Matsui EC, McCormack MC, Peng RD. Urban residence, neighborhood poverty, race/ethnicity, and asthma morbidity among children on Medicaid. J Allergy Clin Immunol. 2017;140(3):822–7. doi: 10.1016/j.jaci.2017.01.036 28283418 PMC8050806

[53] Manrai AK, Funke BH, Rehm HL, Olesen MS, Maron BA, Szolovits P, et al. Genetic Misdiagnoses and the Potential for Health Disparities. N Engl J Med. 2016;375(7):655–65. doi: 10.1056/NEJMsa1507092 27532831 PMC5292722

[54] Landry LG, Ali N, Williams DR, Rehm HL, Bonham VL. Lack Of Diversity In Genomic Databases Is A Barrier To Translating Precision Medicine Research Into Practice. Health Aff (Millwood). 2018;37(5):780–5. doi: 10.1377/hlthaff.2017.1595 29733732

[55] Pongracic JA, Krouse RZ, Babineau DC, Zoratti EM, Cohen RT, Wood RA, et al. Distinguishing characteristics of difficult-to-control asthma in inner-city children and adolescents. J Allergy Clin Immunol. 2016;138(4):1030–41. doi: 10.1016/j.jaci.2016.06.059 27720017 PMC5379996

[56] Zoratti EM, Krouse RZ, Babineau DC, Pongracic JA, O’Connor GT, Wood RA, et al. Asthma phenotypes in inner-city children. J Allergy Clin Immunol. 2016;138(4):1016–29. doi: 10.1016/j.jaci.2016.06.061 27720016 PMC5104222

[57] Gern JE, Visness CM, Gergen PJ, Wood RA, Bloomberg GR, O’Connor GT, et al. The Urban Environment and Childhood Asthma (URECA) birth cohort study: design, methods, and study population. BMC Pulm Med. 2009;9:17. doi: 10.1186/1471-2466-9-17 19426496 PMC2689166

[58] Zhou X, Stephens M. Genome-wide efficient mixed-model analysis for association studies. Nat Genet. 2012;44(7):821–4. doi: 10.1038/ng.2310 22706312 PMC3386377

[59] Consortium EP, Moore JE, Purcaro MJ, Pratt HE, Epstein CB, Shoresh N, et al. Expanded encyclopaedias of DNA elements in the human and mouse genomes. Nature. 2020;583(7818):699–710. doi: 10.1038/s41586-020-2493-4 32728249 PMC7410828

[60] Ma M, Ru Y, Chuang LS, Hsu NY, Shi LS, Hakenberg J, et al. Disease-associated variants in different categories of disease located in distinct regulatory elements. BMC Genomics. 2015;16 Suppl 8:S3. doi: 10.1186/1471-2164-16-S8-S3 26110593 PMC4480828

[61] Boyle EA, Li YI, Pritchard JK. An Expanded View of Complex Traits: From Polygenic to Omnigenic. Cell. 2017;169(7):1177–86. doi: 10.1016/j.cell.2017.05.038 28622505 PMC5536862

[62] Watanabe K, Stringer S, Frei O, Umicevic Mirkov M, de Leeuw C, Polderman TJC, et al. A global overview of pleiotropy and genetic architecture in complex traits. Nat Genet. 2019;51(9):1339–48. doi: 10.1038/s41588-019-0481-0 31427789

[63] Schulz H, Ruppert AK, Herms S, Wolf C, Mirza-Schreiber N, Stegle O, et al. Genome-wide mapping of genetic determinants influencing DNA methylation and gene expression in human hippocampus. Nat Commun. 2017;8(1):1511. doi: 10.1038/s41467-017-01818-4 29142228 PMC5688097

[64] Huan T, Joehanes R, Song C, Peng F, Guo Y, Mendelson M, et al. Genome-wide identification of DNA methylation QTLs in whole blood highlights pathways for cardiovascular disease. Nat Commun. 2019;10(1):4267. doi: 10.1038/s41467-019-12228-z 31537805 PMC6753136

[65] Hannon E, Gorrie-Stone TJ, Smart MC, Burrage J, Hughes A, Bao Y, et al. Leveraging DNA-Methylation Quantitative-Trait Loci to Characterize the Relationship between Methylomic Variation, Gene Expression, and Complex Traits. Am J Hum Genet. 2018;103(5):654–65. doi: 10.1016/j.ajhg.2018.09.007 30401456 PMC6217758

[66] McKennan C, Naughton K, Stanhope C, Kattan M, O’Connor GT, Sandel MT, et al. Longitudinal data reveal strong genetic and weak non-genetic components of ethnicity-dependent blood DNA methylation levels. Epigenetics. 2021;16(6):662–76. doi: 10.1080/15592294.2020.1817290 32997571 PMC8143220

[67] Joubert BR, Felix JF, Yousefi P, Bakulski KM, Just AC, Breton C, et al. DNA Methylation in Newborns and Maternal Smoking in Pregnancy: Genome-wide Consortium Meta-analysis. Am J Hum Genet. 2016;98(4):680–96. doi: 10.1016/j.ajhg.2016.02.019 27040690 PMC4833289

[68] Sikdar S, Joehanes R, Joubert BR, Xu CJ, Vives-Usano M, Rezwan FI, et al. Comparison of smoking-related DNA methylation between newborns from prenatal exposure and adults from personal smoking. Epigenomics. 2019;11(13):1487–500. doi: 10.2217/epi-2019-0066 31536415 PMC6836223

[69] Nicolae DL, Gamazon E, Zhang W, Duan S, Dolan ME, Cox NJ. Trait-associated SNPs are more likely to be eQTLs: annotation to enhance discovery from GWAS. PLoS Genet. 2010;6(4):e1000888. doi: 10.1371/journal.pgen.1000888 20369019 PMC2848547

[70] Yao DW, O’Connor LJ, Price AL, Gusev A. Quantifying genetic effects on disease mediated by assayed gene expression levels. Nat Genet. 2020;52(6):626–33. doi: 10.1038/s41588-020-0625-2 32424349 PMC7276299

[71] Hormozdiari F, van de Bunt M, Segre AV, Li X, Joo JWJ, Bilow M, et al. Colocalization of GWAS and eQTL Signals Detects Target Genes. Am J Hum Genet. 2016;99(6):1245–60. doi: 10.1016/j.ajhg.2016.10.003 27866706 PMC5142122

[72] Helling BA, Sobreira DR, Hansen GT, Sakabe NJ, Luo K, Billstrand C, et al. Altered transcriptional and chromatin responses to rhinovirus in bronchial epithelial cells from adults with asthma. Commun Biol. 2020;3(1):678. doi: 10.1038/s42003-020-01411-4 33188283 PMC7666152

[73] Samuels-Lev Y, O’Connor DJ, Bergamaschi D, Trigiante G, Hsieh JK, Zhong S, et al. ASPP proteins specifically stimulate the apoptotic function of p53. Mol Cell. 2001;8(4):781–94. doi: 10.1016/s1097-2765(01)00367-7 11684014

[74] Aylon Y, Ofir-Rosenfeld Y, Yabuta N, Lapi E, Nojima H, Lu X, et al. The Lats2 tumor suppressor augments p53-mediated apoptosis by promoting the nuclear proapoptotic function of ASPP1. Genes Dev. 2010;24(21):2420–9. doi: 10.1101/gad.1954410 21041410 PMC2964752

[75] Wang Y, Godin-Heymann N, Dan Wang X, Bergamaschi D, Llanos S, Lu X. ASPP1 and ASPP2 bind active RAS, potentiate RAS signalling and enhance p53 activity in cancer cells. Cell Death Differ. 2013;20(4):525–34. doi: 10.1038/cdd.2013.3 23392125 PMC3595493

[76] Xue H, Li MX. MicroRNA-150 protects against cigarette smoke-induced lung inflammation and airway epithelial cell apoptosis through repressing p53: MicroRNA-150 in CS-induced lung inflammation. Hum Exp Toxicol. 2018;37(9):920–8. doi: 10.1177/0960327117741749 29205062

[77] Xu F, Xu A, Guo Y, Bai Q, Wu X, Ji SP, et al. PM2.5 exposure induces alveolar epithelial cell apoptosis and causes emphysema through p53/Siva-1. Eur Rev Med Pharmacol Sci. 2020;24(7):3943–50. doi: 10.26355/eurrev_202004_20863 32329870

[78] Song Q, Zhou ZJ, Cai S, Chen Y, Chen P. Oxidative stress links the tumour suppressor p53 with cell apoptosis induced by cigarette smoke. Int J Environ Health Res. 2021. doi: 10.1080/09603123.2021.1910211 33825597

[79] Zhao K, Yu M, Zhu Y, Liu D, Wu Q, Hu Y. EGR-1/ASPP1 inter-regulatory loop promotes apoptosis by inhibiting cyto-protective autophagy. Cell Death Dis. 2017;8(6):e2869. doi: 10.1038/cddis.2017.268 28594407 PMC5520923

[80] Reynolds PR, Cosio MG, Hoidal JR. Cigarette smoke-induced Egr-1 upregulates proinflammatory cytokines in pulmonary epithelial cells. Am J Respir Cell Mol Biol. 2006;35(3):314–9. doi: 10.1165/rcmb.2005-0428OC 16601242 PMC2643284

[81] Chen ZH, Kim HP, Sciurba FC, Lee SJ, Feghali-Bostwick C, Stolz DB, et al. Egr-1 regulates autophagy in cigarette smoke-induced chronic obstructive pulmonary disease. PLoS One. 2008;3(10):e3316. doi: 10.1371/journal.pone.0003316 18830406 PMC2552992

[82] Shen N, Gong T, Wang JD, Meng FL, Qiao L, Yang RL, et al. Cigarette smoke-induced pulmonary inflammatory responses are mediated by EGR-1/GGPPS/MAPK signaling. Am J Pathol. 2011;178(1):110–8. doi: 10.1016/j.ajpath.2010.11.016 21224049 PMC3069843

[83] Wang SB, Zhang C, Xu XC, Xu F, Zhou JS, Wu YP, et al. Early growth response factor 1 is essential for cigarette smoke-induced MUC5AC expression in human bronchial epithelial cells. Biochem Biophys Res Commun. 2017;490(2):147–54. doi: 10.1016/j.bbrc.2017.06.014 28602698

[84] Xu F, Cao J, Luo M, Che L, Li W, Ying S, et al. Early growth response gene 1 is essential for urban particulate matter-induced inflammation and mucus hyperproduction in airway epithelium. Toxicol Lett. 2018;294:145–55. doi: 10.1016/j.toxlet.2018.05.003 29787794

[85] Golebski K, Gorenjak M, Kabesch M, Maitland-Van Der Zee A-H, Melén E, Potočnik U, et al. EGR-1 as a potential biomarker in asthma and proinflammatory responses in airway epithelium. European Respiratory Journal. 2021;58(suppl 65):PA2041.

[86] Wang A, Chiou J, Poirion OB, Buchanan J, Valdez MJ, Verheyden JM, et al. Single-cell multiomic profiling of human lungs reveals cell-type-specific and age-dynamic control of SARS-CoV2 host genes. Elife. 2020;9. doi: 10.7554/eLife.62522 33164753 PMC7688309

[87] Karlsson M, Zhang C, Mear L, Zhong W, Digre A, Katona B, et al. A single-cell type transcriptomics map of human tissues. Sci Adv. 2021;7(31). doi: 10.1126/sciadv.abh2169 34321199 PMC8318366

[88] Cheng Y, Luo W, Li Z, Cao M, Zhu Z, Han C, et al. CircRNA-012091/PPP1R13B-mediated Lung Fibrotic Response in Silicosis via Endoplasmic Reticulum Stress and Autophagy. Am J Respir Cell Mol Biol. 2019;61(3):380–91. doi: 10.1165/rcmb.2019-0017OC 30908929

[89] Vigneron AM, Ludwig RL, Vousden KH. Cytoplasmic ASPP1 inhibits apoptosis through the control of YAP. Genes Dev. 2010;24(21):2430–9. doi: 10.1101/gad.1954310 21041411 PMC2964753

[90] Manfredi JJ. An identity crisis for a cancer gene: subcellular location determines ASPP1 function. Cancer Cell. 2010;18(5):409–10. doi: 10.1016/j.ccr.2010.11.003 21075306 PMC3001292

[91] Fogal V, Kartasheva NN, Trigiante G, Llanos S, Yap D, Vousden KH, et al. ASPP1 and ASPP2 are new transcriptional targets of E2F. Cell Death Differ. 2005;12(4):369–76. doi: 10.1038/sj.cdd.4401562 15731768

[92] Zhou SJ, Li M, Zeng DX, Zhu ZM, Hu XW, Li YH, et al. Expression variations of connective tissue growth factor in pulmonary arteries from smokers with and without chronic obstructive pulmonary disease. Sci Rep. 2015;5:8564. doi: 10.1038/srep08564 25708588 PMC4338434

[93] Eguchi A, Nishizawa-Jotaki S, Tanabe H, Rahmutulla B, Watanabe M, Miyaso H, et al. An Altered DNA Methylation Status in the Human Umbilical Cord Is Correlated with Maternal Exposure to Polychlorinated Biphenyls. Int J Environ Res Public Health. 2019;16(15). doi: 10.3390/ijerph16152786 31382687 PMC6696183

[94] Pierce BL, Tong L, Argos M, Demanelis K, Jasmine F, Rakibuz-Zaman M, et al. Co-occurring expression and methylation QTLs allow detection of common causal variants and shared biological mechanisms. Nat Commun. 2018;9(1):804. doi: 10.1038/s41467-018-03209-9 29476079 PMC5824840

[95] Philibert RA, Sears RA, Powers LS, Nash E, Bair T, Gerke AK, et al. Coordinated DNA methylation and gene expression changes in smoker alveolar macrophages: specific effects on VEGF receptor 1 expression. J Leukoc Biol. 2012;92(3):621–31. doi: 10.1189/jlb.1211632 22427682 PMC3427615

[96] Guijo M, Ceballos-Chavez M, Gomez-Marin E, Basurto-Cayuela L, Reyes JC. Expression of TDRD9 in a subset of lung carcinomas by CpG island hypomethylation protects from DNA damage. Oncotarget. 2018;9(11):9618–31. doi: 10.18632/oncotarget.22709 29515758 PMC5839389

[97] Consortium GT. The Genotype-Tissue Expression (GTEx) project. Nat Genet. 2013;45(6):580–5. doi: 10.1038/ng.2653 23715323 PMC4010069

[98] Lokk K, Modhukur V, Rajashekar B, Martens K, Magi R, Kolde R, et al. DNA methylome profiling of human tissues identifies global and tissue-specific methylation patterns. Genome Biol. 2014;15(4):r54. doi: 10.1186/gb-2014-15-4-r54 24690455 PMC4053947

[99] Wan J, Oliver VF, Wang G, Zhu H, Zack DJ, Merbs SL, et al. Characterization of tissue-specific differential DNA methylation suggests distinct modes of positive and negative gene expression regulation. BMC Genomics. 2015;16:49. doi: 10.1186/s12864-015-1271-4 25652663 PMC4331481

[100] den Dekker HT, Burrows K, Felix JF, Salas LA, Nedeljkovic I, Yao J, et al. Newborn DNA-methylation, childhood lung function, and the risks of asthma and COPD across the life course. Eur Respir J. 2019;53(4). doi: 10.1183/13993003.01795-2018 30765504

[101] Imboden M, Wielscher M, Rezwan FI, Amaral AFS, Schaffner E, Jeong A, et al. Epigenome-wide association study of lung function level and its change. Eur Respir J. 2019;54(1). doi: 10.1183/13993003.00457-2019 31073081 PMC6610463

[102] Mukherjee N, Arathimos R, Chen S, Kheirkhah Rahimabad P, Han L, Zhang H, et al. DNA methylation at birth is associated with lung function development until age 26 years. Eur Respir J. 2021;57(4). 10.1183/13993003.03505-2020 33214203

[103] Wang T, Wang W, Li W, Duan H, Xu C, Tian X, et al. Genome-wide DNA methylation analysis of pulmonary function in middle and old-aged Chinese monozygotic twins. Respir Res. 2021;22(1):300. doi: 10.1186/s12931-021-01896-5 34809630 PMC8609861

[104] Herrera-Luis E, Li A, Mak ACY, Perez-Garcia J, Elhawary JR, Oh SS, et al. Epigenome-wide association study of lung function in Latino children and youth with asthma. Clin Epigenetics. 2022;14(1):9. doi: 10.1186/s13148-022-01227-5 35033200 PMC8760660

[105] Cosin-Tomas M, Bustamante M, Sunyer J. Epigenetic association studies at birth and the origin of lung function development. Eur Respir J. 2021;57(4). doi: 10.1183/13993003.00109-2021 33858853

[106] Yang IV, Lozupone CA, Schwartz DA. The environment, epigenome, and asthma. J Allergy Clin Immunol. 2017;140(1):14–23. doi: 10.1016/j.jaci.2017.05.011 28673400 PMC5673130

[107] Lin PI, Shu H, Mersha TB. Comparing DNA methylation profiles across different tissues associated with the diagnosis of pediatric asthma. Sci Rep. 2020;10(1):151. doi: 10.1038/s41598-019-56310-4 31932625 PMC6957523

[108] Richmond RC, Davey Smith G. Mendelian Randomization: Concepts and Scope. Cold Spring Harb Perspect Med. 2022;12(1). doi: 10.1101/cshperspect.a040501 34426474 PMC8725623

[109] Robins JM, Greenland S. Identifiability and exchangeability for direct and indirect effects. Epidemiology. 1992;3(2):143–55. doi: 10.1097/00001648-199203000-00013 1576220

[110] Li YF, Gilliland FD, Berhane K, McConnell R, Gauderman WJ, Rappaport EB, et al. Effects of in utero and environmental tobacco smoke exposure on lung function in boys and girls with and without asthma. Am J Respir Crit Care Med. 2000;162(6):2097–104. doi: 10.1164/ajrccm.162.6.2004178 11112121

[111] Gilliland FD, Berhane K, Li YF, Rappaport EB, Peters JM. Effects of early onset asthma and in utero exposure to maternal smoking on childhood lung function. Am J Respir Crit Care Med. 2003;167(6):917–24. doi: 10.1164/rccm.200206-616OC 12480608

[112] Schultz ES, Litonjua AA, Melen E. Effects of Long-Term Exposure to Traffic-Related Air Pollution on Lung Function in Children. Curr Allergy Asthma Rep. 2017;17(6):41. doi: 10.1007/s11882-017-0709-y 28551888 PMC5446841

[113] Kreiner-Moller E, Bisgaard H, Bonnelykke K. Prenatal and postnatal genetic influence on lung function development. J Allergy Clin Immunol. 2014;134(5):1036–42 e15. doi: 10.1016/j.jaci.2014.04.003 24857373

[114] Lee EY, Mak ACY, Hu D, Sajuthi S, White MJ, Keys KL, et al. Whole-Genome Sequencing Identifies Novel Functional Loci Associated with Lung Function in Puerto Rican Youth. Am J Respir Crit Care Med. 2020;202(7):962–72. doi: 10.1164/rccm.202002-0351OC 32459537 PMC7528787

[115] Goddard PC, Keys KL, Mak ACY, Lee EY, Liu AK, Samedy-Bates LA, et al. Integrative genomic analysis in African American children with asthma finds three novel loci associated with lung function. Genet Epidemiol. 2021;45(2):190–208. doi: 10.1002/gepi.22365 32989782 PMC7902343

[116] Akenroye AT, Brunetti T, Romero K, Daya M, Kanchan K, Shankar G, et al. Genome-wide association study of asthma, total IgE, and lung function in a cohort of Peruvian children. J Allergy Clin Immunol. 2021;148(6):1493–504. doi: 10.1016/j.jaci.2021.02.035 33713768 PMC8429514

[117] Imboden M, Bouzigon E, Curjuric I, Ramasamy A, Kumar A, Hancock DB, et al. Genome-wide association study of lung function decline in adults with and without asthma. J Allergy Clin Immunol. 2012;129(5):1218–28. doi: 10.1016/j.jaci.2012.01.074 22424883 PMC3340499

[118] Aschard H, Vilhjalmsson BJ, Joshi AD, Price AL, Kraft P. Adjusting for heritable covariates can bias effect estimates in genome-wide association studies. Am J Hum Genet. 2015;96(2):329–39. doi: 10.1016/j.ajhg.2014.12.021 25640676 PMC4320269

[119] Jones MJ, Goodman SJ, Kobor MS. DNA methylation and healthy human aging. Aging Cell. 2015;14(6):924–32. doi: 10.1111/acel.12349 25913071 PMC4693469

[120] Ambatipudi S, Cuenin C, Hernandez-Vargas H, Ghantous A, Le Calvez-Kelm F, Kaaks R, et al. Tobacco smoking-associated genome-wide DNA methylation changes in the EPIC study. Epigenomics. 2016;8(5):599–618. doi: 10.2217/epi-2016-0001 26864933

[121] Tommasi S, Zheng A, Besaratinia A. Exposure of mice to secondhand smoke elicits both transient and long-lasting transcriptional changes in cancer-related functional networks. Int J Cancer. 2015;136(10):2253–63. doi: 10.1002/ijc.29284 25346222

[122] Sridhar S, Schembri F, Zeskind J, Shah V, Gustafson AM, Steiling K, et al. Smoking-induced gene expression changes in the bronchial airway are reflected in nasal and buccal epithelium. BMC Genomics. 2008;9:259. doi: 10.1186/1471-2164-9-259 18513428 PMC2435556

[123] Zhang X, Sebastiani P, Liu G, Schembri F, Zhang X, Dumas YM, et al. Similarities and differences between smoking-related gene expression in nasal and bronchial epithelium. Physiol Genomics. 2010;41(1):1–8. doi: 10.1152/physiolgenomics.00167.2009 19952278 PMC2841495

[124] Brugha R, Lowe R, Henderson AJ, Holloway JW, Rakyan V, Wozniak E, et al. DNA methylation profiles between airway epithelium and proxy tissues in children. Acta Paediatr. 2017;106(12):2011–6. doi: 10.1111/apa.14027 28833606

[125] Imkamp K, Berg M, Vermeulen CJ, Heijink IH, Guryev V, Kerstjens HAM, et al. Nasal epithelium as a proxy for bronchial epithelium for smoking-induced gene expression and expression Quantitative Trait Loci. J Allergy Clin Immunol. 2018;142(1):314–7 e15. doi: 10.1016/j.jaci.2018.01.047 29522853

[126] Kicic A, de Jong E, Ling KM, Nichol K, Anderson D, Wark PAB, et al. Assessing the unified airway hypothesis in children via transcriptional profiling of the airway epithelium. J Allergy Clin Immunol. 2020;145(6):1562–73. doi: 10.1016/j.jaci.2020.02.018 32113981

[127] Bergougnoux A, Claustres M, De Sario A. Nasal epithelial cells: a tool to study DNA methylation in airway diseases. Epigenomics. 2015;7(1):119–26. doi: 10.2217/epi.14.65 25687471

[128] Stoffel B, Sorkness C, Pech C. Use of a Single, Independent IRB: Case Study of an NIH Funded Consortium. Contemp Clin Trials Commun. 2017;8:114–21. doi: 10.1016/j.conctc.2017.09.001 29546249 PMC5846487

[129] Gergen PJ, Teach SJ, Togias A, Busse WW. Reducing Exacerbations in the Inner City: Lessons from the Inner-City Asthma Consortium (ICAC). J Allergy Clin Immunol Pract. 2016;4(1):22–6. doi: 10.1016/j.jaip.2015.07.024 26589178 PMC4715939

[130] O’Connor GT, Lynch SV, Bloomberg GR, Kattan M, Wood RA, Gergen PJ, et al. Early-life home environment and risk of asthma among inner-city children. J Allergy Clin Immunol. 2018;141(4):1468–75. doi: 10.1016/j.jaci.2017.06.040 28939248 PMC6521865

[131] Quanjer PH, Stanojevic S, Cole TJ, Baur X, Hall GL, Culver BH, et al. Multi-ethnic reference values for spirometry for the 3-95-yr age range: the global lung function 2012 equations. Eur Respir J. 2012;40(6):1324–43. doi: 10.1183/09031936.00080312 22743675 PMC3786581

[132] Bernert JT, Harmon TL, Sosnoff CS, McGuffey JE. Use of continine immunoassay test strips for preclassifying urine samples from smokers and nonsmokers prior to analysis by LC-MS-MS. J Anal Toxicol. 2005;29(8):814–8. doi: 10.1093/jat/29.8.814 16374940

[133] Regier AA, Farjoun Y, Larson DE, Krasheninina O, Kang HM, Howrigan DP, et al. Functional equivalence of genome sequencing analysis pipelines enables harmonized variant calling across human genetics projects. Nat Commun. 2018;9(1):4038. doi: 10.1038/s41467-018-06159-4 30279509 PMC6168605

[134] Kowalski MH, Qian H, Hou Z, Rosen JD, Tapia AL, Shan Y, et al. Use of >100,000 NHLBI Trans-Omics for Precision Medicine (TOPMed) Consortium whole genome sequences improves imputation quality and detection of rare variant associations in admixed African and Hispanic/Latino populations. PLoS Genet. 2019;15(12):e1008500. 31869403 10.1371/journal.pgen.1008500 PMC6953885

[135] Zhang F, Flickinger M, Taliun SAG, In PPGC, Abecasis GR, Scott LJ, et al. Ancestry-agnostic estimation of DNA sample contamination from sequence reads. Genome Res. 2020;30(2):185–94. doi: 10.1101/gr.246934.118 31980570 PMC7050530

[136] Chang CC, Chow CC, Tellier LC, Vattikuti S, Purcell SM, Lee JJ. Second-generation PLINK: rising to the challenge of larger and richer datasets. Gigascience. 2015;4:7. doi: 10.1186/s13742-015-0047-8 25722852 PMC4342193

[137] McKennan C, Naughton K, Stanhope C, Kattan M, O’Connor GT, Sandel MT, et al. Longitudinal data reveal strong genetic and weak non-genetic components of ethnicity-dependent blood DNA methylation levels. Epigenetics. 2020. doi: 10.1080/15592294.2020.1817290 32997571 PMC8143220

[138] Jun G, Flickinger M, Hetrick KN, Romm JM, Doheny KF, Abecasis GR, et al. Detecting and estimating contamination of human DNA samples in sequencing and array-based genotype data. Am J Hum Genet. 2012;91(5):839–48. doi: 10.1016/j.ajhg.2012.09.004 23103226 PMC3487130

[139] Hao W, Storey JD. Extending Tests of Hardy-Weinberg Equilibrium to Structured Populations. Genetics. 2019;213(3):759–70. doi: 10.1534/genetics.119.302370 31537622 PMC6827367

[140] Danecek P, Auton A, Abecasis G, Albers CA, Banks E, DePristo MA, et al. The variant call format and VCFtools. Bioinformatics. 2011;27(15):2156–8. doi: 10.1093/bioinformatics/btr330 21653522 PMC3137218

[141] Danecek P, Bonfield JK, Liddle J, Marshall J, Ohan V, Pollard MO, et al. Twelve years of SAMtools and BCFtools. Gigascience. 2021;10(2). doi: 10.1093/gigascience/giab008 33590861 PMC7931819

[142] Genomes Project C, Auton A, Brooks LD, Durbin RM, Garrison EP, Kang HM, et al. A global reference for human genetic variation. Nature. 2015;526(7571):68–74. doi: 10.1038/nature15393 26432245 PMC4750478

[143] Bergstrom A, McCarthy SA, Hui R, Almarri MA, Ayub Q, Danecek P, et al. Insights into human genetic variation and population history from 929 diverse genomes. Science. 2020;367(6484). doi: 10.1126/science.aay5012 32193295 PMC7115999

[144] Alexander DH, Novembre J, Lange K. Fast model-based estimation of ancestry in unrelated individuals. Genome Res. 2009;19(9):1655–64. doi: 10.1101/gr.094052.109 19648217 PMC2752134

[145] Conomos MP, Miller MB, Thornton TA. Robust inference of population structure for ancestry prediction and correction of stratification in the presence of relatedness. Genet Epidemiol. 2015;39(4):276–93. doi: 10.1002/gepi.21896 25810074 PMC4836868

[146] Conomos MP, Reiner AP, Weir BS, Thornton TA. Model-free Estimation of Recent Genetic Relatedness. Am J Hum Genet. 2016;98(1):127–48. doi: 10.1016/j.ajhg.2015.11.022 26748516 PMC4716688

[147] Manichaikul A, Mychaleckyj JC, Rich SS, Daly K, Sale M, Chen WM. Robust relationship inference in genome-wide association studies. Bioinformatics. 2010;26(22):2867–73. doi: 10.1093/bioinformatics/btq559 20926424 PMC3025716

[148] Shringarpure SS, Bustamante CD, Lange K, Alexander DH. Efficient analysis of large datasets and sex bias with ADMIXTURE. BMC Bioinformatics. 2016;17:218. doi: 10.1186/s12859-016-1082-x 27216439 PMC4877806

[149] Sofer T, Zheng X, Gogarten SM, Laurie CA, Grinde K, Shaffer JR, et al. A fully adjusted two-stage procedure for rank-normalization in genetic association studies. Genet Epidemiol. 2019;43(3):263–75. doi: 10.1002/gepi.22188 30653739 PMC6416071

[150] McCaw ZR, Lane JM, Saxena R, Redline S, Lin X. Operating characteristics of the rank-based inverse normal transformation for quantitative trait analysis in genome-wide association studies. Biometrics. 2020;76(4):1262–72. doi: 10.1111/biom.13214 31883270 PMC8643141

[151] Wang G, Sarkar A, Carbonetto P, Stephens M. A simple new approach to variable selection in regression, with application to genetic fine mapping. Journal of the Royal Statistical Society: Series B (Statistical Methodology). 2020;82(5):1273–300. 10.1111/rssb.12388 PMC10201948 37220626

[152] Shabalin AA. Matrix eQTL: ultra fast eQTL analysis via large matrix operations. Bioinformatics. 2012;28(10):1353–8. doi: 10.1093/bioinformatics/bts163 22492648 PMC3348564

[153] McKennan C, Nicolae D. Accounting for unobserved covariates with varying degrees of estimability in high-dimensional biological data. Biometrika. 2019;106(4):823–40. doi: 10.1093/biomet/asz037 31754283 PMC6845853

[154] Zhang D. A Coefficient of Determination for Generalized Linear Models. The American Statistician. 2017;71(4):310–6.

[155] Fox J, Kleiber C, Zeileis A. ivreg: Two-Stage Least-Squares Regression with Diagnostics. https://john-d-fox.github.io/ivreg/2021.

[156] Burgess S, Small DS, Thompson SG. A review of instrumental variable estimators for Mendelian randomization. Stat Methods Med Res. 2017;26(5):2333–55. doi: 10.1177/0962280215597579 26282889 PMC5642006

[157] Burgess S, Thompson SG. Use of allele scores as instrumental variables for Mendelian randomization. International Journal of Epidemiology. 2013;42(4):1134–44. doi: 10.1093/ije/dyt093 24062299 PMC3780999

[158] Alfons A, Ateş NY, Groenen PJF. A Robust Bootstrap Test for Mediation Analysis. Organizational Research Methods.0(0):1094428121999096.

[159] Dobin A, Davis CA, Schlesinger F, Drenkow J, Zaleski C, Jha S, et al. STAR: ultrafast universal RNA-seq aligner. Bioinformatics. 2013;29(1):15–21. doi: 10.1093/bioinformatics/bts635 23104886 PMC3530905

[160] Law CW, Chen Y, Shi W, Smyth GK. voom: Precision weights unlock linear model analysis tools for RNA-seq read counts. Genome Biol. 2014;15(2):R29. doi: 10.1186/gb-2014-15-2-r29 24485249 PMC4053721

[161] Ritchie ME, Phipson B, Wu D, Hu Y, Law CW, Shi W, et al. limma powers differential expression analyses for RNA-sequencing and microarray studies. Nucleic Acids Res. 2015;43(7):e47. doi: 10.1093/nar/gkv007 25605792 PMC4402510

[162] Jager R, Migliorini G, Henrion M, Kandaswamy R, Speedy HE, Heindl A, et al. Capture Hi-C identifies the chromatin interactome of colorectal cancer risk loci. Nat Commun. 2015;6:6178. doi: 10.1038/ncomms7178 25695508 PMC4346635

[163] Mifsud B, Tavares-Cadete F, Young AN, Sugar R, Schoenfelder S, Ferreira L, et al. Mapping long-range promoter contacts in human cells with high-resolution capture Hi-C. Nat Genet. 2015;47(6):598–606. doi: 10.1038/ng.3286 25938943

[164] Montefiori LE, Sobreira DR, Sakabe NJ, Aneas I, Joslin AC, Hansen GT, et al. A promoter interaction map for cardiovascular disease genetics. Elife. 2018;7. doi: 10.7554/eLife.35788 29988018 PMC6053306

[165] Cairns J, Freire-Pritchett P, Wingett SW, Varnai C, Dimond A, Plagnol V, et al. CHiCAGO: robust detection of DNA looping interactions in Capture Hi-C data. Genome Biol. 2016;17(1):127. doi: 10.1186/s13059-016-0992-2 27306882 PMC4908757

