# Supplementary figures and images for: Multi-omic association study identifies DNA methylation-mediated genotype and smoking exposure effects on lung function in children living in urban settings
Source: PLoS Genet. 2023 Jan 13;19(1):e1010594. doi: 10.1371/journal.pgen.1010594 (PMC9879483; doi:10.1371/journal.pgen.1010594)

**A**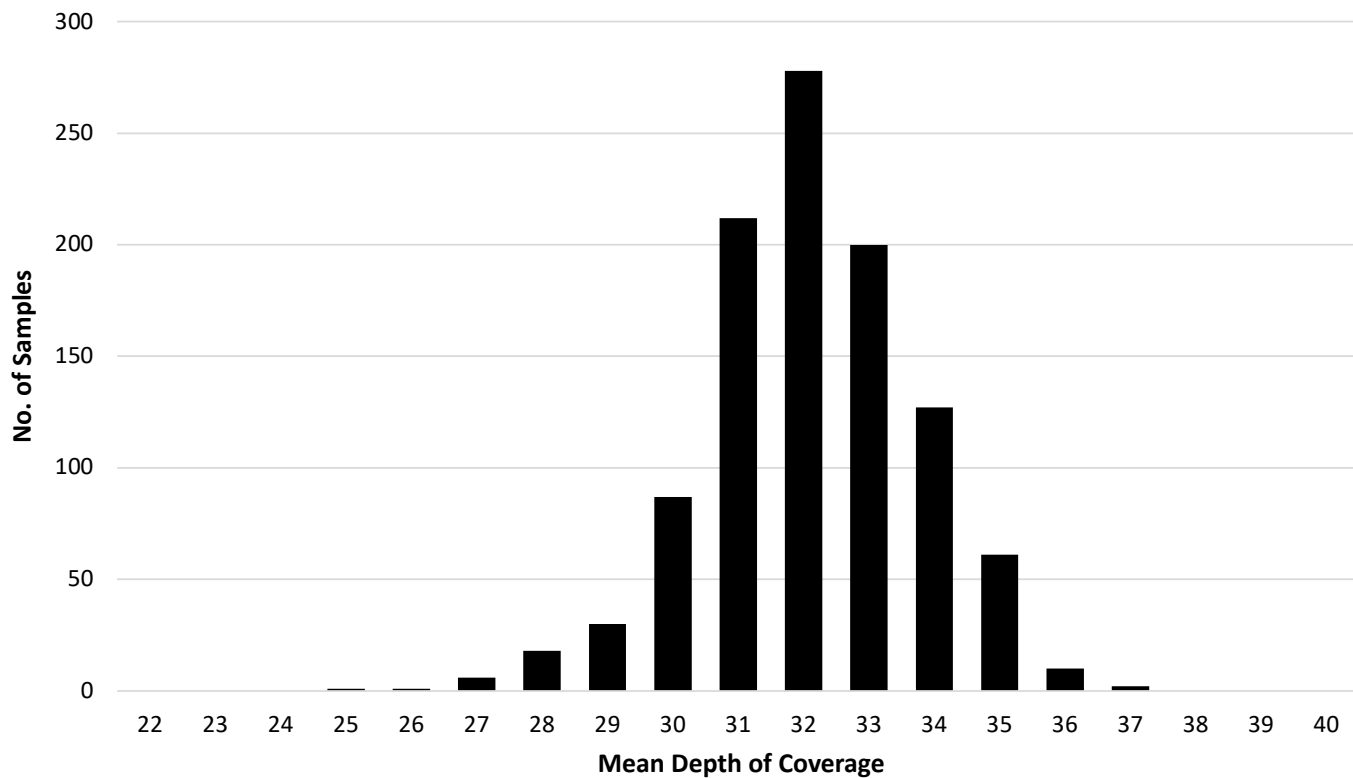**B**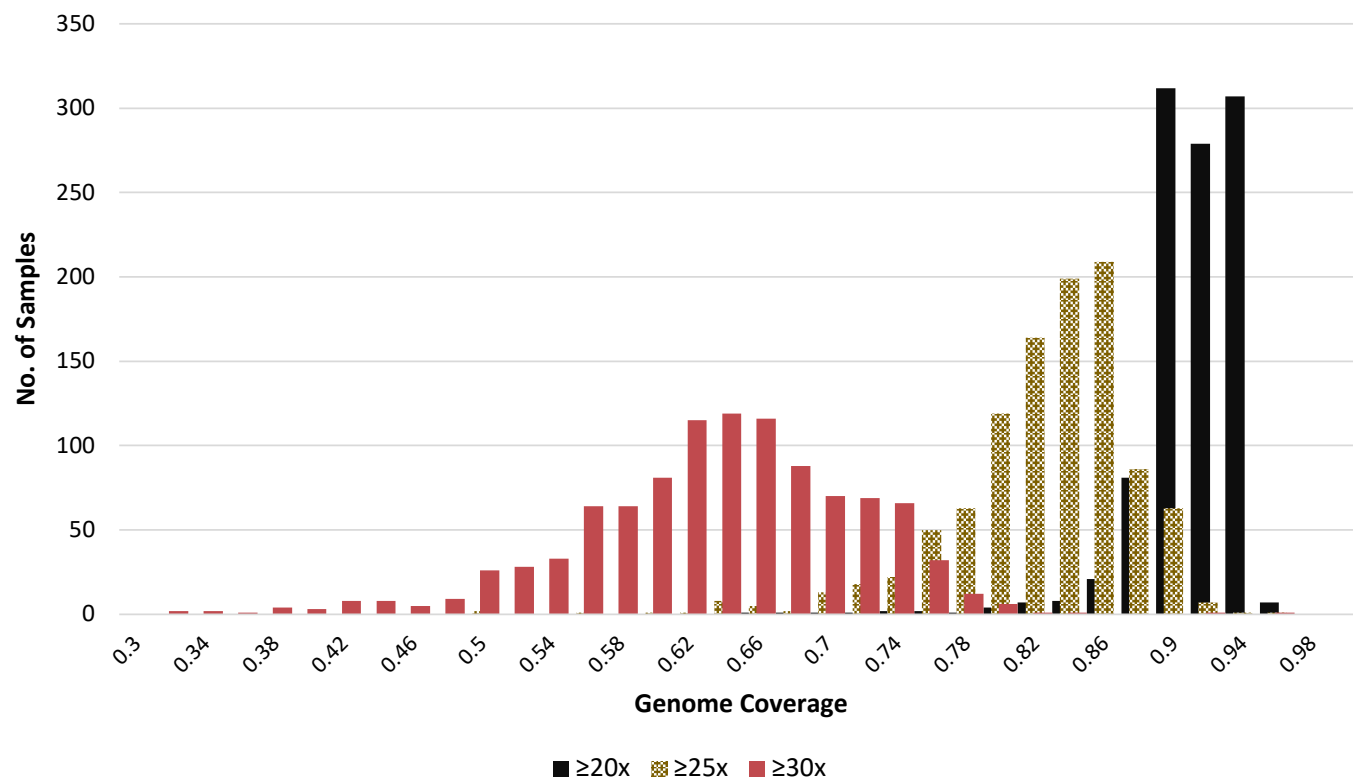

Supplement: S1 Fig — A) Histogram of 1,035 whole-genome sequencing (WGS) samples from APIC and URECA by mean depth of coverage. B) Histogram of WGS samples based on proportion of genome covered at 20x, 25x, and 30x depth. APIC, Asthma Phenotypes in the Inner City study; URECA, Urban Environment and Childhood Asthma study. (PDF) [file pgen.1010594.s001.pdf]

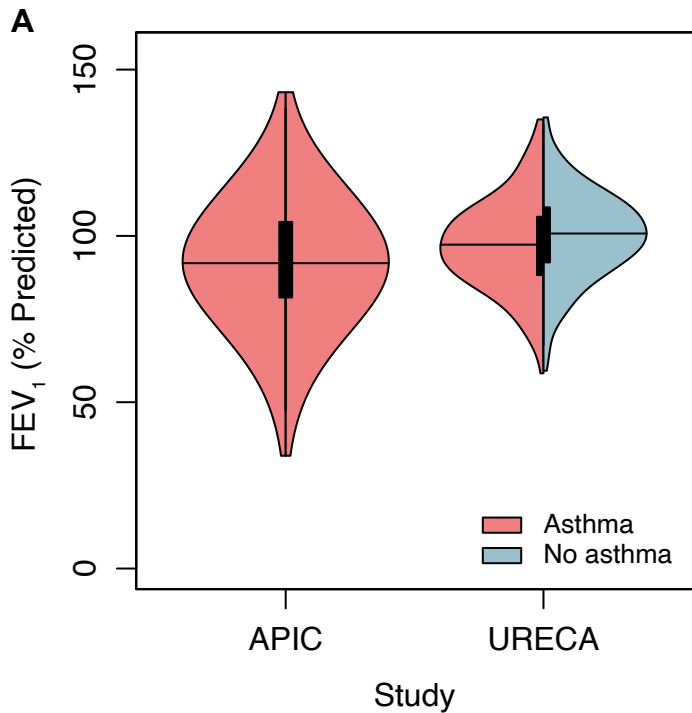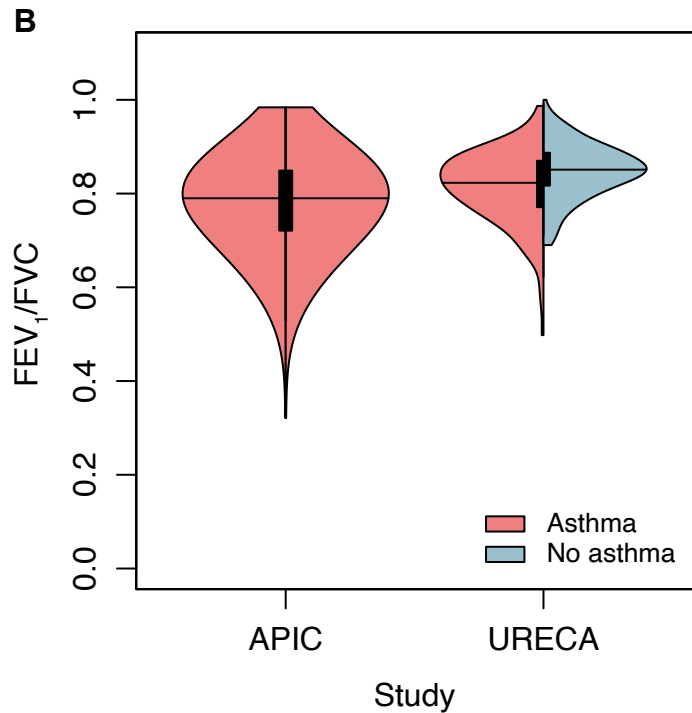

Supplement: S2 Fig — A) Distribution of FEV1 (% predicted) in APIC and URECA. B) Distribution of FEV1/FVC in APIC and URECA. APIC, Asthma Phenotypes in the Inner City study; URECA, Urban Environment and Childhood Asthma study. FEV1, forced expiratory volume in one second; FVC, forced vital capacity. (PDF) [file pgen.1010594.s002.pdf]

**A****FEV<sub>1</sub> (% predicted)**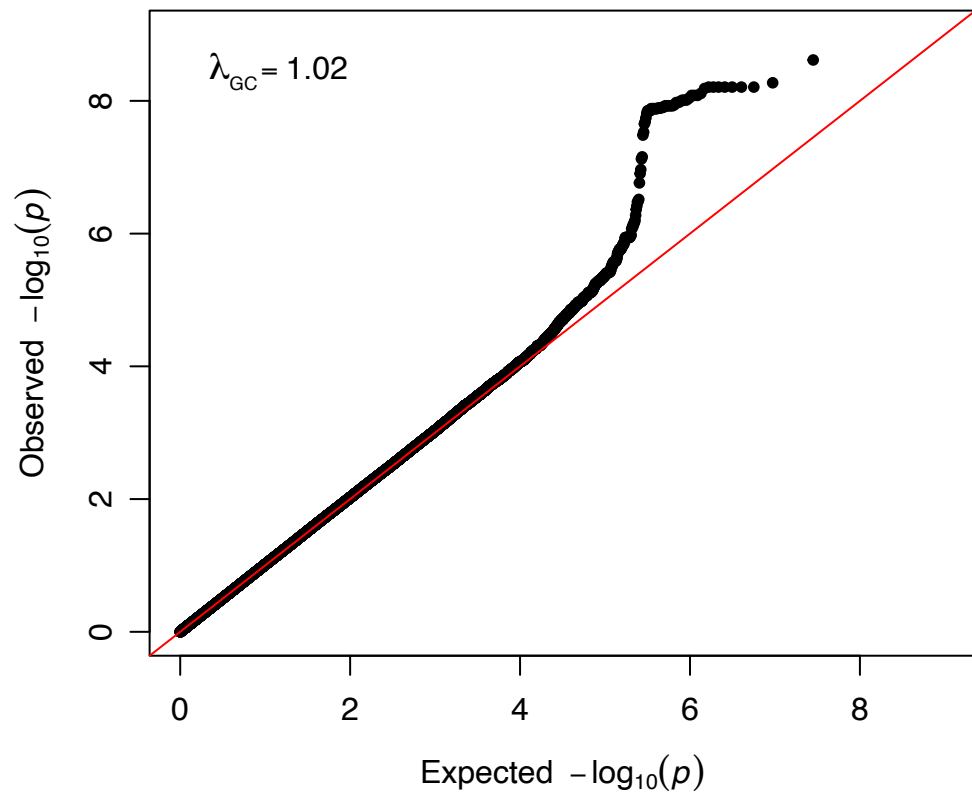**B****FEV<sub>1</sub>/FVC (Z-score)**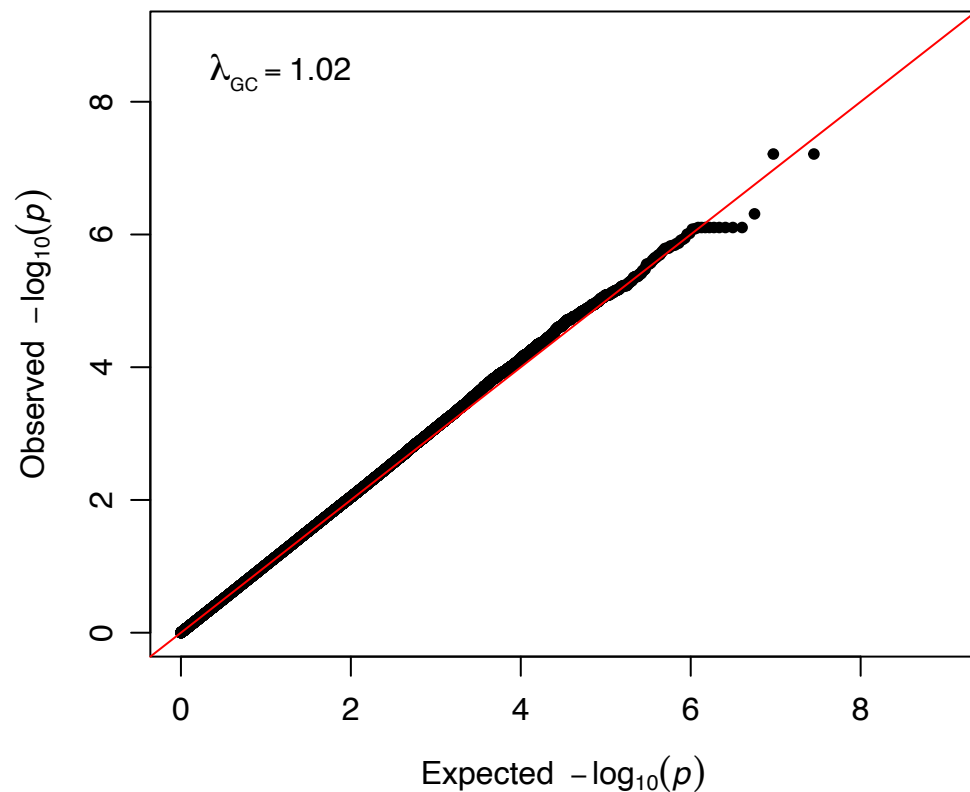

Supplement: S3 Fig — Quantile-quantile plots of the GWAS results with corresponding genomic control factors (lambda) are shown for A) FEV1 (% predicted) and B) FEV1/FVC. FEV1, forced expiratory volume in one second; FVC, forced vital capacity. (PDF) [file pgen.1010594.s003.pdf]

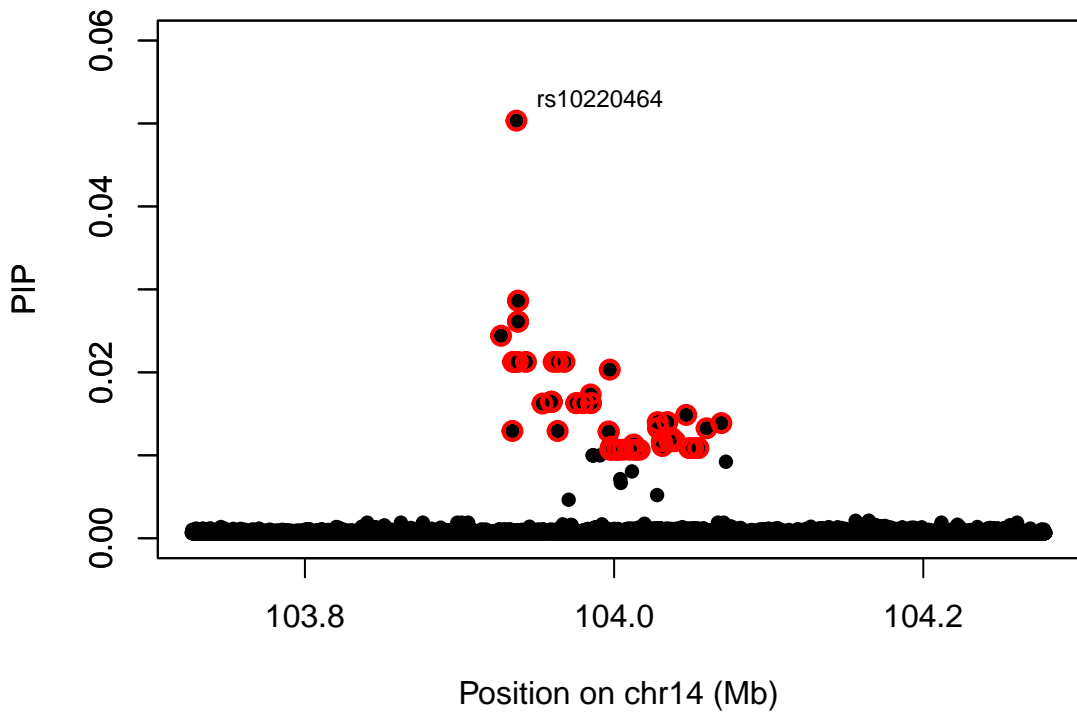

Supplement: S4 Fig — The X-axis shows the chromosome position on chromosome 14. The Y-axis is the posterior inclusion probability (PIP). Variants highlighted in red represent a credible set, in which there is a 95% probability that at least one of the variants is causal. FEV1, forced expiratory volume in one second. (PDF) [file pgen.1010594.s004.pdf]

**A****FEV<sub>1</sub> (% Predicted)**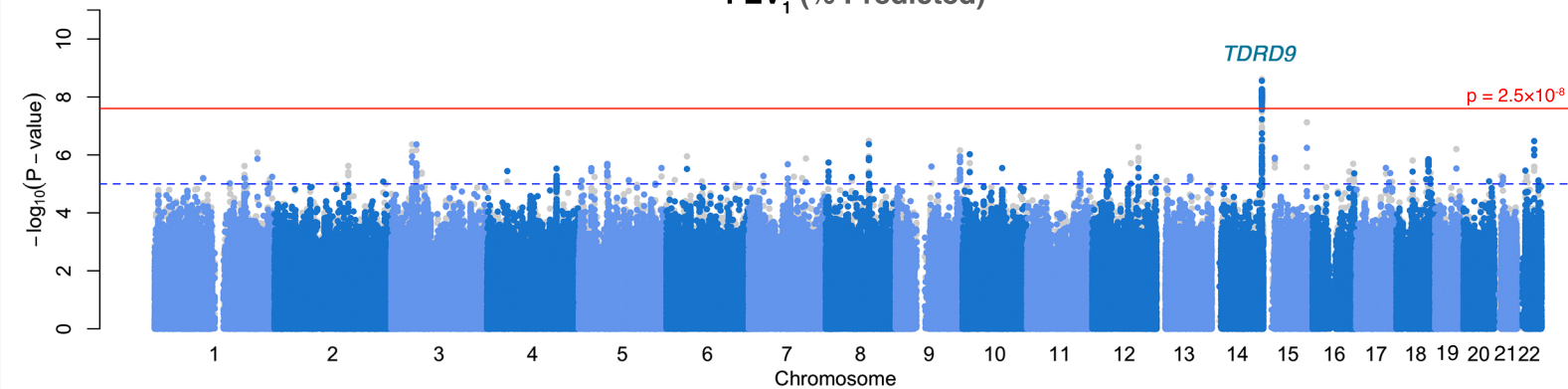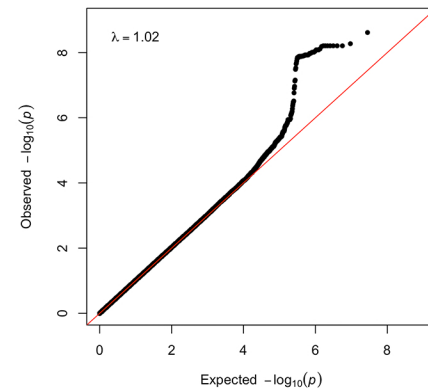**B****FEV<sub>1</sub>/FVC**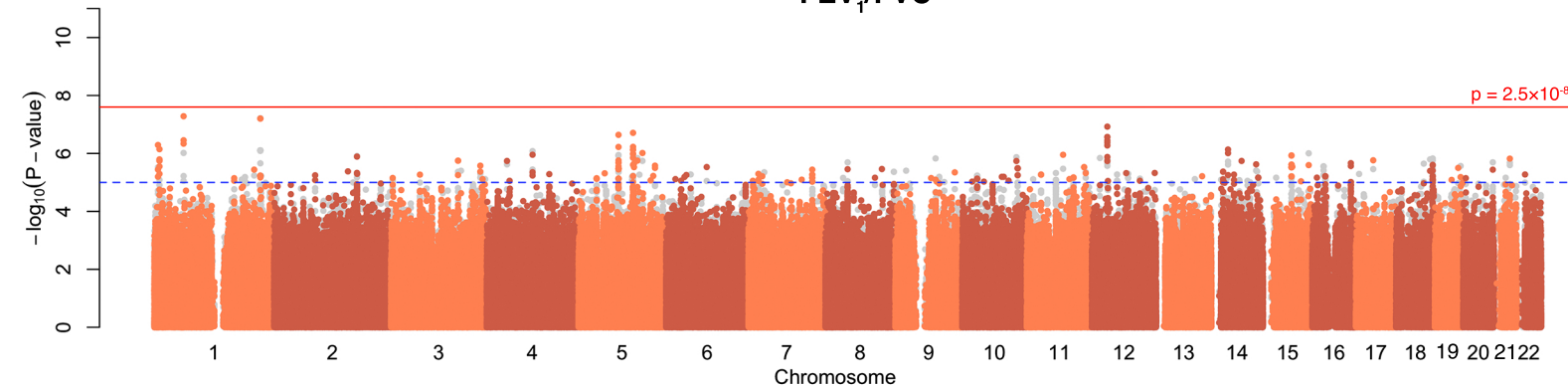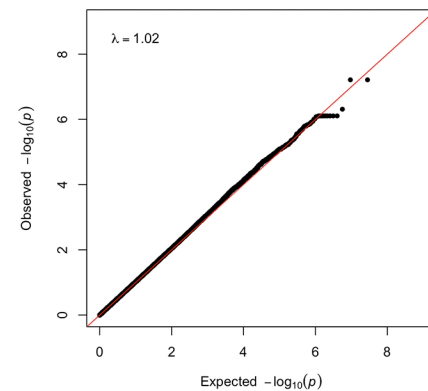

Supplement: S5 Fig — GWAS Manhattan plots for A) FEV1 and B) FEV1/FVC ratio, without adjustment for asthma status. The horizontal red line indicates genome-wide significance (p ≤ 2.5x10^−8^). The dotted horizontal blue line indicates p = 1x10^−5^. Variants colored in grey are the GWAS results with asthma adjustment. FEV1, forced expiratory volume in one second; FEV1/FVC, ratio of FEV1 to forced vital capacity. (PDF) [file pgen.1010594.s005.pdf]

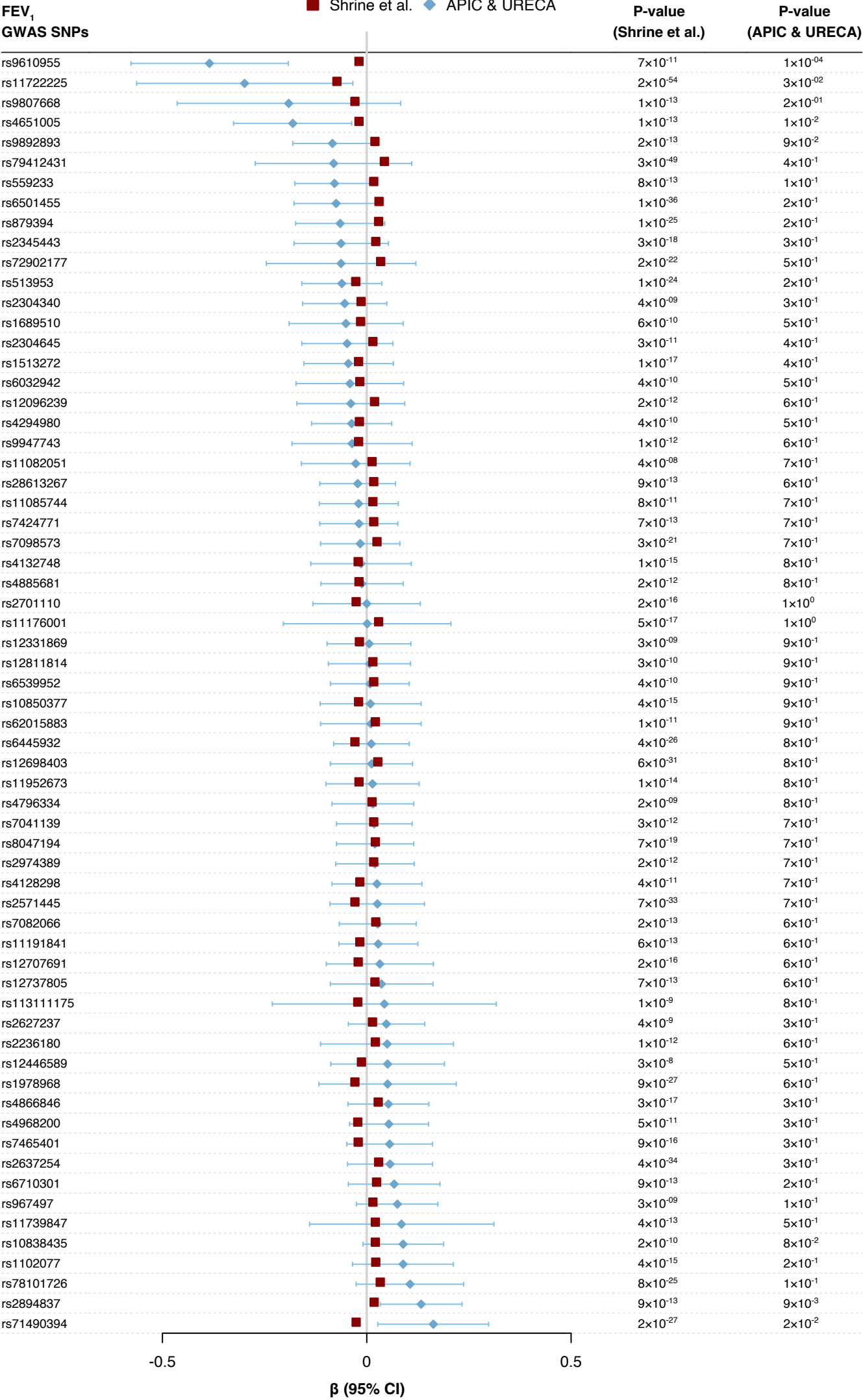

Supplement: S6 Fig — Association statistics for previously identified FEV1 GWAS SNPs [23]. 64 out of 70 previously identified SNPs were genotyped in APIC & URECA. GWAS, genome-wide association study; SNP, single nucleotide polymorphism; APIC, Asthma Phenotypes in the Inner City study; URECA, Urban Environment and Childhood Asthma study. FEV1, forced expiratory volume in one second. (PDF) [file pgen.1010594.s006.pdf]

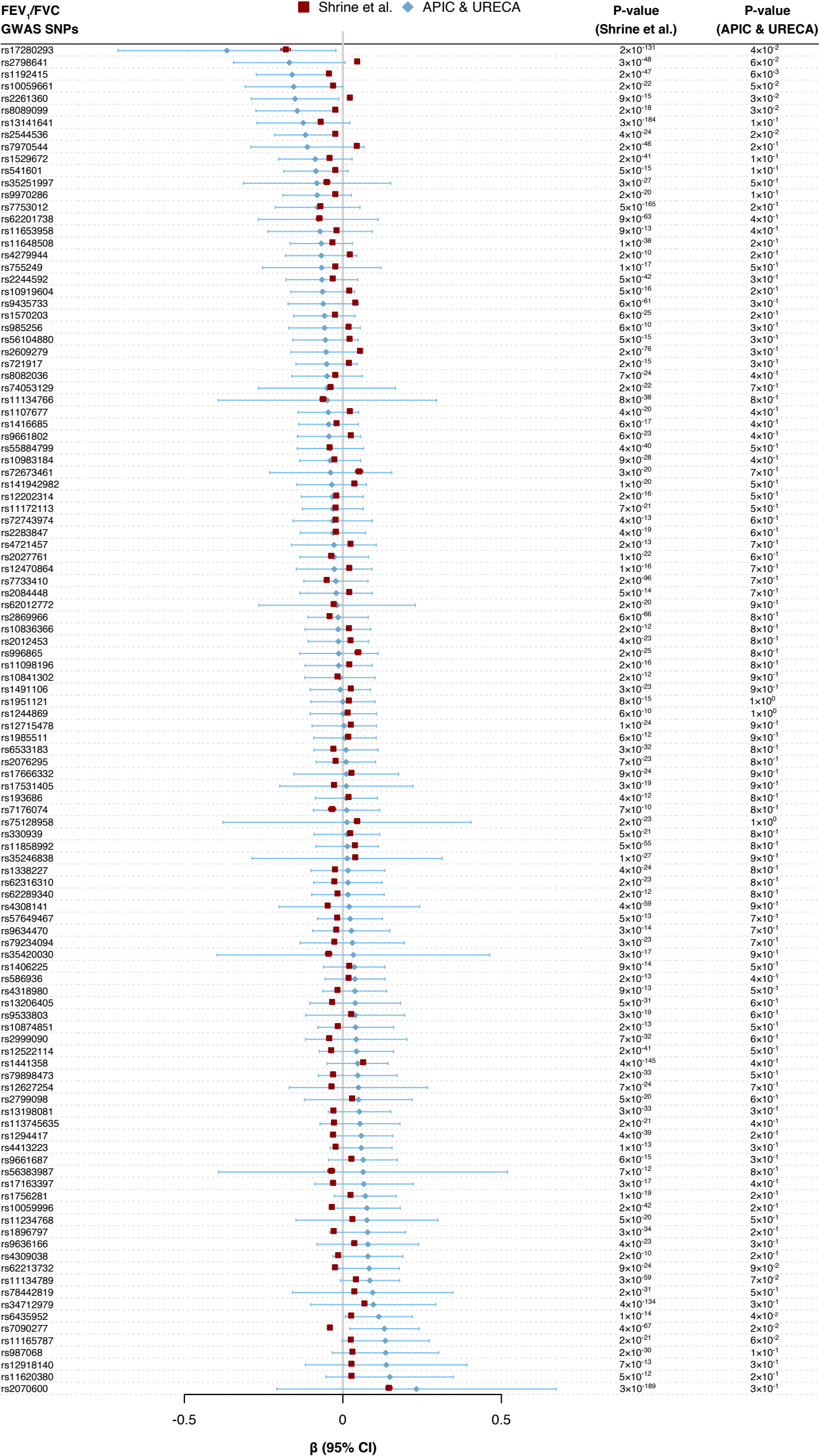

Supplement: S7 Fig — Association statistics for previously identified FEV1/FVC GWAS SNPs [23]. 112 out of 117 previously identified SNPs were genotyped in APIC & URECA. GWAS, genome-wide association study; SNP, single nucleotide polymorphism; APIC, Asthma Phenotypes in the Inner City study; URECA, Urban Environment and Childhood Asthma study. FEV1, forced expiratory volume in one second; FVC, forced vital capacity. (PDF) [file pgen.1010594.s007.pdf]

# NicAlert Results by Study

APIC

URECA

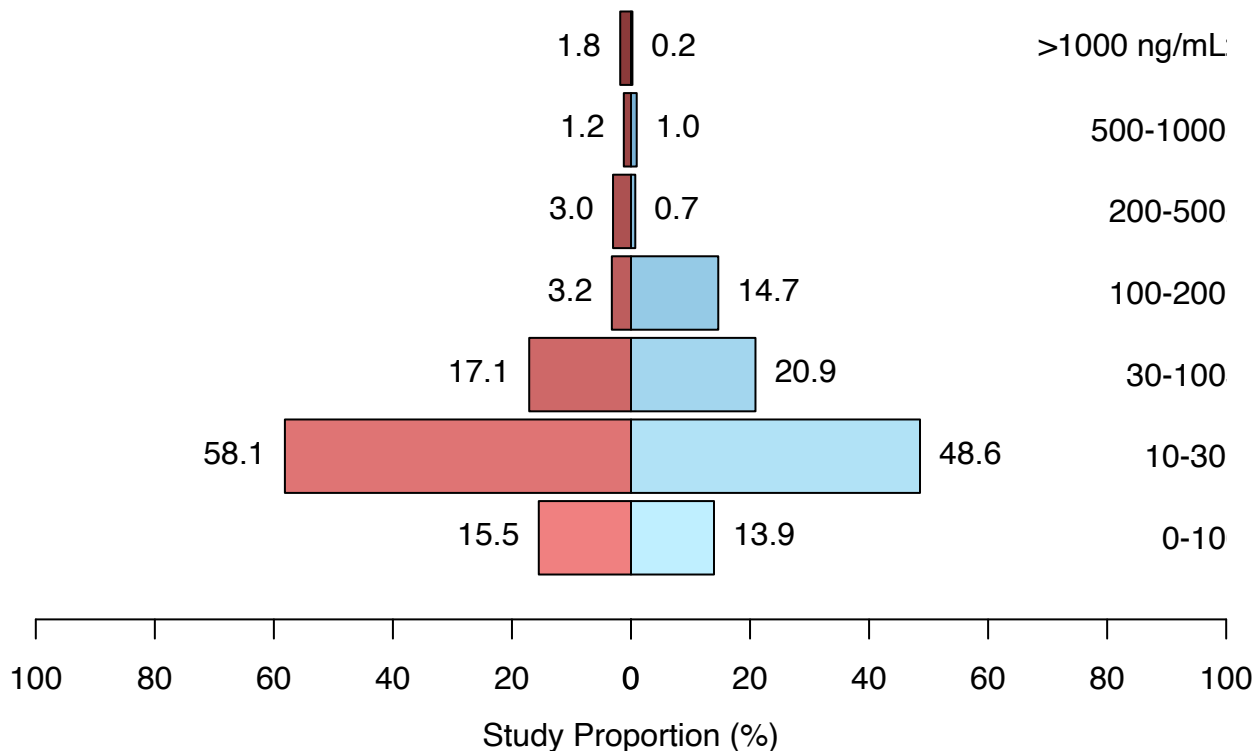

Supplement: S8 Fig — Distribution of urine cotinine levels, as measured using NicAlert immunochromatographic assays, which report results on a scale of 0–6 according to the labeled concentration ranges. Proportions were calculated relative to the number of samples with available NicAlert results. APIC, Asthma Phenotypes in the Inner City study; URECA, Urban Environment and Childhood Asthma study. (PDF) [file pgen.1010594.s008.pdf]

## NECs

## PBMCs

**A**

Low cotinine  
≤30ng/ml

High cotinine  
>30ng/ml

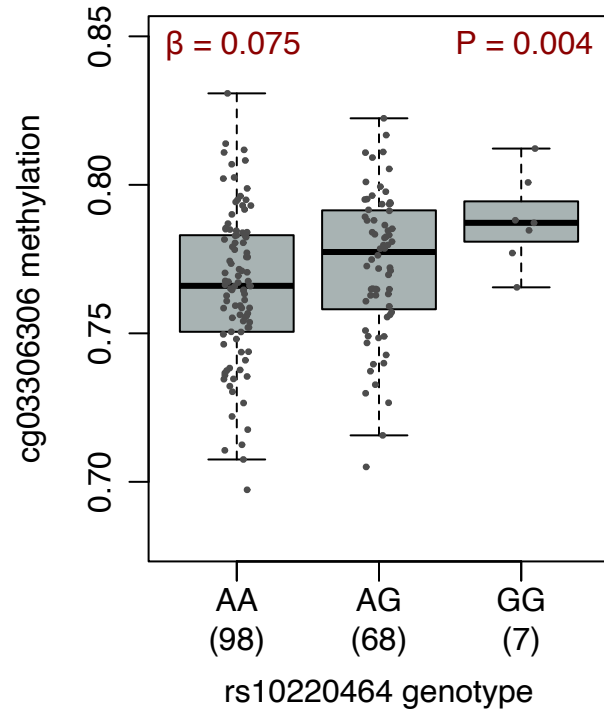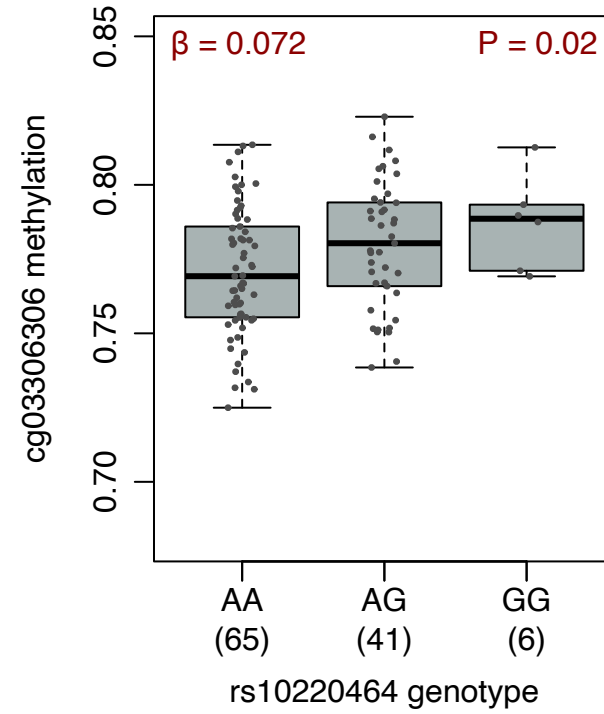

**B**

Low cotinine  
≤30ng/ml

High cotinine  
>30ng/ml

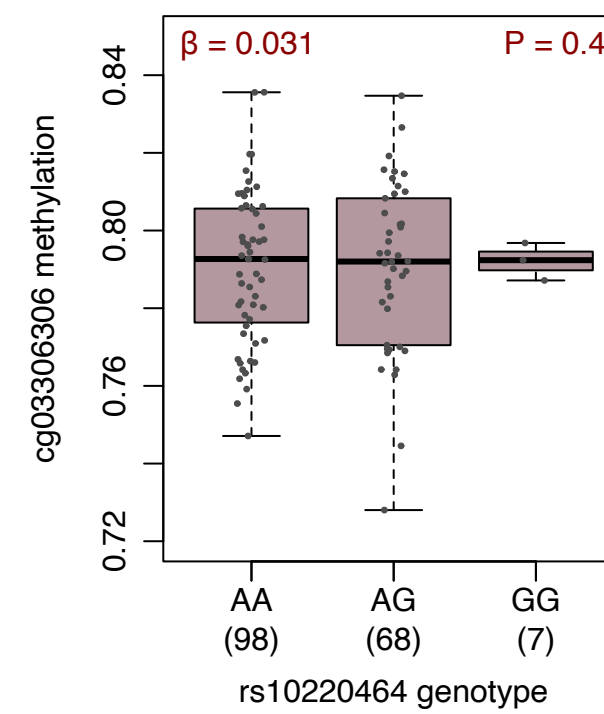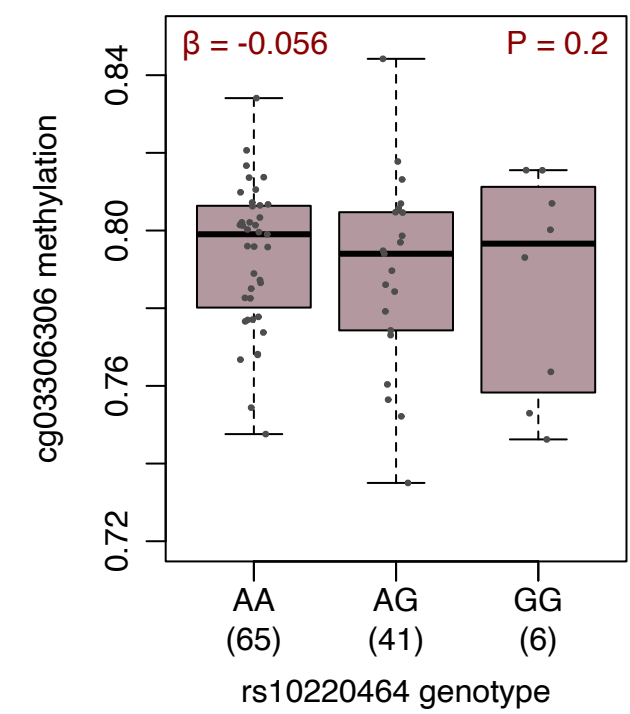

**C**

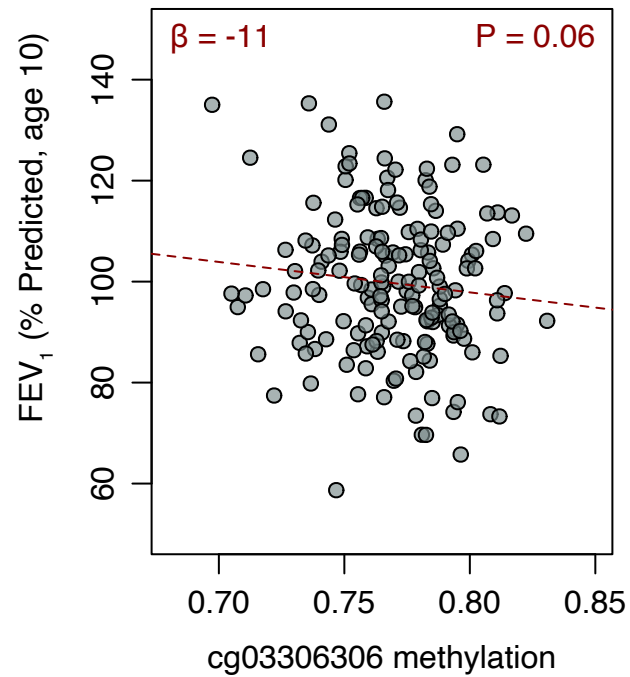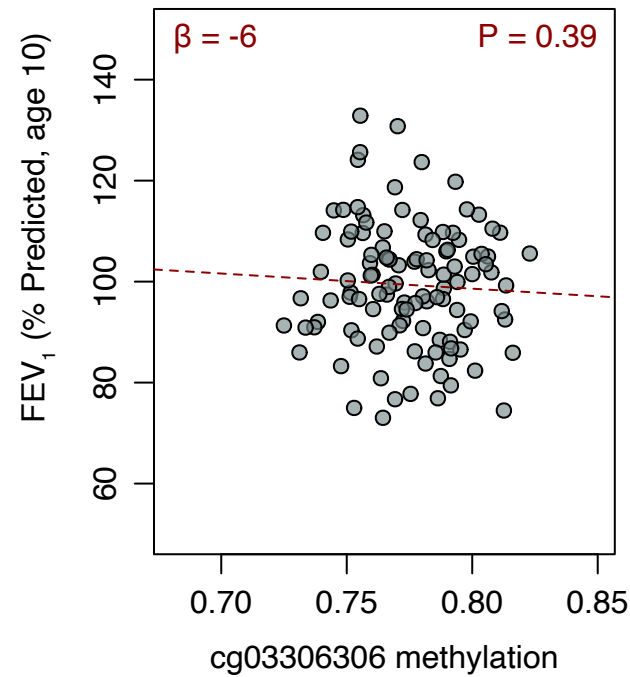

**D**

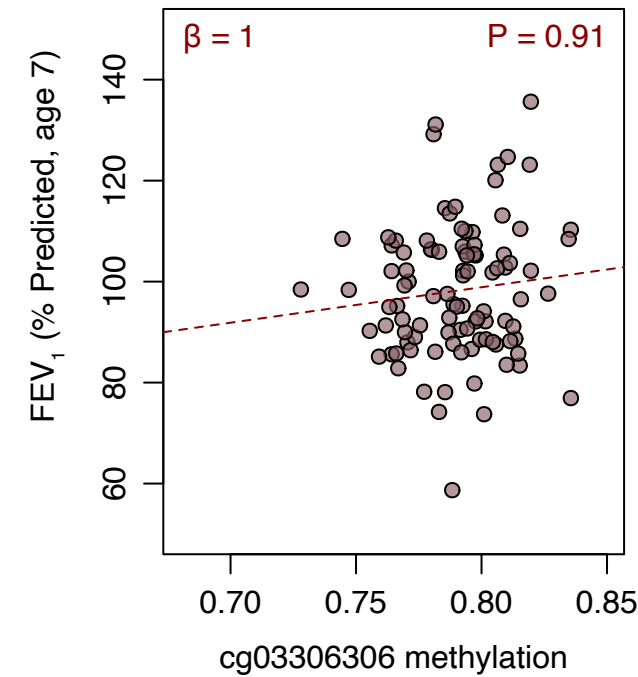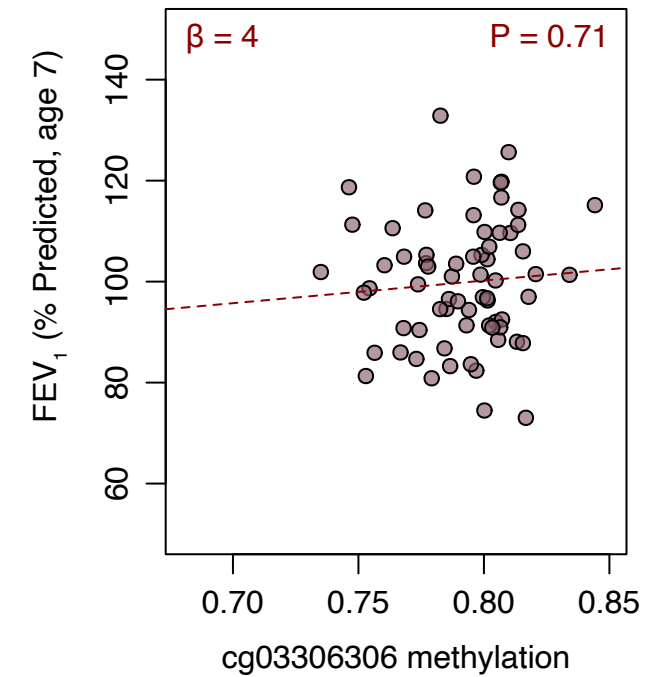

Supplement: S9 Fig — DNA methylation levels at cg03306306 are shown by rs10220464 genotype in URECA participants with low and high smoking exposures in (A) NECs at age 11 and (B) PBMCs at age 7. FEV1 (% predicted) are also shown by cg03306306 DNA methylation levels in URECA participants with low and high smoking exposures in (C) NECs at age 11 and (D) PBMCs at age 7. NECs, nasal epithelial cells; PBMCs, peripheral blood mononuclear cells; FEV1, forced expiratory volume in one second; URECA, Urban Environment and Childhood Asthma study. (PDF) [file pgen.1010594.s009.pdf]

**Low cotinine  
 $\leq 30\text{ng/ml}$**

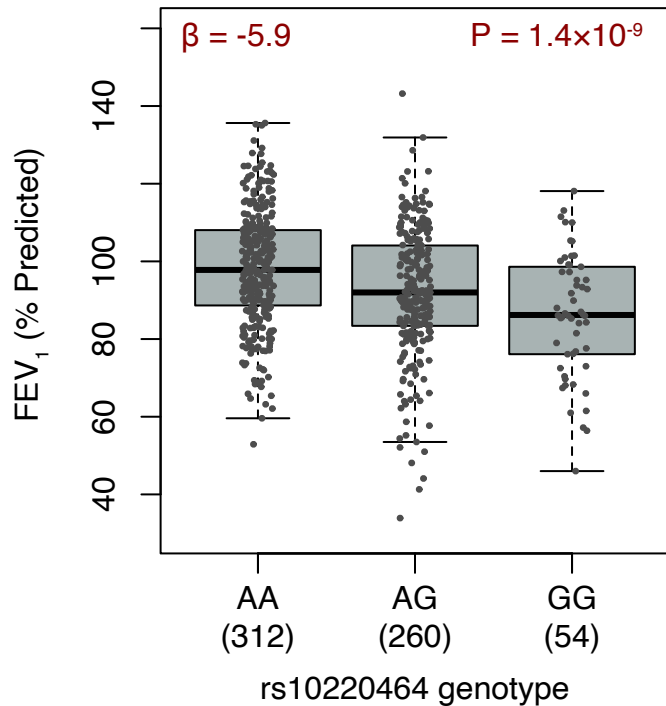

**High cotinine  
 $>30\text{ng/ml}$**

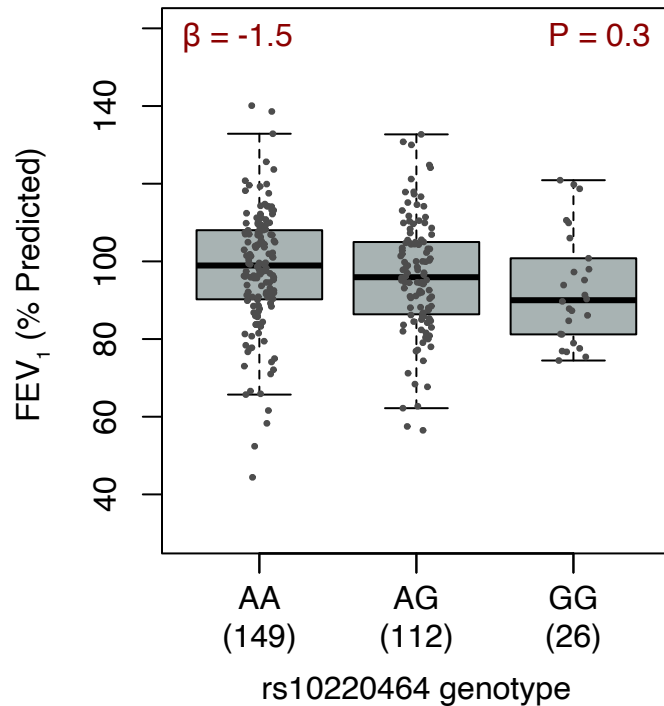

Supplement: S10 Fig — FEV1 (% predicted) are shown by rs10220464 genotype in APIC & URECA participants with low and high smoking exposures according to urine cotinine levels. FEV1, forced expiratory volume in one second; APIC, Asthma Phenotypes in the Inner City study; URECA, Urban Environment and Childhood Asthma study. (PDF) [file pgen.1010594.s010.pdf]

**A**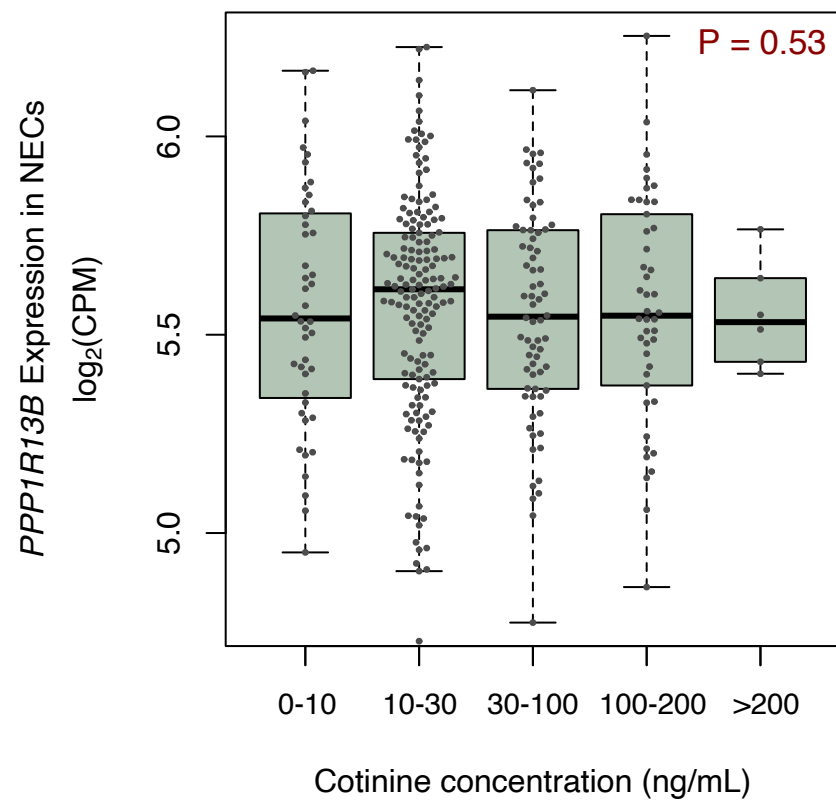**B**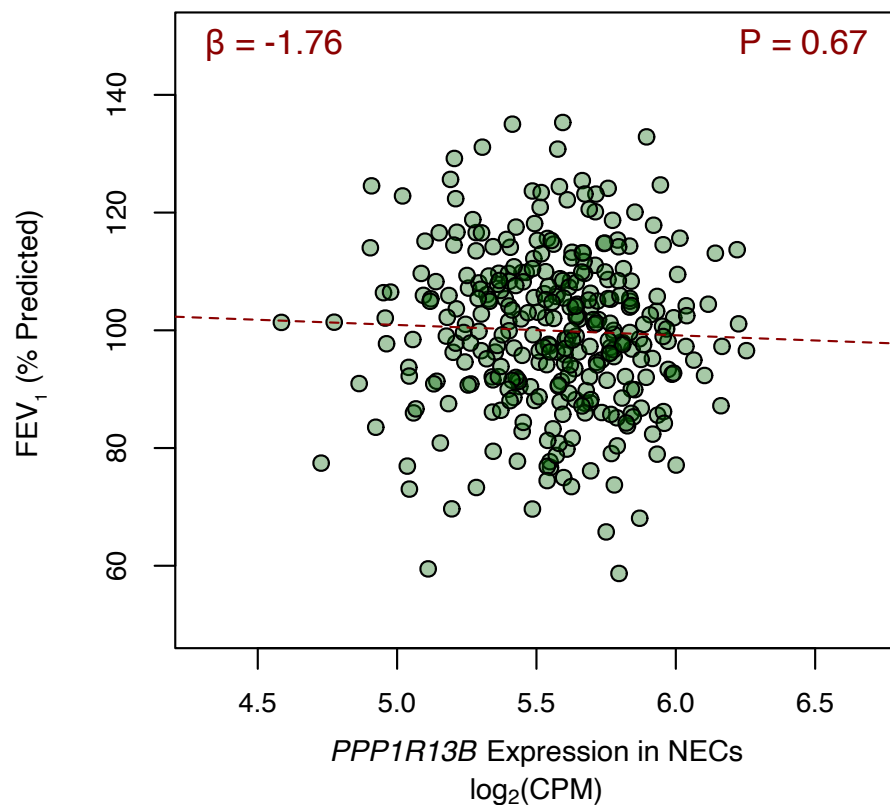

Supplement: S11 Fig — PPP1R13B expression in NECs at age 11 was not associated with smoking exposure at age 10 (A) nor with FEV1 (% predicted) at age 10 (B) in URECA. NECs, nasal epithelial cells; FEV1, forced expiratory volume in one second; URECA, Urban Environment and Childhood Asthma. (PDF) [file pgen.1010594.s011.pdf]

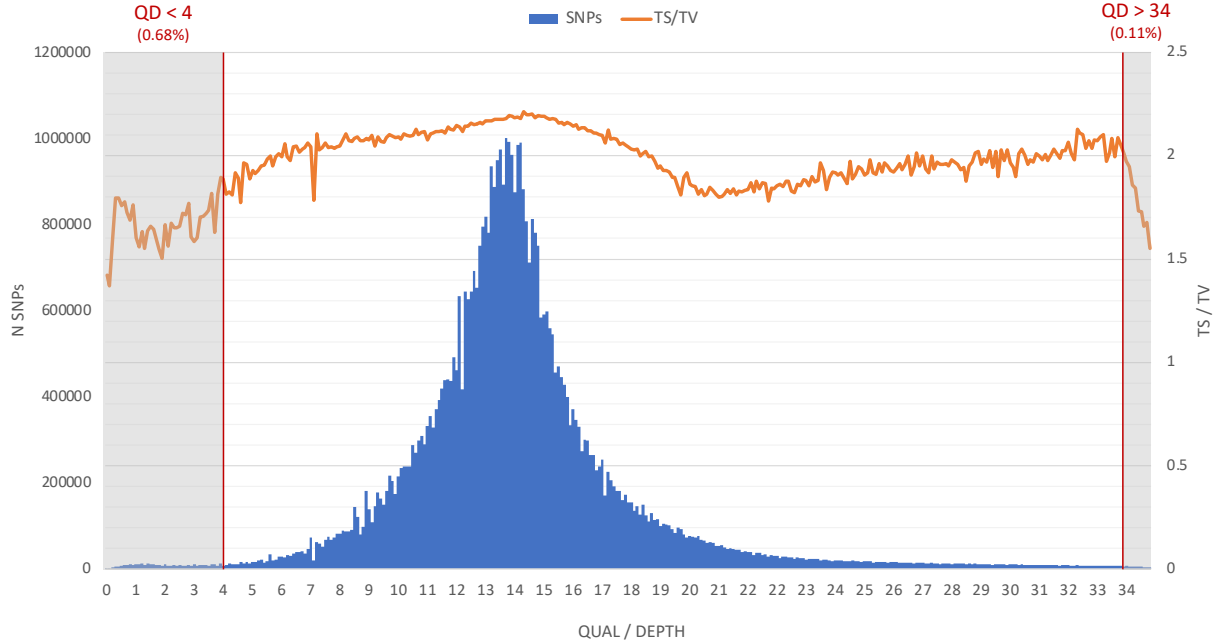

Supplement: S13 Fig — The transition/transversion ratio (TS/TV) is plotted against the variant call quality/depth metric (QD) across all WGS SNP calls in APIC & URECA. Sites with QD less than 4 or greater than 34 were removed from consideration in this study. SNPs, single nucleotide polymorphisms; WGS, whole-genome sequencing; APIC, Asthma Phenotypes in the Inner City study; URECA, Urban Environment and Childhood Asthma study. (PDF) [file pgen.1010594.s013.pdf]

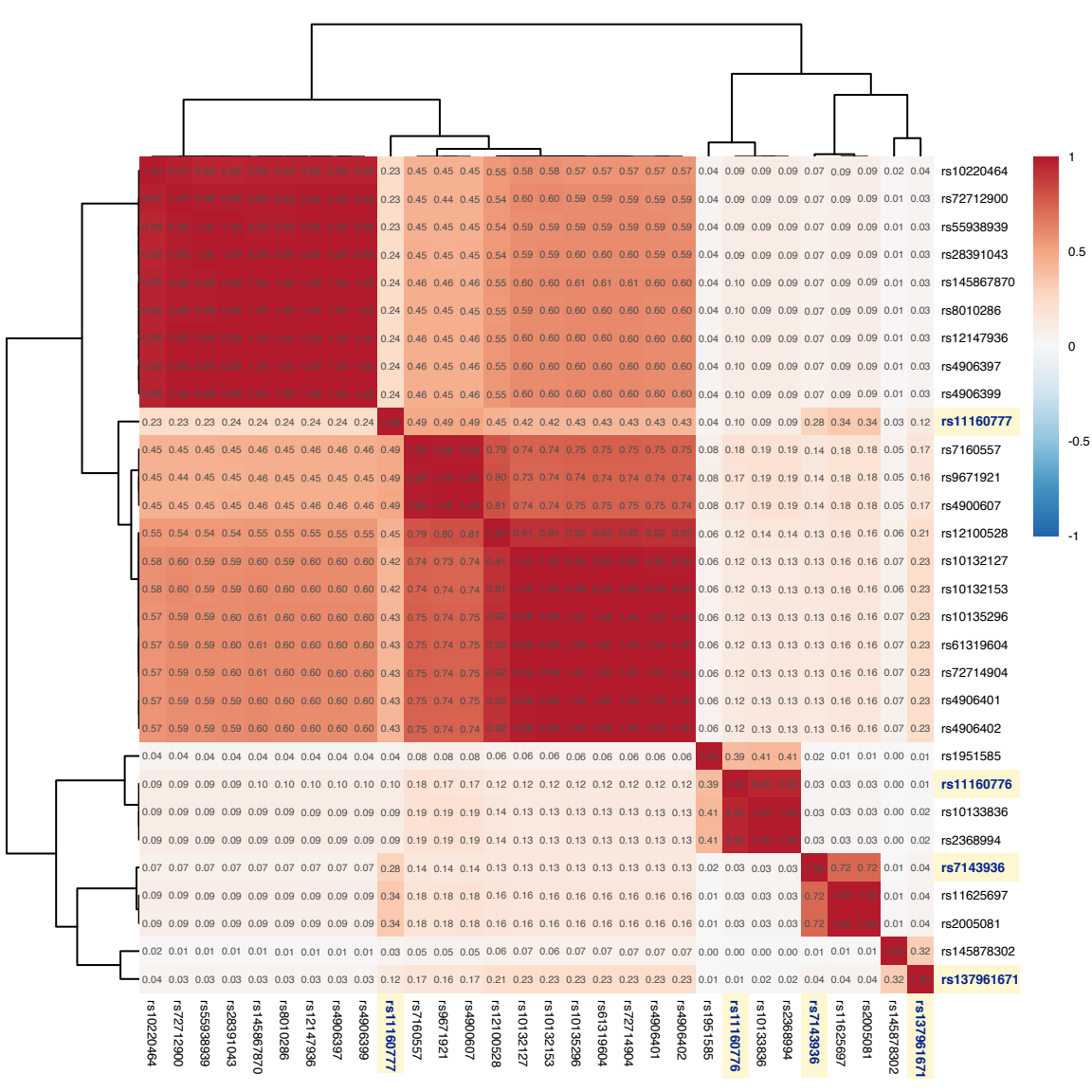

Supplement: S14 Fig — Instrumental variables were chosen from a set of candidate SNPs that were at least nominally associated with cg03306306 methylation with p<0.15. The correlation values between these SNPs are shown, clustered using Ward’s method. The four SNPs used for the instrument are highlighted. URECA, Urban Environment and Childhood Asthma. (PDF) [file pgen.1010594.s014.pdf]

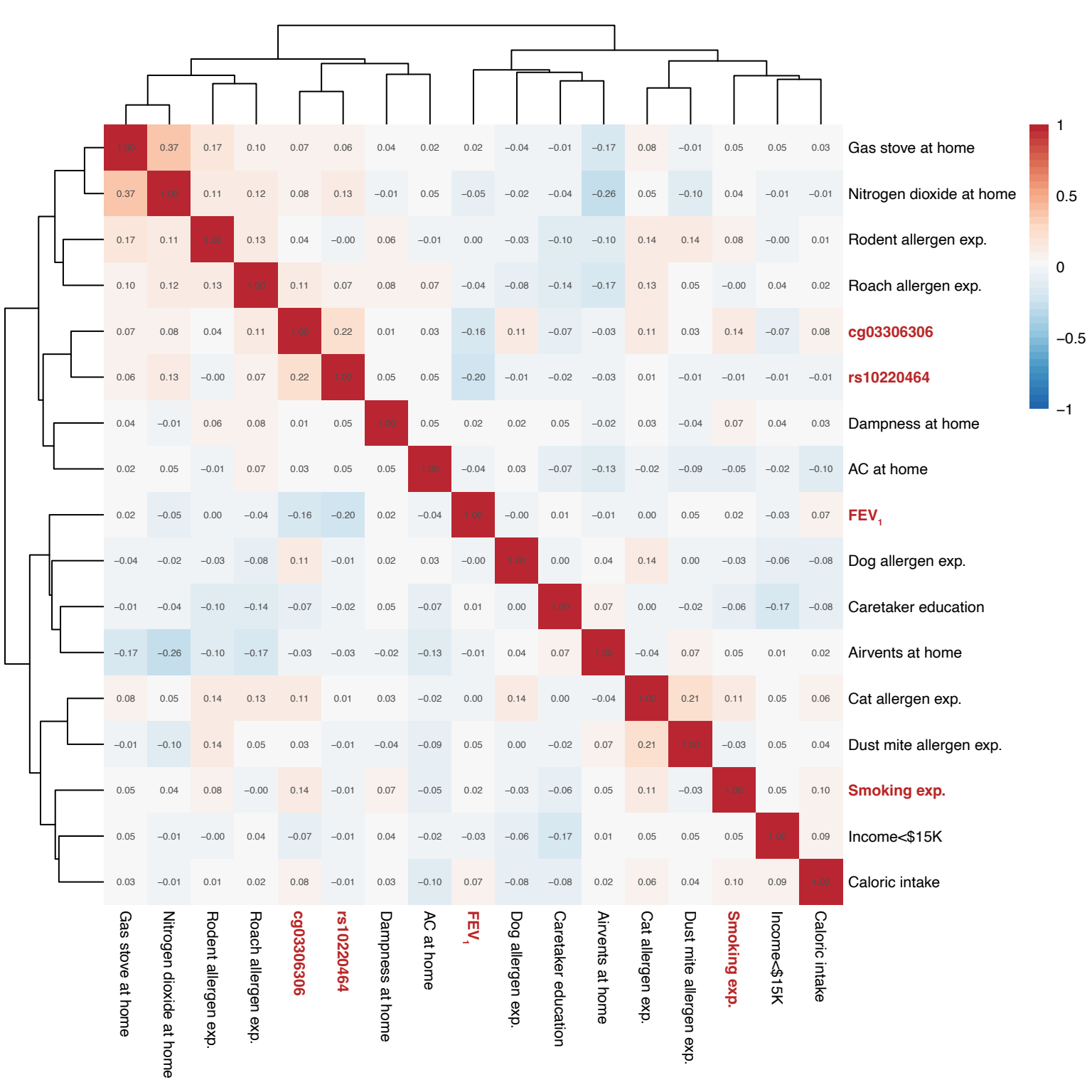

Supplement: S15 Fig — The correlations are shown between FEV1 (% predicted), smoking exposure (NicAlert), the primary the lead FEV1 SNP rs10220464, DNA methylation at cg03306306, 11 environmental exposures, and 2 socioeconomic indicators, clustered using Ward’s method. APIC, Asthma Phenotypes in the Inner City study; exp., exposure; URECA, Urban Environment and Childhood Asthma. (PDF) [file pgen.1010594.s015.pdf]
