# Supplementary material for: Multi-omic association study identifies DNA methylation-mediated genotype and smoking exposure effects on lung function in children living in urban settings
Source: PLoS Genet. 2023 Jan 13;19(1):e1010594. doi: 10.1371/journal.pgen.1010594 (PMC9879483; doi:10.1371/journal.pgen.1010594)
Supplement: S1 Table — Variant call concordance between three pairs of replicate samples, by variant type and cohort allele frequency. SNPs, single nucleotide polymorphisms; MAF, minor allele frequency; InDels, insertions and deletions. (PDF) [file pgen.1010594.s016.pdf]

S1 Table. Post-QC sequencing call concordance between replicates.

Variant Type	**Variants** (mean)	**Call concordance**
**All Variants**	4,284,228	0.9985
**SNPs**	3,793,694	0.9997
Common SNPs (MAF > 0.05)	3,244,661	0.9997
Low Frequency SNPs (0.01 > MAF ≤ 0.05)	383,918	0.9995
Rare SNPs (MAF ≤0.01)	165,115	0.9995
**InDels**	490,534	0.9892
Common InDels (MAF > 0.05)	428,621	0.9896
Low Frequency InDels (0.01 > MAF ≤ 0.05)	39,572	0.9845
Rare InDels (MAF ≤0.01)	22,340	0.9911

Variant call concordance between three pairs of replicate samples, by variant type and cohort allele frequency. SNPs, single nucleotide polymorphisms; MAF, minor allele frequency; InDels, insertions and deletions.
