# Supplementary material for: Multi-omic association study identifies DNA methylation-mediated genotype and smoking exposure effects on lung function in children living in urban settings
Source: PLoS Genet. 2023 Jan 13;19(1):e1010594. doi: 10.1371/journal.pgen.1010594 (PMC9879483; doi:10.1371/journal.pgen.1010594)
Supplement: S2 Table — All variants in chr14q32.33 associated with FEV1 (% predicted) with p<1x10^-5^ (n = 82) in GWAS of 896 participants from APIC & URECA. N, number of genotyped individuals. MAF, minor allele frequency; 95% CI, 95% confidence interval; SE, standard error; P, P-value (Wald); FEV1, forced expiratory volume in one second; APIC, Asthma Phenotypes in the Inner City study; URECA, Urban Environment and Childhood Asthma study. (PDF) [file pgen.1010594.s017.pdf]

S2 Table. FEV_1-associated variants in chr14q32.33

Pos	rsID	N	Ref	Alt	MAF	Beta	SE	P	Type	Gene
**103936818**	**rs10220464**	**896**	**A**	**G**	**0.30**	**-0.311**	**0.052**	**2.42×10^{-9}**	**intronic**	***TDRD9***
103926863	rs11850186	896	G	A	0.29	-0.307	0.052	5.34×10^{-9}	intergenic	*ATP5MPL; TDRD9*
103934654	rs4906387	896	C	T	0.29	-0.305	0.052	6.20×10^{-9}	intronic	*TDRD9*
103935736	rs9285602	896	C	T	0.29	-0.305	0.052	6.20×10^{-9}	intronic	*TDRD9*
103937635	rs9324069	896	G	A	0.29	-0.305	0.052	6.20×10^{-9}	intronic	*TDRD9*
103942966	.	896	GA	G	0.29	-0.305	0.052	6.20×10^{-9}	intronic	*TDRD9*
103960917	rs4900604	896	G	A	0.29	-0.305	0.052	6.20×10^{-9}	intronic	*TDRD9*
103963565	rs28574832	896	G	T	0.29	-0.305	0.052	6.20×10^{-9}	intronic	*TDRD9*
103967867	rs4900605	896	G	T	0.29	-0.305	0.052	6.20×10^{-9}	intronic	*TDRD9*
103997188	rs72712900	896	G	A	0.29	-0.304	0.052	6.51×10^{-9}	intronic	*TDRD9*
103984806	rs11847797	896	G	A	0.29	-0.303	0.052	7.73×10^{-9}	intronic	*TDRD9*
103980079	rs58289480	896	A	G	0.29	-0.303	0.052	8.28×10^{-9}	intronic	*TDRD9*
103985095	rs4906396	896	A	C	0.29	-0.303	0.052	8.28×10^{-9}	intronic	*TDRD9*
103953683	rs72712884	896	C	G	0.29	-0.303	0.052	8.28×10^{-9}	intronic	*TDRD9*
104046654	rs143594477	896	C	CT	0.29	-0.302	0.052	9.12×10^{-9}	intronic	*TDRD9*
104028226	rs10137997	896	T	C	0.29	-0.301	0.052	9.73×10^{-9}	intronic	*TDRD9*
104034493	rs9944163	896	G	T	0.29	-0.301	0.052	9.73×10^{-9}	intronic	*TDRD9*
104069368	rs72714937	896	C	T	0.29	-0.302	0.052	9.80×10^{-9}	intergenic	*TDRD9; ASPG*
104059833	rs11160785	896	T	A	0.29	-0.300	0.052	1.03×10^{-8}	intergenic	*TDRD9; ASPG*
103934222	rs28461376	896	A	G	0.29	-0.301	0.052	1.06×10^{-8}	intronic	*TDRD9*
103963484	.	896	TC	T	0.29	-0.301	0.052	1.06×10^{-8}	intronic	*TDRD9*
104037291	rs4906408	896	G	T	0.29	-0.299	0.052	1.14×10^{-8}	intronic	*TDRD9*
104030861	rs10135338	896	G	A	0.29	-0.299	0.052	1.18×10^{-8}	intronic	*TDRD9*
104035861	rs10134394	896	G	A	0.29	-0.299	0.052	1.18×10^{-8}	intronic	*TDRD9*
104036696	rs11160780	896	G	A	0.29	-0.299	0.052	1.18×10^{-8}	intronic	*TDRD9*
104037342	rs4900608	896	G	A	0.29	-0.299	0.052	1.18×10^{-8}	intronic	*TDRD9*
104038178	rs4906409	896	T	C	0.29	-0.299	0.052	1.18×10^{-8}	intronic	*TDRD9*
104039412	rs10144682	896	G	A	0.29	-0.299	0.052	1.18×10^{-8}	intronic	*TDRD9*
104012687	rs11851441	896	A	G	0.29	-0.299	0.052	1.23×10^{-8}	intronic	*TDRD9*
104031206	rs10135507	896	C	T	0.29	-0.298	0.052	1.25×10^{-8}	exonic	*TDRD9*
103998027	rs55938939	896	G	A	0.29	-0.299	0.052	1.26×10^{-8}	intronic	*TDRD9*
104048481	rs61244535	896	C	G	0.29	-0.299	0.052	1.29×10^{-8}	intronic	*TDRD9*
104051127	rs28644198	896	C	A	0.29	-0.299	0.052	1.29×10^{-8}	intronic	*TDRD9*
104054541	rs28377615	896	G	A	0.29	-0.299	0.052	1.29×10^{-8}	intergenic	*TDRD9; ASPG*
103997438	rs4906397	896	C	T	0.29	-0.298	0.052	1.31×10^{-8}	intronic	*TDRD9*
103999081	rs4906399	896	C	T	0.29	-0.298	0.052	1.31×10^{-8}	intronic	*TDRD9*
104005628	rs12147936	896	C	T	0.29	-0.298	0.052	1.31×10^{-8}	intronic	*TDRD9*
104010259	rs145867870	896	C	CT	0.29	-0.298	0.052	1.31×10^{-8}	intronic	*TDRD9*
104013496	rs11851723	896	C	T	0.29	-0.298	0.052	1.31×10^{-8}	intronic	*TDRD9*
104014746	rs11160779	896	T	C	0.29	-0.298	0.052	1.31×10^{-8}	exonic	*TDRD9*
104016537	rs72714909	896	C	A	0.29	-0.298	0.052	1.31×10^{-8}	intronic	*TDRD9*
103986068	rs10143030	896	C	G	0.29	-0.300	0.052	1.41×10^{-8}	intronic	*TDRD9*
103986303	rs10143389	896	G	A	0.29	-0.300	0.052	1.41×10^{-8}	exonic	*TDRD9*
103986825	rs61248168	896	T	A	0.29	-0.300	0.052	1.41×10^{-8}	intronic	*TDRD9*
103990858	rs1957518	896	G	A	0.29	-0.300	0.052	1.41×10^{-8}	intronic	*TDRD9*
104072329	rs111576189	896	G	A	0.29	-0.298	0.052	1.54×10^{-8}	intergenic	*TDRD9; ASPG*
104011541	rs28522352	896	T	A	0.29	-0.296	0.052	1.79×10^{-8}	intronic	*TDRD9*
104003975	rs8010286	896	T	C	0.29	-0.293	0.052	2.06×10^{-8}	intronic	*TDRD9*
104004349	rs28391043	896	G	A	0.29	-0.294	0.052	2.22×10^{-8}	intronic	*TDRD9*
104027784	rs28725314	896	C	T	0.29	-0.290	0.052	2.96×10^{-8}	intronic	*TDRD9*
103970594	rs11851097	896	C	T	0.36	-0.267	0.048	3.28×10^{-8}	exonic	*TDRD9*

Pos	rsID	N	Ref	Alt	MAF	Beta	SE	P	Type	Gene
103987132	rs200735321	880	A	G	0.26	-0.311	0.057	6.98 x10^{-8}	intronic	*TDRD9*
104067149	rs10782497	896	T	C	0.39	-0.265	0.049	1.08 x10^{-7}	intergenic	*TDRD9; ASPG*
104019632	rs74089113	896	G	C	0.44	-0.246	0.046	1.25 x10^{-7}	intronic	*TDRD9*
104071795	rs35775205	896	A	G	0.37	-0.264	0.050	1.72 x10^{-7}	intergenic	*TDRD9; ASPG*
104038358	rs877009	896	T	C	0.43	-0.240	0.046	3.06 x10^{-7}	intronic	*TDRD9*
104007407	rs12100528	896	G	A	0.43	-0.240	0.047	3.32 x10^{-7}	intronic	*TDRD9*
104016337	rs7158223	896	G	T	0.42	-0.238	0.046	3.83 x10^{-7}	intronic	*TDRD9*
103947548	rs7144813	896	G	C	0.35	-0.244	0.049	8.48 x10^{-7}	intronic	*TDRD9*
103937851	rs10141985	896	G	A	0.34	-0.242	0.050	1.53 x10^{-6}	intronic	*TDRD9*
103939314	rs7148877	896	A	G	0.34	-0.241	0.050	1.73 x10^{-6}	intronic	*TDRD9*
103945800	rs12147460	896	T	G	0.34	-0.241	0.050	1.73 x10^{-6}	intronic	*TDRD9*
104032500	rs9944058	896	C	T	0.42	-0.235	0.049	2.22 x10^{-6}	intronic	*TDRD9*
103981884	rs4906395	896	T	C	0.42	-0.220	0.047	2.71 x10^{-6}	intronic	*TDRD9*
103958246	rs10149734	896	A	C	0.41	-0.221	0.047	3.04 x10^{-6}	intronic	*TDRD9*
103962412	rs72712889	896	T	C	0.41	-0.221	0.047	3.04 x10^{-6}	intronic	*TDRD9*
103967964	rs4906392	896	T	C	0.41	-0.221	0.047	3.04 x10^{-6}	intronic	*TDRD9*
103995217	rs10132153	896	C	A	0.41	-0.220	0.047	3.16 x10^{-6}	intronic	*TDRD9*
104036896	rs7156122	896	G	A	0.41	-0.217	0.046	3.28 x10^{-6}	intronic	*TDRD9*
104014863	rs397949797	896	A	AT	0.41	-0.216	0.046	3.75 x10^{-6}	intronic	*TDRD9*
103979872	rs8022833	896	A	G	0.41	-0.219	0.047	3.87 x10^{-6}	intronic	*TDRD9*
103981060	rs1957517	896	A	G	0.41	-0.219	0.047	3.87 x10^{-6}	intronic	*TDRD9*
103995157	rs10132127	896	C	G	0.41	-0.218	0.047	4.02 x10^{-6}	intronic	*TDRD9*
104004783	rs9671921	896	A	G	0.49	-0.214	0.046	4.98 x10^{-6}	intronic	*TDRD9*
104008697	rs4906401	896	A	G	0.41	-0.215	0.047	5.23 x10^{-6}	intronic	*TDRD9*
104001342	rs61319604	896	C	T	0.41	-0.215	0.047	5.35 x10^{-6}	intronic	*TDRD9*
104002208	rs72714904	896	T	C	0.41	-0.215	0.047	5.35 x10^{-6}	intronic	*TDRD9*
104003206	rs10135296	896	G	T	0.41	-0.215	0.047	5.35 x10^{-6}	intronic	*TDRD9*
104006916	rs4900607	896	A	G	0.49	-0.212	0.046	5.90 x10^{-6}	intronic	*TDRD9*
104010388	rs4906402	896	G	A	0.41	-0.213	0.047	6.22 x10^{-6}	intronic	*TDRD9*
104000834	rs7160557	896	G	A	0.48	-0.207	0.046	8.97 x10^{-6}	intronic	*TDRD9*
104078948	rs4906415	896	G	A	0.19	-0.268	0.060	9.00 x10^{-6}	intergenic	*TDRD9; ASPG*

All variants in chr14q32.33 associated with FEV_1 (% predicted) with $p<1x10^{-5}$ (n=82) in GWAS of 896 participants from APIC & URECA. N, number of genotyped individuals. MAF, minor allele frequency; 95% CI, 95% confidence interval; SE, standard error; P, P-value (Wald); FEV_1, forced expiratory volume in one second; APIC, Asthma Phenotypes in the Inner City study; URECA, Urban Environment and Childhood Asthma study.
