# Supplementary material for: Multi-omic association study identifies DNA methylation-mediated genotype and smoking exposure effects on lung function in children living in urban settings
Source: PLoS Genet. 2023 Jan 13;19(1):e1010594. doi: 10.1371/journal.pgen.1010594 (PMC9879483; doi:10.1371/journal.pgen.1010594)
Supplement: S3 Table — All CpG sites where DNA methylation levels in NECs at age 11 in URECA were associated with rs10220464 at FDR<0.05 are shown with their corresponding associations with FEV1. The FDR-adjusted P-values (FDR Q) correspond to a 5% false-discovery rate. FDR, false discovery rate; 95% CI, 95% confidence interval; FEV1, forced expiratory volume in one second; URECA, Urban Environment and Childhood Asthma study. (PDF) [file pgen.1010594.s018.pdf]

S3 Table. MeQTL analysis results and associations with FEV_1

CpG Site	Position (strand), hg38 (chr14)	rs10220464			FEV_1	
		Beta [95% CI]	P	FDR Q	Beta [95% CI]	P
cg21567958	103909862 (-)	0.10 [0.05, 0.14]	1.79×10^{-5}	0.01	-1.5 [-8.8, 5.7]	0.68
cg12183467	103885907 (-)	0.10 [0.05, 0.14]	1.31×10^{-4}	0.04	5.3 [-1.5, 12.1]	0.13
cg16820107	103970549 (-)	0.08 [0.04, 0.12]	1.62×10^{-4}	0.04	-5.4 [-13.4, 2.5]	0.18
**cg03306306**	**104001397 (+)**	**0.07 [0.03, 0.11]**	**2.28×10^{-4}**	**0.04**	**-11.5 [-20.3, -2.7]**	**0.01**
cg17298714	103938355 (+)	0.14 [0.07, 0.21]	2.36×10^{-4}	0.04	-4.1 [-8.7, 0.4]	0.07

All CpG sites where DNA methylation levels in NECs at age 11 in URECA were associated with rs10220464 at FDR<0.05 are shown with their corresponding associations with FEV_1. The FDR-adjusted P-values (FDR Q) correspond to a 5% false-discovery rate. FDR, false discovery rate; 95% CI, 95% confidence interval; FEV_1, forced expiratory volume in one second; URECA, Urban Environment and Childhood Asthma study.
