# Supplementary material for: Multi-omic association study identifies DNA methylation-mediated genotype and smoking exposure effects on lung function in children living in urban settings
Source: PLoS Genet. 2023 Jan 13;19(1):e1010594. doi: 10.1371/journal.pgen.1010594 (PMC9879483; doi:10.1371/journal.pgen.1010594)
Supplement: S4 Table — Results of eQTL analyses in NECs and PBMCs with rs10220464 for all genes within 1 Mb in URECA. Gene expression was measured in counts per million mapped reads. The FDR-adjusted P-values (FDR Q) correspond to a 5% false-discovery rate. FDR, false discovery rate; 95% CI, 95% confidence interval; NECs, nasal epithelial cells; PBMCs, peripheral blood mononuclear cells; URECA, Urban Environment and Childhood Asthma study. (PDF) [file pgen.1010594.s019.pdf]

S4 Table. **rs10220464 eQTL analysis results**

Gene	NECs			PBMCs	
	Beta [95% CI]	P	FDR Q	Beta [95% CI]	P
PPP1R13B	**0.12 [0.06, 0.17]**	**1.26 x10^{-5}**	**2.77 x10^{-4}**	0.01 [-0.08, 0.09]	0.87
BAG5	-0.06 [-0.02, -0.10]	0.0054	0.059	0.03 [-0.03, 0.08]	0.31
ASPG	0.12 [-0.02, 0.26]	0.089	0.48	NOT EXPRESSED	
CKB	-0.04 [0.01, -0.09]	0.10	0.48	0.13 [-0.03, 0.30]	0.12
KLC1	-0.04 [0.01, -0.08]	0.12	0.48	0.02 [-0.04, 0.07]	0.53
TNFAIP2	-0.05 [0.02, -0.12]	0.15	0.48	-0.11 [0.06, -0.28]	0.22
MARK3	-0.03 [0.01, -0.07]	0.15	0.48	-0.02 [0.02, -0.06]	0.40
C14orf2	0.01 [-0.01, 0.03]	0.23	0.61	-0.03 [0.03, -0.09]	0.35
XRCC3	0.08 [-0.06, 0.22]	0.25	0.61	0.00 [0.06, -0.07]	0.92
ZFYVE21	0.02 [-0.01, 0.04]	0.28	0.61	-0.02 [0.03, -0.07]	0.49
ADSSL1	0.04 [-0.04, 0.11]	0.33	0.61	0.04 [-0.09, 0.17]	0.54
PLD4	0.07 [-0.07, 0.20]	0.33	0.61	0.07 [-0.12, 0.26]	0.47
SIVA1	-0.01 [0.01, -0.03]	0.36	0.61	0.01 [-0.06, 0.09]	0.77
EXOC3L4	-0.08 [0.14, -0.29]	0.47	0.69	NOT EXPRESSED	
TRMT61A	-0.02 [0.04, -0.09]	0.48	0.69	-0.04 [0.04, -0.12]	0.32
CEP170B	-0.02 [0.05, -0.09]	0.51	0.69	0.02 [-0.11, 0.14]	0.78
APOPT1	-0.02 [0.04, -0.08]	0.56	0.69	-0.03 [0.03, -0.09]	0.37
EIF5	-0.01 [0.02, -0.04]	0.60	0.69	-0.03 [0.05, -0.12]	0.44
TDRD9	-0.07 [0.21, -0.36]	0.60	0.69	0.01 [-0.23, 0.26]	0.91
ZBTB42	-0.02 [0.05, -0.08]	0.63	0.69	-0.09 [0.01, -0.19]	0.089
INF2	-0.01 [0.08, -0.10]	0.84	0.85	0.04 [-0.06, 0.13]	0.45
AKT1	0.00 [0.03, -0.03]	0.85	0.85	-0.02 [0.04, -0.08]	0.57
AL049840.1	NOT EXPRESSED			0.00 [0.08, -0.09]	0.93
AL049840.4	NOT EXPRESSED			0.01 [-0.05, 0.08]	0.65
AL049840.5	NOT EXPRESSED			0.02 [-0.08, 0.13]	0.68
AL133367.1	NOT EXPRESSED			0.09 [-0.02, 0.20]	0.12
LINC00638	NOT EXPRESSED			0.03 [-0.11, 0.18]	0.66

Results of eQTL analyses in NECs and PBMCs with rs10220464 for all genes within 1 Mb in URECA. Gene expression was measured in counts per million mapped reads. The FDR-adjusted P-values (FDR Q) correspond to a 5% false-discovery rate. FDR, false discovery rate; 95% CI, 95% confidence interval; NECs, nasal epithelial cells; PBMCs, peripheral blood mononuclear cells; URECA, Urban Environment and Childhood Asthma study.
