# Supplementary material for: Multi-omic association study identifies DNA methylation-mediated genotype and smoking exposure effects on lung function in children living in urban settings
Source: PLoS Genet. 2023 Jan 13;19(1):e1010594. doi: 10.1371/journal.pgen.1010594 (PMC9879483; doi:10.1371/journal.pgen.1010594)
Supplement: S5 Table — Bait and target fragments refer to mapped Hi-C restriction fragments on chr14 (hg38) for gene promoters and putative enhancers, respectively. FEV1 SNPs refer to number of FEV1-associated variants (p<1x10^-5^) within 1kb of target fragment. SNPs, single nucleotide polymorphisms; FEV1, forced expiratory volume in one second. (PDF) [file pgen.1010594.s020.pdf]

S5 Table. Chromatin interactions with FEV_1-associated SNPs

Promoter	Bait Fragment	Target Fragment	Strand	Distance	FEV_1 SNPs	CHiCAGO Score
PPP1R13B	**103845355-103845955**	**104003512-104004312**	**-**	**157557**	**4**	**9.38**
CDCA4	105020395-105021827	104010864-104011532	-	1008863	3	7.65
PPP1R13B	**103845355-103845955**	**104004616-104004922**	**-**	**158661**	**4**	**7.16**
ZFYVE21	103716777-103717467	103948470-103949743	+	231003	1	6.57
ZFYVE21	103716777-103717467	103933835-103934479	+	216368	2	6.49
C14orf79	104985639-104987255	103935625-103936182	+	1049457	3	6.10
MARK3	103385080-103385712	103937160-103938502	+	551448	4	5.89
ZFYVE21	103715624-103716777	103981359-103981909	+	264582	2	5.89
PLD4	104927266-104927937	103963380-103964018	+	963248	3	5.69
ZFYVE21	103715624-103716777	103933397-103933835	+	216620	2	5.62
ADSSL1	104720007-104724747	104017055-104017482	+	702525	2	5.52
APOPT1	103562490-103563614	103999031-103999343	+	435417	1	5.48
CKB	103521551-103522109	103997308-103998030	-	475199	3	5.26
MARK3	103385881-103386341	103958731-103959336	+	572390	1	5.19
PPP1R13B	**103845355-103845955**	**104036454-104037811**	**-**	**190499**	**7**	**5.00**

Bait and target fragments refer to mapped Hi-C restriction fragments on chr14 (hg38) for gene promoters and putative enhancers, respectively. FEV_1 SNPs refer to number of FEV_1-associated variants ($p<1x10^{-5}$) within 1kb of target fragment. SNPs, single nucleotide polymorphisms; FEV_1, forced expiratory volume in one second.
