# Supplementary material for: Multi-omic association study identifies DNA methylation-mediated genotype and smoking exposure effects on lung function in children living in urban settings
Source: PLoS Genet. 2023 Jan 13;19(1):e1010594. doi: 10.1371/journal.pgen.1010594 (PMC9879483; doi:10.1371/journal.pgen.1010594)
Supplement: S6 Table — URECA, Urban Environment and Childhood Asthma study; FEV1, forced expiratory volume in one second; FVC, forced vital capacity. (PDF) [file pgen.1010594.s021.pdf]

S6 Table. Age at used lung function measure in URECA

Age (years)	Count	
	FEV_1	FEV_1/FVC
10	382	372
9	26	30
8	10	11
7	8	5
6	6	10
5	9	10

URECA, Urban Environment and Childhood Asthma study; FEV_1, forced expiratory volume in one second; FVC, forced vital capacity.
