# Supplementary material for: Multi-omic association study identifies DNA methylation-mediated genotype and smoking exposure effects on lung function in children living in urban settings
Source: PLoS Genet. 2023 Jan 13;19(1):e1010594. doi: 10.1371/journal.pgen.1010594 (PMC9879483; doi:10.1371/journal.pgen.1010594)
Supplement: S7 Table — APIC, Asthma Phenotypes in the Inner City study; URECA, Urban Environment and Childhood Asthma study; WGS, whole-genome sequencing; NECs, nasal epithelial cells; PBMCs, peripheral blood mononuclear cells. (PDF) [file pgen.1010594.s022.pdf]

S7 Table. Study samples

Cohort	Sample	Cell Type	Age Collected	N
APIC	WGS	-	-	508
URECA	WGS	-	-	527
URECA	DNA Methylation	NECs	11	286
URECA	DNA Methylation	PBMCs	7	169
URECA	RNA-Seq	NECs	11	324
URECA	RNA-Seq	PBMCs	2	132

APIC, Asthma Phenotypes in the Inner City study; URECA, Urban Environment and Childhood Asthma study; WGS, whole-genome sequencing; NECs, nasal epithelial cells; PBMCs, peripheral blood mononuclear cells.
