# Supplementary material for: Multi-omic association study identifies DNA methylation-mediated genotype and smoking exposure effects on lung function in children living in urban settings
Source: PLoS Genet. 2023 Jan 13;19(1):e1010594. doi: 10.1371/journal.pgen.1010594 (PMC9879483; doi:10.1371/journal.pgen.1010594)
Supplement: S8 Table — Additional variables examined for potential confounding in mediation analyses for APIC & URECA. APIC, Asthma Phenotypes in the Inner City study; URECA, Urban Environment and Childhood Asthma study. (PDF) [file pgen.1010594.s023.pdf]

S8 Table. Additional phenotypic, socioeconomic, and environmental data

Variable	Description	Units	N
**Socioeconomic factors**			
Income<$15K	Family yearly income <$15,000	yes/no	1018
Caretaker education	Caretaker completed high school	yes/no	1031
**Environmental exposures**			
Gas stove at home	Gas stove in home	yes/no	982
Dampness at home	Water problems in home	yes/no	981
AC at home	AC unit in child's bedroom	yes/no	982
Airvents at home	Forced air for heat in home	yes/no	983
Rodent allergen exposure	Mus m 1 (mouse) bedroom allergen levels	log(μg/g)	847
Roach allergen exposure	Bla g 1 (German cockroach) bedroom allergen levels	log(μg/g)	850
Dust mite allergen exposure	Combined Der f 1 (*Dermatophagoides farina*) and Der p 1 (*Dermatophagoides pteronyssinus)* bedroom allergen levels	log(μg/g)	756
Dog allergen exposure	Can f 1 bedroom allergen levels	log(μg/g)	839
Cat allergen exposure	Fel d 1 bedroom allergen levels	log(μg/g)	839
NO_2 at home	Indoor nitrogen dioxide levels	log(ppb)	900
Caloric intake	Total caloric intake	log(calories/day)	787

Additional variables examined for potential confounding in mediation analyses for APIC & URECA. APIC, Asthma Phenotypes in the Inner City study; URECA, Urban Environment and Childhood Asthma study.
