# Supplementary material for: Multi-omic association study identifies DNA methylation-mediated genotype and smoking exposure effects on lung function in children living in urban settings
Source: PLoS Genet. 2023 Jan 13;19(1):e1010594. doi: 10.1371/journal.pgen.1010594 (PMC9879483; doi:10.1371/journal.pgen.1010594)
Supplement: S12 Fig — Data availability for measures used in this study are shown for all sequenced samples. Each row represents a pattern of available and missing data, with green squares indicating available data and grey squares indicating missing data. Total counts of available data points for each variable are listed across the top of the figure. Total counts for each data availability pattern are listed along the right. (PDF) [file pgen.1010594.s012.pdf]

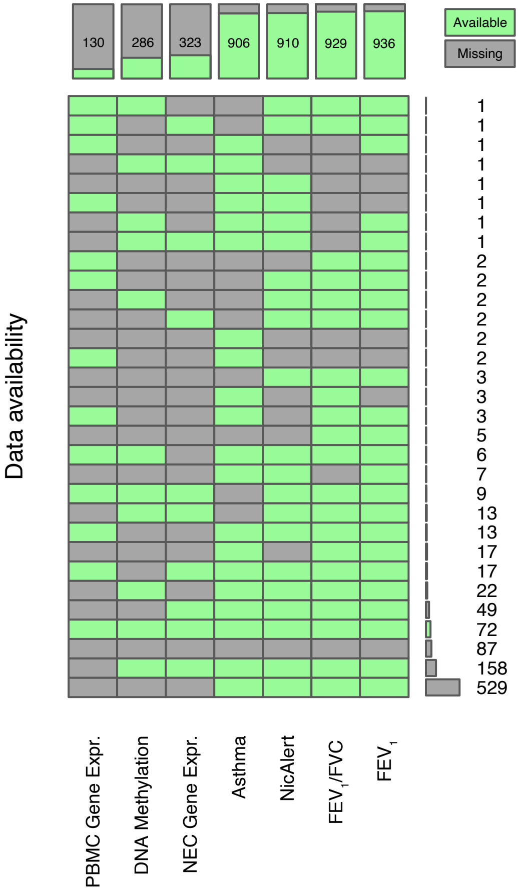

Available
Missing
130
286
323
906
910
929
936
Data availability
1
1
1
1
1
1
1
1
2
2
2
2
2
2
3
3
3
5
6
7
9
13
13
17
17
22
49
72
87
158
529
PBMC Gene Expr.
DNA Methylation
NEC Gene Expr.
Asthma
NicAlert
FEV1/FVC
FEV1
