# Supplementary material for: Multi-omic association study identifies DNA methylation-mediated genotype and smoking exposure effects on lung function in children living in urban settings
Source: PLoS Genet. 2023 Jan 13;19(1):e1010594. doi: 10.1371/journal.pgen.1010594 (PMC9879483; doi:10.1371/journal.pgen.1010594)
Supplement: Attachment — Submitted filename: Dapas_WGS_LungFunction_PLOSGen_ReviewerResponses.docx [file pgen.1010594.s024.docx]

**RESPONSES TO REVIEWER COMMENTS**

We sincerely thank each of the reviewers for their thorough review of our manuscript and for their thoughtful feedback. We have endeavored to address all of their comments in this revision and feel that the manuscript is much improved as a result. Our responses to each comment are provided below with corresponding changes made to the manuscript. The line numbers refer to those in the clean copy of the revised manuscript.

**COMMENTS FROM REVIEWER #1**

Authors looked into an understudied multiethnic pediatric population of sample size of 1035 and discovered one significant locus on 14q32.22 from GWAS of reduced lung function. Authors then moved on to study its functionality through locating its corresponding DNA methylation (which was linked to smoking), followed by additional analyses looking into the gene expression and promoter-enhancer interaction. The manuscript is overall clearly written.

1. It would be helpful to mention the sample size and the study population of Shrine’s meta-analysis study (line 171)

**Response:** We added the names of the cohorts included in the meta-analysis and provided the study population size.

**Changes to manuscript:** The text was revised (lines 184-185):

*“We examined association results for the previously identified FEV­_1_ and FEV_1_/FVC loci reported in the meta-analysis of the UK Biobank and SpiroMeta Consortium by Shrine and colleagues (n=400,102)…”*

2. A fine mapping analysis would be appropriate here to narrow down the causal SNP in the region given the multi-ancestral sample, as authors also pointed out in line 342. The conclusions of the manuscript are mainly based on this SNP rs10220464 which has the highest significance. From Fig 3, there are really quite a few SNPs with similar p-value and highly correlated with this SNP. Is there any public available GWAS dataset for European children?

**Response:** Thank you for this excellent suggestion. We have now conducted a fine-mapping analysis of the *TDRD9* locus using SuSiE [1]. The analysis identified one “credible set” of 59 causal variants, with rs10220464 having the highest individual posterior inclusion probability (PIP) among them (see figure). The relatively small PIP (0.05) is expected due to the large number of SNPs in strong LD and the requirement that the SNP PIPs within a credible set must add up to 1.

There have been other lung function GWAS in children [2, 3], but these studies did not find any genome-wide significant associations with FEV_1_ nor did they make summary statistics publicly available.

**Changes to manuscript:** We added a fine-mapping analysis of the *TDRD9* locus with a corresponding supplementary figure **(S4 Fig)**. In the Discussion, we now call for additional functional studies but not additional fine mapping:

RESULTS (lines 153-155):

*“Fine-mapping analysis at this locus (chr14:103.7-104.3Mb) revealed one 95% credible set of effect variables consisting of 59 SNPs, with rs10220464 having the highest individual posterior inclusion probability among them (****S4 Fig****).”*

DISCUSSION (line 371):

*“Additional ~~fine mapping and/or~~ functional studies are needed to identify the causal variant(s) and full mechanism of action.”*

METHODS (lines 549-553):

*“Fine-mapping analysis was conducted using SuSiE (SusieR R package v0.12.27) [150]. SuSiE applies a form of Bayesian variable selection in regression using iterative Bayesian stepwise selection to identify “credible sets” of variables. Each credible set has a 95% probability of containing at least one causal effect SNP. Prior to running SuSiE, we regressed asthma, age, sex, and ancestry PCs 1-10 from the genotype matrix and outcome vector (the normalized FEV_1_ residuals).”*

3. Rs10220464 was reported to be associated with the methylation at *TDRD9*, *TDRD9* was associated with maternal smoking during pregnancy. Is the SNP reported to be associated with smoking?

**Response:** DNA methylation at the *TDRD9* locus has been found to be associated with maternal smoking [4, 5], but the CpG sites reported in those studies (cg12154261, cg06884495 [Joubert]; cg08933517, cg18991417, cg12154261 [Sikdar]) were not associated with rs10220464 in our study. To our knowledge, no trait association with rs10220464 has been reported previously. The SNP was not associated with smoking exposure in our data.

4. Report the variance explained in stage 1 of the MR model (line 232)

**Response:** The variance explained in Stage 1 of the MR model was r^2^=0.11.

**Changes to manuscript:** We added this value to our Results (lines 244-250):

*“In the first stage, cg03306306 methylation levels in NECs were regressed on an instrument composed of four meQTLs for cg03306306 (rs11160777, rs137961671, rs7143936, rs11160776;* ***Methods****). In the second stage, FEV_1_ was regressed on the predicted DNA methylation values generated from the first stage regression, thereby yielding a causal effect estimate of cg03306306 methylation on FEV_1_. Urine cotinine levels were included as a covariate in both stages. The variance explained in the first stage regression was r^2^=0.11. The causal effect of cg03306306 methylation on FEV_1_ was statistically significant (p=0.020).”*

5. Plotting a graph demonstrated the relationship among leading SNP, CpG site, gene expression, smoking and lung function would be helpful.

**Response:** We added a summary figure (see below) at the end of the Results depicting the various relationships described in our study.

**Changes to manuscript:**

RESULTS (lines 301-304)

*“****Summary of study associations***

*The associations between the TDRD9 risk allele, cg03306306 DNA methylation in NECs, smoking exposure, PPP1R13B gene expression, and FEV_1_ (% predicted) reported in this study are summarized in* ***Fig 9****.”*

6. Report the genetic inflation factor of GWAS study or provide other evidence of the control of population stratification. Are 3 PCs enough to control for population stratification in methylation association analysis, gene expression analysis and mediation analysis for the multiethnic population?

**Response:** The lambda values were 1.02 for both the FEV_1_ and FEV_1_/FVC GWASs, indicating adequate control for population stratification. We have added the QQ plots as a supplementary figure.

Regarding the ancestry PCs, the variance explained by each PC drops and levels off after PC3 (Fig 1B), and the variance among individuals for PCs 4-10 is much smaller relative to PCs 1-3 (Fig 1C). PCs 1-3 appear to capture well the main axes of global ancestry in APIC and URECA. Nonetheless, because the GWASs featured millions of statistical tests in a diverse cohort, we included 10 PCs to reduce potential population stratification. The subsequent methylation and gene expression testing in URECA, however, consisted of fewer tests in a smaller cohort. Therefore, we concluded that including 10 PCs in those tests was unnecessary and could cause overfitting.

**Changes to manuscript:** The genomic control factors are now reported in the Results (lines 144-145) and corresponding QQ plots have been added as a supplementary figure (see below; **S3 Fig**).

*“The genomic control factor, λ_GC_, for both GWAS results was 1.02 (****S3 Fig****), indicating adequate control for population stratification.”*

**COMMENTS FROM REVIEWER #2**

Dapas and coauthors presented an integrative study of lung function in two multi-ethnic cohorts of children living in underprivileged, urban communities by interrogating GWAS, DNA methylation, RNA sequencing, and HiC data. Given that most GWAS of lung functions have focused on adult populations of primarily European ancestry, this study is a timely contribution to the literature and provides unique insights into the genetic, genomic and environmental factors underlying lung function in multi-ethnic, underprivileged children. The manuscript is overall well written and I enjoyed reading it. It is a considerable strength to interrogate DNA methylation and RNAseq in lung function-relevant tissues (upper airway (nasal) epithelial cells (NECs) and lower airway (bronchial) epithelial cells (BECs)), as well as PBMC and blood, which contributes to the novel insights provided by this study. I have some specific comments about the analysis methods and results interpretation as follows.

1. The role of asthma: based on Table 1, one of the two cohorts (APIC) only consisted of children with asthma, while the other cohort is also substantially over-represented by kids with asthma. This reviewer is thus wondering how much the identified signals are attributed to asthma. Also the manuscript seemed inconsistent regarding how asthma was treated in the analyses. For example, it states “we adjusted for asthma status in the GWAS” in line 367, while it states “Individuals without a confirmed asthma status (n=127) were excluded from trait association testing” in line 497. Please clarify.

**Response:** The removal of 127 samples “without a confirmed asthma status” from the trait association testing referred to individuals for whom an asthma diagnosis could not be determined. These individuals were from URECA and were primarily those who dropped out of the study at a young age before an asthma diagnosis could be made. Most of these individuals also did not have FEV_1_ or FEV_1_/FVC measures (**S12 Fig**).

Most of our test population was asthmatic, and the non-asthmatics in URECA were also selected based on family history of asthma or allergies and therefore were also likely to have elevated genetic risk for asthma. Even after controlling for asthma status, it remains possible that the genetic associations reported in our study have smaller effects in populations with lower asthma risk/prevalence. This could potentially explain why the *TDRD9* locus has not been previously identified. This is an important point we address in the Discussion (lines 375-377, 410-418).

To control for asthma’s effect on lung function and thereby reduce attribution of identified signals to asthma, we included asthma as a covariate in our association analyses. However, as Reviewer #3 pointed out, this could have attenuated lung function signals or introduced collider bias. To address this concern, we repeated the lung function GWASs without including asthma as a covariate when normalizing the trait values or testing for genome-wide associations. The results were largely the same, with the *TDRD9* locus remaining the only significant signal (**S5 Fig**).

**Changes to manuscript:** We now report supplementary results of the GWAS without asthma adjustment. We clarified in the Methods that the exclusion of the 127 samples without a confirmed asthma status referred to individuals for whom an asthma diagnosis could not be determined and spirometry was never performed (lines 544-545):

*“Individuals ~~without a confirmed asthma status~~ who were not evaluated for asthma at ages 7 or 10 (n=127) were excluded from trait association testing.”*

2. In addition to Manhattan plots (Fig 2), the authors should present QQ plots as well as genomic control lamba.

**Response:** We now include QQ plots as a supplementary figure (see ***Reviewer 1, Comment 6***). The lambda values were 1.02 for both FEV_1_ and FEV_1_/FVC, indicating adequate control for population stratification.

**Changes to manuscript:** The genomic control factors are now reported in the Results (lines 144-145) and corresponding QQ plots have been added as a supplementary figure (**S3 Fig**).

*“The genomic control factor, λ_GC_, for both GWAS results was 1.02 (****S3 Fig****), indicating adequate control for population stratification.”*

3. For the mediation analysis, could the authors also present proportion (percentage) of total effect mediated by the CpG site methylation?

**Response:** We now report the proportion mediated according to the bootstrapped estimates.

**Changes to manuscript:** In lines 253-256: *“The indirect effect of rs10220464 on FEV_1_ via cg03306306 methylation was significant, both when including asthma as a covariate (β_z_=-0.04, 95% CI=-0.10- -0.003, percent mediated=14.4%) and when asthma was not considered (β_z_=-0.04, 95% CI=-0.10- -0.002, percent mediated=15.0%).”*

4. The lead SNP rs10220464’s MAFs differ across ethnicities according to dbSNP (lower in Blacks and higher Hispanics and Whites). Could the authors report the MAFs for this SNP in the current cohorts by ethnic groups, in particular, in Blacks and Hispanics. Was there indication of genetic effect heterogeneity across Black/Hispanic, asthma/non-asthma, and between the two cohorts?

**Response:** We performed additional association tests for rs10220464 to explore whether there was genetic effect heterogeneity by ancestry, by asthma status, and by study. To visualize effect size differences, we tested the rs10220464 association with FEV_1_ using the same linear mixed model as in the GWASs but limiting the test cohort to each sub-group in turn (e.g. Black non-Hispanics only, Hispanics only, etc.). To statistically test for effect size differences, we utilized the whole APIC & URECA sample and added to our model an interaction term with rs10220464 for each variable of interest (e.g. study).

For ancestry, we performed separate association tests in each of the Black, Hispanic, and white populations, according to self-identified race/ethnicity. We also tested for genotype-by-ancestry interaction effects across the whole APIC & URECA sample by using admixture proportions as covariates in our models, in lieu of ancestry PCs, and including an interaction term for each continental ancestry group and rs10220464 in turn.

For asthma status, we performed separate association tests in the asthmatics and non-asthmatics and also ran the original association test model with the addition of an interaction term between rs10220464 and asthma status.

To determine whether there was effect heterogeneity by study (APIC vs. URECA), we performed separate association tests in each study and we also ran the original association test model with the addition of a study covariate and an interaction term between rs10220464 and study.

None of the interaction effects we tested had a p-value <0.05. Our results did not indicate any heterogeneity by ancestry, asthma status, or study, insofar as we had the power to detect.

**Changes to manuscript:** We now report the genetic effect heterogeneity analysis in the Results (lines 150-156) and added an additional figure (**Fig 4**, see below) showing the subgroup-specific effects:

*“The minor allele at the lead SNP (rs10220464; MAF=0.30) was significantly associated with lower FEV_1_ (p=2.4x10^-9^; β_z_= -0.31, 95% confidence interval (CI)= -0.41- -0.21) and nominally associated with lower FEV_1_/FVC (p=1.1x10^-3^; β_z_= -0.17, 95% CI= -0.28- -0.07). Fine-mapping analysis at this locus (chr14:103.7-104.3Mb) revealed one 95% credible set of effect variables consisting of 59 SNPs, with rs10220464 having the highest individual posterior inclusion probability among them (****S4 Fig****). We did not detect any significant differences in rs10220464 association effect size by ancestry or asthma status or study for FEV_1_ (****Fig 4****).”*

We added a corresponding description of this analysis in the Methods in the ‘Quantitative Trait Association Testing’ section (lines 554-566):

*“To explore whether there was lead-SNP effect heterogeneity by ancestry, study, or asthma status, we performed additional single-SNP quantitative trait association tests within several different sub-cohorts and introduced interaction effects into our models. For ancestry, we performed separate association tests in each of the Black, Hispanic, and white populations, according to self-identified race/ethnicity. We then tested for genotype-by-ancestry interaction effects across APIC and URECA by using admixture proportions as covariates in our models, in lieu of ancestry PCs, and including an interaction term with the lead SNP for each continental ancestry group in turn. We tested these interaction effects using the --gxe argument in GEMMA in four separate models (one for each ancestry). To determine whether there was effect heterogeneity by study (APIC vs. URECA), we performed separate association tests in each study and also tested the association across APIC and URECA with the addition of a study covariate and a genotype-by-study interaction term. For asthma status, we performed separate association tests in the asthmatics and non-asthmatics and tested a genotype interaction term with asthma status.”*

And we added mention of these findings in the Discussion in the context of not having identical samples between analyses (lines 403-407):

*“There are additional caveats to consider when interpreting our findings. First, this study integrated data from two cohorts with different recruitment criteria, asthma definitions, and ancestral compositions. Furthermore, most of the analyses beyond the GWAS were limited to non-identical subsets of URECA participants. However, we did not observe significant genetic effect heterogeneity for rs10220464 by study, asthma status, or ancestry.”*

5. Line 186: please clarify n=82 was the sample size or # of GWAS loci.

**Response:** We rewrote the sentence to clarify that n=82 referred to the number of FEV_1_-associated variants we used to define the *TDRD9* locus.

**Changes to manuscript:** The text was revised in lines 198-201:

*“We tested for associations between the FEV_1_ genotype, as tagged by rs10220464, and DNA methylation levels at 796 CpG sites within 10 kb of ~~the region containing~~ any TDRD9 locus variants associated with FEV_1_ ~~-associated variants (~~at p<1x10^-5^~~,~~ (n=82 variants).”*

6. Lines starting from 228 and lines starting from 525 regarding MR analysis: it seems that the four SNPs/IVs used in the MR analysis were derived from the dataset itself based on association testing, which may suffer from the Winner’s curse. Also were the four selected SNPs used as four IVs or a single IV by constructing a weighted/unweighted genetic score?

**Response:** For the MR analysis to be valid, the instrumental variables must be sufficiently associated with the intermediate factor and not independently associated with the outcome. The instruments were derived from the same dataset as the outcome regression and therefore could suffer from winner’s curse, but MR analysis with weak instruments could also produce bias. We tried to balance these factors by selecting instruments from multiple haplotypes with modest exposure associations that were not associated with FEV_1_ when controlling for the exposure. The four instrumental variables were included as separate individual predictors in our model.

Combining genetic IVs into a single genetic score can help reduce bias from weak instruments [6] and using an unweighted score can reduce bias resulting from winner’s curse [7]. We therefore repeated the MR analysis using an unweighted allele score consisting of the 4 IVs. The resultant causal association p-value was p=0.045.

**Changes to manuscript:** We have added a secondary MR analysis using an unweighted allele score and highlighted additional study limitations in the Discussion, including mentioning the potential bias from the winner’s curse effect.

RESULTS (lines 250-252):

*“The causal effect of cg03306306 methylation on FEV_1_ was statistically significant (p=0.020). We also tested a single, unweighted allele score of the instrumental variables and observed a causal effect association of p=0.045 (stage-one r^2^=0.10).”*

DISCUSSION (lines 381-387):

*“To infer causality, Mendelian randomization and mediation analyses rely on assumptions that are often difficult to empirically verify. For the Mendelian randomization analysis, we identified instrumental variants associated with the intermediate cg03306306 that were not independently associated with the outcome, FEV_1_. However, because these variants were selected from the same dataset that the outcome testing was performed in, they were susceptible to bias from winner’s curse [108]. To mitigate the potential impact from this effect and from weak instruments, we performed a secondary analysis in which we combined the instrumental variants into a single, unweighted score. ”*

METHODS (lines 606-609):

*“We tested two 2SLS models: one where the instrumental variables were included as individual predictors, and another featuring an unweighted allele score of the four instrumental variants to reduce potential bias from weak instruments and/or winner’s curse.”*

7. Data availability: WGS data phs002921.v1.p1 cannot be found on dbGaP. There is no information about where the RNAseq, DNA methylation and HiC array data are deposited for public access.

**Response:** We have since completed the dbGaP submission process for the WGS data under accession phs002921.v1.p1 and are in the process of uploading the GWAS summary statistics. The URECA gene expression data are available on the Gene Expression Omnibus (GEO) under the accession GSE145505 (NECs) and GSE96783 (PBMCs). The pcHI-C data are available in GEO under the reference series GSE152550. The DNA methylation data are in the process of being deposited to GEO to coincide with the publication of another study [8].

**COMMENTS FROM REVIEWER #3**

In this study the authors report on the potential effects of the gene on lung function as well as evidence of mediation via DNA methylation. While a lot of information is presented in support of these findings, I have some methodological concerns regarding the control for asthma status and mediation analysis. Furthermore I am not sure the evidence presented support the potential role attributed to the PPP1R13B gene as a potential part of a causal mechanism.

1. The motivation in the introduction (and the rest of the manuscript) alludes lung development, however the chosen spirometry parameters are FEV1 and the FEV1/FVC. Why was not FVC considered as an outcome? FEV1 and especially the FEV1/FVC ratio are parameters indicative primarily of airway obstruction/restriction. Decreased FVC would likely be a better indication of impaired lung development than at least the FEV1/FVC ratio.

**Response:** We were primarily interested in obstruction and therefore only evaluated FEV_1_ and FEV_1_/FVC. However, we see that the manuscript’s narrative placed too much emphasis on lung development relative to the airways.

**Changes to manuscript:** We have updated the manuscript to allude more to obstruction and airway development, as opposed to lung growth/development, per se:

*“This study reveals a potential mechanism through which genetic risk and environmental exposures can affect ~~lung~~ airway development, perhaps leading to interventions that can help reduce the burden of ~~chronic respiratory diseases~~ asthma in socioeconomically disadvantaged children.”*

*“~~Impairment of lung function~~ Airway obstruction often begins in early life, with lower lung function in infancy being a risk factor for the development of asthma in childhood and COPD in late adulthood.”*

*“Lung function is also affected by environmental exposures, such as smoking and air pollution, which can disrupt ~~lung growth and~~ airway development in early life, increasing the risk of childhood asthma and perhaps other chronic obstructive ~~respiratory~~ diseases.”*

*“The specific function(s) of this gene in the airway epithelium and its potential impact on ~~lung~~ the development of airway obstruction remain to be elucidated.”*

*“…additional study of PPP1R13B in lung and airway development is warranted, particularly in the context of adverse environmental stimuli…”*

*“Our study identified a novel avenue through which genetic risk and environmental exposures could affect ~~lung development in~~ the airways of children…”*

2. The adjustment for asthma status particularly for the potential genetic effects is problematic. It is mentioned as part of a set of 'confounding factors', however it the structural sense of confounding it is not one. It can however be considered as an intermediate as it is likely caused by some genetic factors and in turn affects the outcome (lung function parameters). Its adjustment therefore is problematic as it will remove part of the potential effect of exposure, but also introduce the potential for collider bias if unmeasured common causes of asthma status and lung function exist. The two-stage approach for GWAS using the normalized residuals of the outcome does not protect from this issue. It was reassuring to see that the reported effect for the highlighted gene remained even without adjustment for asthma, but adjustment may have obscured effects of other genes and would also cause problems in the mediation analysis in quantification of direct and indirect effects.

**Response:** Thank you for this comment. Because asthma often leads to lower function in children [9] (**S2 Fig**), we considered that it would be problematic *not* to adjust for asthma in a population with high asthma prevalence if the goal is to identify lung function genes. Any corresponding association signal may be attributed to asthma risk and not necessarily lung function itself. However, we also recognize that adjusting for asthma could nullify some true lung function association signals, but we wanted to avoid detecting associations that were primarily disease-induced. Nonetheless, to more thoroughly evaluate the effects of the asthma adjustment, we repeated the lung function GWASs without adjusting for asthma in either stage. The *TDRD9* association signal for FEV_1_ remained the only significant association in our study, with rs10220464 as the lead SNP with p=2.8x10^-9^. The beta correlations between each pair of GWAS was 0.981 for FEV_1_ and 0.954 for FEV_1_/FVC.

We also tested the effect of removing asthma as a covariate in the mediation analysis. We used the normalized FEV_1_ residuals (see Response to ***Reviewer 3,*** ***Comment 4***) that were not adjusted for asthma, and we did not include asthma as a covariate in the mediation test. The results remained significant (β_z_=-0.04, 95% CI=-0.10- -0.002).

**Changes to manuscript:** We added a supplementary figure (**S5 Fig**, see below) showing the GWAS results without adjusting for asthma status and made the following changes in the manuscript:

RESULTS

(lines 155-159)

*“We did not detect any significant differences in rs10220464 association effect size by ancestry or asthma status or study for FEV_1_ (****Fig 4****). Furthermore, the TDRD9 locus remained the only genome-wide significant association when the GWAS were performed without adjustment for asthma status (****S5 Fig****). The overall effect size correlations between asthma-adjusted and unadjusted GWAS results were r=0.981 for FEV_1_ and r=0.954 for FEV_1_/FVC. ”*

(lines 252-256)

*“We further performed a bootstrapped mediation analysis to test whether the rs10220464 risk allele effect on FEV_1_ was mediated by DNA methylation. The indirect effect of rs10220464 on FEV_1_ via cg03306306 methylation was significant, both when including asthma as a covariate (β_z_=-0.04, 95% CI=-0.10- -0.003, percent mediated=14.4%) and when asthma was not considered (β_z_=-0.04, 95% CI=-0.10- -0.002, percent mediated=15.0).”*

DISCUSSION (lines 410-418)

*“Because children with asthma have lower lung function overall (****Table 1****) and their lung function ~~is~~ may be more affected by ~~certain~~ environmental exposures [108-110], we adjusted for asthma status in the GWAS, as ~~others have done previously~~ in previous GWAS [111-114]. The likelihood of discovering lung function variants with consistent effects in asthmatics and non-asthmatics was thereby increased, although genetic determinants of lung function may differ by asthma status [115]. Furthermore, adjusting for disease status could potentially introduce collider bias [116]. The significant genotype effect at the TDRD9 locus, however, remained the only genome-wide-significant association when asthma was excluded as a covariate, and adjustment for asthma did not substantively alter the mediation results.”*

METHODS

(lines 538-548)

*“Quantitative traits were adjusted for ~~confounding factors~~ covariates and normalized using a two-stage approach … To identify potential collider bias introduced by adjusting for asthma status, we repeated the GWAS without accounting for asthma status in either covariate-adjustment stage.”*

(lines 612-614)

*“Age at FEV­1 measurement, sex, asthma status, ancestry PCs 1-3, and urine cotinine levels were included as covariates. We also performed a secondary mediation analysis without adjusting for asthma status.”*

3. The mediation results presented here do not refer to any of the assumptions required for mediation analysis or the assumptions under which mediated effects are estimated. Mediation analysis requires exchangeability assumptions that essentially require no unmeasured exposure-outcome, exposure-mediator and mediator-outcome confounding. The latter assumption in particular is the more problematic one as in this case the only other common cause of methylation and outcome considered is tobacco exposure (presumably from current SHS exposure). Any other environmental risk factor affecting both DNA methylation at the outcome would mean this assumption fails. Importantly the authors have provided enough evidence of mediation even in light of these limitations: The meQTL results for exposure-mediator relationship are in theory not thought to be very prone to confounding, while the instrumental variable approach for the mediator-outcome relationship is considered valid even in the presence of unmeasured confounding between methylation and the outcome if the instrument is valid. Both these relationships have a causal interpretation and together are sufficient to indicate mediation. The quantification of direct and indirect effects, however, is dependent on the aforementioned assumptions that are not even mentioned let alone addressed in any way. Proper quantification of direct and indirect effects should also take into account potential interactions between exposure and mediator if any exist, which I don’t think the ROBMED package does.

**Response:** To identify any potential confounding that could affect our mediation analysis and to address this comment, we systematically tested for associations with 2 socioeconomic variables and 11 environmental exposures (see below) that were available in the APIC and/or URECA cohorts.

For each environmental exposure, we tested whether the variable was associated with the mediator (CpG) and whether the variable was associated with the outcome (FEV_1_) conditional on the mediator. Associations in both tests would indicate mediator-outcome confounding. In each regression model we included the standard set of covariates used in the mediation analysis and additionally adjusted for the participant age at variable measure where applicable. At an association p-value threshold of 0.05 we did not detect any relationships indicative of confounding.

The ROBMED package does not model potential interactions between exposure and mediator, but when we added rs10220464 and a CpG-SNP interaction term to our CpG-FEV_1_ association test we did not observe an interaction effect.

**Changes to manuscript:** We added a description of this analysis to the Methods and added a discussion around MR/Mediation assumptions to the Discussion.

DISCUSSION (lines 381-394):

*“To infer causality, Mendelian randomization and mediation analyses rely on assumptions that are often difficult to empirically verify. For the Mendelian randomization analysis, we identified instrumental variants associated with the intermediate cg03306306 that were not independently associated with the outcome, FEV_1_. However, because these variants were selected from the same dataset that the outcome testing was performed in, they were susceptible to bias from winner’s curse [108]. To mitigate the potential impact from this effect and from weak instruments, we performed a secondary analysis in which we combined the instrumental variants into a single, unweighted score. For the mediation analysis, unmeasured confounding can invalidate direct and indirect effect estimates [109]. To protect against such bias, we systematically tested for confounding associations with additional environmental measures available in APIC and URECA (****Materials and*** ***Methods****). Nonetheless, there may still exist unknown confounding factors that were not measured. Ultimately the results of the Mendelian randomization and mediation analyses indicate~~d~~ that methylation at cg03306306 in NECs mediated the rs10220464 genotype effect on FEV_1_, but there was residual correlation between rs10220464 and FEV_1_, signifying that the genotype effect was only partially mediated by cg03306306.”*

METHODS (lines 610-621):

*“Mediation analysis was conducted with ROBMED [153]. The adjusted and normalized FEV_1_ residuals were set as the dependent variable, adjusted cg03306306 methylation as the mediator, and rs10220464 as the independent variable. Age at FEV­1 measurement, sex, asthma status, ancestry PCs 1-3, and urine cotinine levels were included as covariates. We also performed a secondary mediation analysis without adjusting for asthma status. To identify additional, potential confounders that could invalidate our mediation model, we systematically tested for associations with 2 socioeconomic variables and 11 environmental exposures available in APIC and URECA (****S8 Table****,* ***S15 Fig****). For each environmental exposure, we tested whether the variable was associated with the mediator (cg03306306) and whether the variable was associated with the outcome (FEV_1_) conditional on the mediator. To ensure no exposure-mediator interactions, we repeated the cg03306306 association test with FEV_1_ with rs10220464 included as a predictor with a rs10220464: cg03306306 interaction term. The indirect effect of rs10220464 on FEV_1_ via cg03306306 methylation was estimated using 100,000 bootstrap resamples.”*

4. Furthermore, since indirect effects are reported it is very difficult to compare to the total effects (assuming they are accurate if the assumptions above hold). Based on the betas reported, the indirect effect is greater than the total effect which would mean that the direct effect is in the opposite direction. Based on my understanding of the methods however, the betas aren't comparable (one is based on normalized residuals of the outcome, the other is FEV1 predicted untransformed). It the results are to be kept then it would greatly benefit the reader to see them in a scale that is comparable.

**Response:** To better compare the total and indirect effect sizes we replaced the untransformed FEV_1_ values in the mediation analysis with the normalized FEV_1_ residuals and report the effects in standardized units. These results better illustrate how cg03306306 only partially mediates the rs10220464 risk allele effect on lung function.

**Changes to manuscript:** *“Mediation analysis was conducted with ROBMED. The adjusted and normalized FEV_1_ ~~(% predicted)~~ residuals ~~was~~ were set as the dependent variable, adjusted cg03306306 methylation as the mediator, and rs10220464 as the independent variable.”*

5. Identifiability assumptions and especially the issue of exchangeability also affects the issue of interactions. Identification of interactions in epidemiology both implies the presence of causal effects of the two (or more) variables of interest potentially interacting to jointly affect an outcome, but also that the causal effects of both of exposures are identifiable. This becomes an issue with (secondhand) smoking exposure especially as no socioeconomic indicators and co-exposures are adjusted for.

**Response:** The range of socioeconomic status in this study was relatively narrow. The study participants were selected based on neighborhood poverty rates and so their incomes were uniformly low. For example, 54% of APIC and URECA had family income levels <$15K. We would therefore expect to observe less of a correlation between smoking and socioeconomic indicators in this cohort relative to a broader population sample.

The only direct socioeconomic variables available to us were whether a participant’s family had a total annual income <$15K and whether the caretaker had completed high school. We investigated these relationships with cotinine levels further. The correlation between the NicAlert test results and the income<$15k variable was 0.05, with a p-value of 0.15 (see below).

The correlation between the NicAlert test results and the caretaker-completed-high-school variable was 0.06, with a p-value of 0.07 (see below).

We added these variables as a covariate in our interaction models to see if doing so affected our interaction effect estimates, but we did not observe any change in the smoking interaction effects with DNA methylation or rs10220464 genotype.

6. The focus on the PPP1R13B gene as a part of a potential causal mechanism for decreased lung function is problematic in the sense that it is not associated with the outcome of interest. If it was indeed part of the causal mechanisms then it should be associated with lung function parameters, while the proposed roles in the discussion would also lead us to expect that it should be associated with the outcome. The association with the risk alleles and DNA methylation alone does not necessarily implicate this gene on effects on lung function as the authors assert in the title and the discussion. In this sense the importance of this particular finding is overstated in the article.

**Response:** We recognized that *PPP1R13B* expression in NECs at age 11 was not significantly associated with FEV_1_ at ages 5-10 (**S6 Table**) in the 299 URECA children included in that model. We weighed that evidence against the strong eQTL association, the methylation correlation, the pcHi-C results, and the purported *PPP1R13B* function and felt that the evidence overall implicated the *PPP1R13B* gene. The word ‘implicate’ is a subjective term that implies an overall preponderance of evidence, although it may not have been conservative enough in this case.

Within the Discussion, we laid out both sides and provided an ultimate interpretation that we feel is fair, recommending further study of the gene in this context:

*“PPP1R13B expression in airway epithelial cells at age 11 was not directly associated with lung function or urine cotinine levels in the URECA children, but ~~its~~ the cofactors of this gene [79,91] have been found previously to be upregulated in smokers with COPD [81,92]. Given its association with lung function alleles in our study, its expression in the ~~lung~~ airway epithelium, and its purported functions in autophagy and apoptosis pathways, additional study of PPP1R13B in lung and airway development is warranted, particularly in the context of adverse environmental stimuli, many of which are enriched in low-income urban environments.”*

Nonetheless, this more nuanced interpretation, which lies within the Discussion, was perhaps overshadowed by stating the gene’s implication within the article title.

**Changes to manuscript:** We altered the title to remove mention of *PPP1R13B* and we modified the wording of our final conclusions in the abstract and at the end of the Discussion.

***“Multi-omic association study ~~implicates PPP1R13B in~~ identifies DNA methylation-mediated genotype and smoking exposure effects on lung function in children living in urban settings”***

ABSTRACT (lines 48-49):

*“These combined results highlight a potential novel mechanism for reduced lung function in urban youth ~~through increased airway epithelial apoptosis~~ resulting from both genetics and smoking exposure.”*

DISCUSSION (lines 428-430):

*“Our study identified a novel avenue through which genetic risk and environmental exposures ~~can~~ could affect the airways of children raised in low-income urban neighborhoods. Further research into this pathway may yield mechanistic insights…”*

In the newly added summary figure (**Fig 9**), in response to ***Reviewer 1 Comment 5***, we do not directly illustrate a relationship between *PPP1R13B* and FEV_1_.

7. Lastly, since most of the remaining analysis beyond the GWAS is only performed in URECA it would be interesting to see if the primary finding for TDRD9 persists in the URECA sample alone. A lot of the additional results are only based on URECA participants and even if the effect is still present but with a different magnitude it makes it difficult to compare total and indirect effects coming from only moderately overlapping samples.

**Response:** We performed additional association tests for rs10220464 to explore whether there was genetic effect heterogeneity by ancestry, by asthma status, and by study (see ***Reviewer 2, Comment 4***). This included running separate association tests in APIC & URECA. We added a new figure (**Fig 4**) showing rs10220464 effect sizes in different sub-cohorts, including in URECA alone (p=1.5x10^-3^; β_z_= -0.23, 95% CI= -0.37- -0.09). We did not observe significant effect heterogeneity by any of the variables we explored, including study, but the effect size for rs10220464 was slightly lower in magnitude in URECA relative to APIC. To help provide perspective on which individuals were included in each analysis, we also added another supplementary figure (**S12 Fig**) showing a histogram of data availability for the variables analyzed in our paper.

**Changes to manuscript:** We added a genetic effect heterogeneity analysis for rs10220464 (**Fig 4**). We also added a supplementary figure showing a histogram of data availability in APIC & URECA. We also added a diagram showing the URECA participant overlap between various analyses to help provide perspective **(S12 Fig**, see below). Finally, we added mention of this consideration in the Discussion.

DISCUSSION (lines 403-407):

*“There are additional caveats to consider when interpreting our findings. First, this study integrated data from two cohorts with different recruitment criteria, asthma definitions, and ancestral compositions. Furthermore, most of the analyses beyond the GWAS were limited to subsets of the URECA participants. However, we did not observe significant genetic effect heterogeneity for rs10220464 by study, asthma status, or ancestry.”*

METHODS (lines 461-463):

*“This study utilized DNA methylation and RNA-seq data generated for other URECA studies; therefore, the number of samples included in each analysis varied and was limited by data availability (****S7 Table****,* ***S12 Fig****).”*

8. The authors use the general term of "smoking exposures" though given the age of the children in URECA this is likely secondhand smoke exposures. This should be better specified in the text.

**Response:** We intentionally used the term “environmental tobacco smoke exposure”, or “smoking exposure” for short, since this was measured using cotinine assays with no further regard for the specific avenues of exposure. Indeed, for URECA these exposures are likely due to secondhand smoke, since these measures were mostly taken at age 10, but some of the APIC participants were older, with 50 participants aged 16 or 17. The distribution of NicAlert results (**S6 Fig**) indicates that some of the participants were likely smokers themselves.

The terms “environmental smoking exposure” or “smoking exposure” connote secondhand smoke but also do not preclude direct smoking, and because we could not determine the actual avenues of smoking exposure, we felt that these terms were more appropriate to use throughout. However, to clarify that most tobacco exposures were likely due to secondhand smoke, we added this point to the Results.

**Changes to manuscript:** Lines 239-240: *“There was modest evidence for a genotype-by-smoking exposure interaction effect on FEV_1_ in the combined APIC and URECA sample, but this did not reach statistical significance (p=0.06,* ***S10 Fig****). Considering the ages of the participants in APIC and URECA, most tobacco exposures were likely due to secondhand smoke.”*

**REFERENCES**

1.	Wang G, Sarkar A, Carbonetto P, Stephens M. A simple new approach to variable selection in regression, with application to genetic fine mapping. Journal of the Royal Statistical Society: Series B (Statistical Methodology). 2020;82(5):1273-300. doi: <https://doi.org/10.1111/rssb.12388>.

2.	Goddard PC, Keys KL, Mak ACY, Lee EY, Liu AK, Samedy-Bates LA, et al. Integrative genomic analysis in African American children with asthma finds three novel loci associated with lung function. Genet Epidemiol. 2021;45(2):190-208. Epub 20200929. doi: 10.1002/gepi.22365. PubMed PMID: 32989782; PubMed Central PMCID: PMCPMC7902343.

3.	Ong BA, Li J, McDonough JM, Wei Z, Kim C, Chiavacci R, et al. Gene network analysis in a pediatric cohort identifies novel lung function genes. PLoS One. 2013;8(9):e72899. Epub 20130902. doi: 10.1371/journal.pone.0072899. PubMed PMID: 24023788; PubMed Central PMCID: PMCPMC3759429.

4.	Joubert BR, Felix JF, Yousefi P, Bakulski KM, Just AC, Breton C, et al. DNA Methylation in Newborns and Maternal Smoking in Pregnancy: Genome-wide Consortium Meta-analysis. Am J Hum Genet. 2016;98(4):680-96. Epub 2016/04/05. doi: 10.1016/j.ajhg.2016.02.019. PubMed PMID: 27040690; PubMed Central PMCID: PMCPMC4833289.

5.	Sikdar S, Joehanes R, Joubert BR, Xu CJ, Vives-Usano M, Rezwan FI, et al. Comparison of smoking-related DNA methylation between newborns from prenatal exposure and adults from personal smoking. Epigenomics. 2019;11(13):1487-500. Epub 2019/09/20. doi: 10.2217/epi-2019-0066. PubMed PMID: 31536415; PubMed Central PMCID: PMCPMC6836223.

6.	Burgess S, Small DS, Thompson SG. A review of instrumental variable estimators for Mendelian randomization. Stat Methods Med Res. 2017;26(5):2333-55. doi: 10.1177/0962280215597579. PubMed PMID: WOS:000412931500022.

7.	Burgess S, Thompson SG. Use of allele scores as instrumental variables for Mendelian randomization. International Journal of Epidemiology. 2013;42(4):1134-44. doi: 10.1093/ije/dyt093. PubMed PMID: WOS:000325167800030.

8.	Morin A, Thompson EE, Helling BA, Shorey-Kendrick LE, Faber P, Gebretsadik T, et al. A Functional Genomics Pipeline to Identify High-Value Asthma and Allergy CpGs in the Human Methylome. medRxiv. 2022:2022.05.19.22275204. doi: 10.1101/2022.05.19.22275204.

9.	Mahut B, Bokov P, Beydon N, Delclaux C. Longitudinal assessment of loss and gain of lung function in childhood asthma. J Asthma. 2022:1-8. Epub 20220103. doi: 10.1080/02770903.2021.2023176. PubMed PMID: 34958615.
