# Supplementary material for: Multi-omic association study identifies DNA methylation-mediated genotype and smoking exposure effects on lung function in children living in urban settings
Source: PLoS Genet. 2023 Jan 13;19(1):e1010594. doi: 10.1371/journal.pgen.1010594 (PMC9879483; doi:10.1371/journal.pgen.1010594)
Supplement: Attachment — Submitted filename: Dapas_WGS_LungFunction_PLOSGen_ReviewerResponses2.docx [file pgen.1010594.s025.docx]

**RESPONSES TO REVIEWER COMMENTS**

We are grateful to each of the reviewers for their thorough review of our manuscript. The additional comments from the latest review helped to improve the manuscript further. Our responses to each of these comments are provided below with corresponding changes made to the manuscript.

**COMMENTS FROM REVIEWER #1**

The authors have addressed all my concerns, well done!

**COMMENTS FROM REVIEWER #2**

This reviewer thanks the authors for addressing all the comments.

**COMMENTS FROM REVIEWER #3**

The authors have addressed comments from the initial review. I have some lingering concerns from the point of view of clarity/accuracy regarding some language used in the Discussion:

1. In the discussion (ln 338) the authors mention that PPP1R13B expression is not 'directly' associated with lung function. The word directly here is problematic in two ways. Terms 'direct' and 'indirect' in epidemiology are usually used to distinguish (causal) effects with respect to a specific mediator of interest which is not the case here as far as PPP1R13B expression and lung function are concerned. The presumed 'indirect association' alluded to here probably has to do with common causes of both PPP1R13B expression and lung function with respect to associated genes and DNA methylation, but absent any causal effect between PPP1R13B expression and lung function for which we have no evidence here, any such association would be by definition non-causal. However, since no association (causal/mediated, or non-causal) between PPP1R13B expression and lung function was reported here I think the sentence should be phrased as "PPP1R13B expression in airway epithelial cells at age 11 was not associated with lung function or urine cotinine levels in the URECA children, but ..." with the word directly entirely omitted.

**Response:** Thank you for this comment. We removed the word from the sentence as suggested.

**Changes to manuscript:**

*“PPP1R13B expression in airway epithelial cells at age 11 was not ~~directly~~ associated with lung function or urine cotinine levels in the URECA children, but…”*

2. In the same page the beginning of the last paragraph (lns 356-357) which reads "The correlations of FEV1 and rs10220464 with cg03306306 methylation and PPP1R13B expression in NECs..." reads as if FEV1 and rs10220464 and are correlated with both cg03306306 methylation and PPP1R13B expression in NECs, however this is not true based on the reported results, as FEV1 was not correlated with PPP1R13B expression in either NECs or PMBCs. This sentence should be restructured for clarification.

**Response:** Thank you for noticing this. We restructured the sentence for clarification. We no longer mention *PPP1R13B* expression, since the paragraph is really about DNA methylation, but we added mention of the smoking exposure association, since cotinine levels were also associated with cg03306306 in NECs but not PBMCs.

**Changes to manuscript:**

*“The correlations of ~~FEV~~_~~1~~_ ~~and~~ rs10220464, FEV_1_, and smoking exposure with cg03306306 methylation ~~and PPP1R13B~~* *~~expression~~ in NECs were absent in PBMCs.”*

3. In the Discussion it is also stated the paragraph regarding interactions also can be misleading and lacks clarity. The paragraph begins (ln 372) by stating "If increased methylation at cg03306306 leads to lower FEV1 in response to environmental stressors, one might expect to see interaction effects with smoking exposure on FEV1". It is not clear what interaction is referred to here. One between methylation and smoking exposures or one between genes and smoking exposure? Regardless, I fear that the sentence is misleading in the sense that evidence of an established relationship between an exposure and mediator or two exposures sharing the same mechanism of effect as is the presumed case here with genetics and smoking exposures both affecting lung function through DNA methylation is by no means enough evidence to lead us to expect the presence of an interaction. The authors are correct in checking for any interaction, however the phrasing as is implies that the evidence at hand would lead us to believe that there might be an interaction, but this is not methodologically sound reasoning and should be rephrased.

**Response:** We appreciate the comment. We replaced the sentence in question to avoid the implication.

**Changes to manuscript:** We removed the supposition at the beginning of the paragraph and adjusted the following sentence accordingly:

*“~~If increased methylation at cg03306306 leads to lower FEV~~_~~1~~_ ~~in response to environmental stressors, one might expect to see interaction effects with smoking exposure on FEV~~_~~1~~_~~.~~ We tested for interactions between smoking exposure and rs10220464 genotype effects on cg03306306 and on FEV_1_ and between smoking exposure and cg03306306 methylation effects on FEV_1_. We did not detect any ~~such~~ significant interactions, ~~although~~ but our analyses in that regard ~~were likely~~ could have been underpowered given our observed effects and sample sizes [36]. Furthermore…”*

We also moved the preceding paragraph (*“The FEV_1_ association signal…”*) up in the Discussion to improve the topical flow (swapping Discussion paragraphs #4 and #5). The paragraph in question here (paragraph #6) now immediately follows the one discussing DNA methylation in NECs vs. PBMCs; Comment #2).
